# Changes in sex distribution in Achilles tendon rupture literature over 74 years: A systematic review

**DOI:** 10.1002/ksa.70520

**Published:** 2026-07-06

**Authors:** Morgan N. Potter, Jenna Mlecko, Marianne Christensen, Susanna Aufwerber, Sarah E. Katz, Ryan T. Pohlig, Karin Grävare Silbernagel

**Affiliations:** ^1^ Department of Physical Therapy University of Delaware Newark Delaware USA; ^2^ Physiotherapy and Occupational Therapy Aalborg University Hospital Aalborg Denmark; ^3^ Department of Orthopaedic Surgery Aalborg University Hospital Aalborg Denmark; ^4^ Department of Molecular Medicine and Surgery Karolinska Institute Stockholm Sweden; ^5^ Women's Health and Allied Health Professionals Theme, Medical Unity of Occupational Therapy and Physiotherapy Karolinska University Hospital Stockholm Sweden; ^6^ Library, Museums, and Press University of Delaware Newark Delaware USA; ^7^ Department of Epidemiology University of Delaware Newark Delaware USA

**Keywords:** men, sex differences, sex‐disaggregated data, tendon injury, women

## Abstract

**Purpose:**

Evaluate changes in sex distribution within the Achilles tendon rupture (ATR) literature over time.

**Methods:**

Four databases were searched (PubMed, Cochrane Central Register of Controlled Trials, SPORTDiscus and Web of Science). To be included, studies had to evaluate primary and complete ATR and report the years in which participants were enrolled, or injury occurred. A weighted linear regression evaluated the relationship between the article midpoint year (of participant enrolment) and the percentage of females across articles.

**Results:**

In total, 532 articles were included. The midpoint year of participant enrolment ranged from 1948 to 2022. The midpoint year of study enrolment was related to the percentage of females included (*b* = 0.386, *p* < 0.001, *R* ^2^ = 0.073). The average sex distribution across studies (spanning 74 years) was 81 ± 12% male and 19 ± 12% female. The lowest percentage of females was 7% in 1990, and the highest percentage of females was 39% in 2015. Finally, 10.7% (57/532) of articles reported sex disaggregated data for both demographics and variable(s) of interest.

**Conclusions:**

The sex distribution in the ATR literature demonstrates a gradual rise in the percentage of females over time, but males continue to make up a majority of this population. The paucity of sex‐disaggregated data or analyses limited the evaluation of trends among females specifically and limits insight into sex‐specific patterns.

**Level of Evidence:**

Level III, systematic review (includes studies from I to III).

**Abbreviations**
ATR: Achilles tendon rupture
CENTRAL: Cochrane Central Register of Controlled Trials
IRB: Institution Review Board
NCAA: National Collegiate Athletic Association
PRISMA: Preferred Reporting Items for Systematic reviews and Meta‐Analyses

## INTRODUCTION

Achilles tendon rupture (ATR) is a complete tear of the Achilles tendon, typically occurring during sudden ankle dorsiflexion while the knee is in full extension [13]. ATR is most common in active individuals between the ages of 30 and 49 years, with most injuries occurring during sport or recreational activities [18]. Historically, the literature has reported that ATR is more common among males, with only 20%–25% of study samples being female [5, 18]. The reason for the reported greater occurrence in males is not fully understood, but it has been suggested that higher sports participation [7, 23], a greater tendency to be a ‘weekend warrior’ [18] and/or generally stiffer tendons may place males at a greater risk for ATR [12].

Studies from countries across the world show a rise in ATR rates over the past 45 years [14, 17, 18, 20]. This rise is attributed to increased sport participation and a more active ageing population than in previous decades [14, 17, 18]. Despite this well‐documented rise in ATR rates, it is not well understood whether this rise in ATR rates is similar for both males and females. It is possible that the incidence rate among females is rising at a faster pace than among males, as there has been a continued and substantial rise in female sports participation across all levels of competition over the past 50 years [30]. Specifically, female participation in high school sports increased from 24.2% in 1973 to 42.9% in 2018 [30], and the 2024 Paris Olympics was the first Olympic Games to have the same number of male and female athletes [31]. Additionally, in Achilles tendinopathy, another common Achilles tendon injury, there has been a shift from occurring predominantly in males to now having an equal distribution between males and females [29].

Large epidemiology studies of all ages and activity participation have documented a rise in females diagnosed with ATR [7, 14, 16, 17, 18, 20]. Interestingly, a 2019 article by Chan et al. [6] found that in National Collegiate Athletic Association (NCAA) collegiate sports, women's gymnastics had the highest rate of reported Achilles tendon injuries (ruptures and tendinopathy combined) [6]. A study from 2022 found the prevalence of ATR in female collegiate gymnasts was 17.2% [3]. Yet, the literature overall is mixed on whether the rise is greater among males versus females, and if this trend is true across different age groups [7, 14, 16, 17, 18, 20]. There are also shortcomings of these studies as they are typically limited to samples from individual countries and span five to fifteen years. As a result, only relatively small snapshots of the trends in the incidence rate have been captured [7, 14, 16, 17, 18, 20]. A review by Ho et al. [12] evaluated global trends in the sex distribution of ATR and found the percentage of females with ATR in the literature increased by 0.6% every 5 years from approximately 1970 to 2015 [12]. This study provides a great initial step towards a better understanding of global trends of the sex distribution in ATR from 1970 to 2015. However, the exclusion of large datasets and specific populations, such as diabetics or the elderly (not defined), results in a partial understanding of changes in these global trends. A more comprehensive evaluation of global trends is required to establish if a shift in the sex distribution in ATR exists, which in turn could guide future research needs. Therefore, the primary aim of the current systematic review was to determine if the reported sex distribution of study participants with primary ATR in the literature has changed over time.

## METHODS

### Search strategy

The protocol for this systematic review is registered in the International Prospective Register of Systematic Reviews (PROSPERO) under ID: CRD420251006811. The search strategy, developed by a health science librarian (SEK) and in line with the Preferred Reporting Items for Systematic reviews and Meta‐Analyses (PRISMA) guidelines, was initially conducted on 28 July 2023, and included publications from inception through July 2023. The search was then updated on 13 August 2024 to include publications from 1 July 2023 through 1 August 2024. Four bibliographic databases, PubMed, SPORTDiscus, Cochrane Central Register of Controlled Trials (CENTRAL) and Web of Science, were searched, and handsearching was conducted to identify additional eligible studies. The search was developed in PubMed utilizing keywords and controlled vocabulary (e.g., MeSH) and modified for all other databases. The search was limited to English language results and excluded articles about animals, cells, or cadavers. The full search strategies for each database can be found in Table S1.

### Eligibility, screening and assessment of agreement

Inclusion criteria for this systematic review were original research articles published in English that reported on participants with primary, complete, and closed ATR. As the predictor variable was time, articles were required to report the year(s) of participant enrolment (i.e., prospective studies) or injury occurrence (i.e., retrospective reviews). Studies including participants of only one sex were eligible for inclusion as they provide a meaningful contribution to the estimation of sex distribution in the literature over time, which was the primary aim of this systematic review. However, studies in which sex was explicitly or implicitly restricted (i.e., through the study's eligibility criteria and evaluation of sex‐specific sports leagues) were excluded from this systematic review, as these designs would bias the estimation of sex distribution over time. The full exclusion criteria are listed in Table 1.

**Table 1:** Exclusion criteria for the systematic review.

Study exclusion criteria
Not in English
Duplicate article
Animal or cadaver study
Not Achilles tendon rupture *(i.e., different injury, includes ruptures/injuries from various body regions, partial or re‐rupture of Achilles tendon, rupture occurred following use of fluroquinolone)*
Case Report (≤3 people)
Review Paper, Opinion Paper or Book Chapter
Systematic Review or Meta‐analysis
Conference Abstract only available
Excludes one sex
Overlapping/same sample as another included study in review
Participant enrolment years not reported

One reviewer (M.N.P.) screened all titles and abstracts for eligibility. If there was insufficient information in the title or abstract, the article was retained to be further evaluated in the full‐text review. Full‐text review/determination of article eligibility was performed by four reviewers in two groups (Group 1: MNP and JM; Group 2: MC and SA) to minimize erroneous exclusion. All reviewers were trained on the eligibility criteria and were required to demonstrate at least 80% agreement in their practice reviews. If agreement on eligibility could not be reached in one group, the other group was consulted. Any persisting disagreement was ultimately resolved by the senior author (K.G.S.). Authors of articles were contacted when the eligibility of the article could not be determined to obtain additional information.

To minimize the chance of including the same participants multiple times from different published articles, all articles were further reviewed for participant overlap. During the full‐text review, reviewers documented the first author's name and affiliation(s) and, if available, institution review board (IRB) number, clinical trial number and any descriptors of their study sample. Descriptors of overlap include direct citation/description of an earlier publication with the same sample, reference to a future publication that plans to include the same sample, or collection from a widely used or national patient database (i.e., PearlDiver Patient Record Database and Danish Achilles Tendon Database). One reviewer (J.M.) then used Excel to identify publications with the same IRB number, clinical trial number or overlapping descriptors. All identified publications were formally assessed for participant overlap by J.M. based on direct citations/references, recruitment years, recruitment source, sample size, methodology, affiliation and authors. If publications had overlapping participant samples who were recruited over the same years, then the publication with the largest sample size was included, and all others were excluded. If participant samples only partially overlapped in recruitment years, then the combination of articles that provided the largest total sample size, while avoiding any overlap in recruitment dates, was included, and all others were excluded (e.g., If one article recruited participants from 2014 to 2018, one from 2010 to 2015, and the other from 2016 to 2020, then the 2010–2015 and 2016–2020 articles could be included as they do not overlap and only 2014–2018 would need to be excluded).

### Data extraction

The same two groups independently performed data extraction on all articles. A third group of reviewers (J.O. and S.H. assisted with extracting data on a smaller portion of articles (n = 96). This group was also trained on the data extraction procedures and was required to have a minimum of 80% agreement with M.N.P. prior to beginning data extraction. This group was added to ensure the timely completion of the systematic review, given the large volume of articles. Each group evaluated agreement on their respective articles to ensure accuracy. Any disagreements were discussed within the group, and other groups were consulted if agreement could not be reached. The senior author (K.G.S.) was the tie breaker if agreement could not be reached. The data extracted included: year published, year participant enrolment began and completed, study type, country where the study took place, total sample size, number of males and number of females. The percentage of males and females was calculated based on extracted data. Mean age and age range for the whole sample were recorded when available. If the article provided the sex‐disaggregated mean age or age range, it was documented, and those values were recorded. Consistent with a previous systematic review [29], it was documented whether an article provided sex disaggregated data in demographics/descriptives, variable(s) of interest, or both. It was also recorded if a study did not provide sex disaggregated data but did report a significant finding of sex or reported including sex as a covariate in their analysis. It was documented that an article had a purpose of evaluating sex in ATR if it was explicitly stated in their introduction or implied (i.e., ‘personal factors’ and ‘patient characteristics’). Finally, the midpoint of study enrolment was calculated by finding the middle point between the year enrolment began and the year it concluded (i.e., study began enrolment in 2018 and completed in 2020, the midpoint was 2019). Any midpoint of enrolment with a decimal was rounded down (i.e., 2018.5 was changed to 2018). This strategy of using the midpoint of study enrolment was selected over the use of publication year to represent the predictor variable of time, as it more accurately reflects the years in which the injuries occurred.

### Bias assessment

This systematic review focused exclusively on the number of males and females with ATR in research articles rather than on study outcomes. Therefore, the most relevant form of bias to consider was external validity, or how well the participants in the article reflected the population of a region during a specific time. External validity, however, is commonly missing from bias evaluation tools or makes up a small portion of these tools [8]. The Downs and Black [8] checklist is beneficial as it consists of a three‐question subscale specific to external validity, which could be used in isolation. Unfortunately, this subscale had poor performance within inter‐rater reliability, which makes it difficult to use in this review, given the multiple groups of reviewers. Furthermore, over the past few decades, there has been a push for more standardized reporting in research studies. As a result, many older publications do not meet the reporting criteria. Given these limitations, and that the primary purpose of this systematic review was to describe the sex distribution in research articles, a formal bias assessment was not performed.

### Statistical analysis

A plan for statistical analysis was created a priori in collaboration with a biostatistician (R.T.P.) with over 10 years of experience in Achilles tendon research, to ensure an appropriate and robust analytic approach. A weighted linear regression evaluated the relationship between the article midpoint year (of participant enrolment) and the percentage of females across included articles. The percentage of females in each study was weighted by its sample size. This approach allows for a more robust assessment of trends by accounting for variability in study size over time and reducing the influence of smaller sample sizes. Alpha was set to a value of 0.05. Robust standard errors were used as the assumption of data normality was violated and could not be corrected. All statistical testing was performed in IBM SPSS version 29.0.1.0. (IBM Corp). All study data were collected and managed using REDCap electronic data capture tools, including the double data entry tool, hosted at the University of Delaware [10, 11]. All data figures were created using R (version 4.4.1; R Foundation for Statistical Computing).

## RESULTS

### Article identification

**Figure 1:**
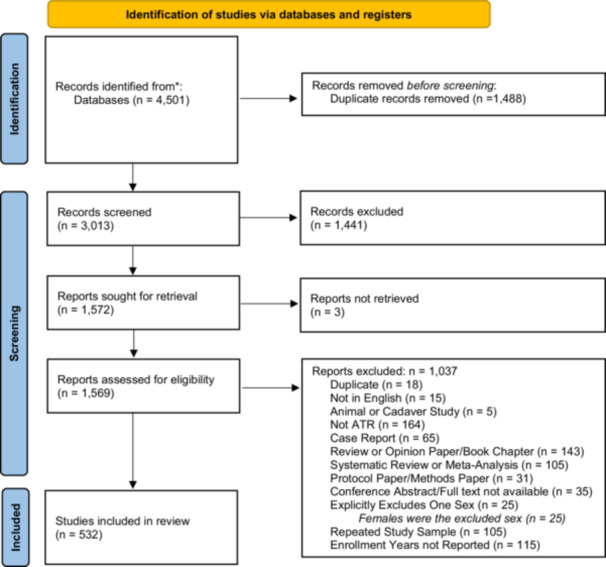
PRISMA flow diagram of included articles. PRISMA, Preferred Reporting Items for Systematic Review and Meta‐Analysis. From Page et al. [26]. ATR, Achilles tendon rupture.

The first search performed on 28 July 2023, yielded 2784 articles (with duplicates removed) and the second search performed on 13 August 2024, yielded 229 (with duplicates removed), which gave a total of 3013 articles. Of the 3013 articles screened, 532 met the inclusion criteria (Figure 1, Table S2). Notably, during the review process, 52 articles were excluded for the implicit or explicit exclusion of one sex (27 during screening, and 25 during full text review). Of the 52 articles, five (9.6%) articles studied females only (inclusion criteria were either female gymnasts or players in the Women's National Basketball Association).

### Article characteristics

Descriptive data of the articles included are shown in Tables 2 and 3. The midpoint year of enrolment ranged from 1948 to 2022, with the median midpoint year of enrolment being 2009 (Table 2). The average percentage of females across all articles was 19 ± 12% (Table 3). The midpoint year with the most articles was 2015, with 34 articles. These 34 articles had a total sample of 105,220 people, with 40,503 being females (38.5%, Table S3). This year also corresponded to the highest percentage of females compared to any other year. Conversely, the lowest percentage of females was 7.4% in 1990 (three publications; *n* = 108), and 8.1% in 1964 (one publication; *n* = 37) (Table S3).

**Table 2:** Descriptive data of the included articles.

Variable	Value
Total articles included (*n*)	532
Midpoint of enrolment in articles (year)	
Min, Max	1948, 2022
Average	2006
Median	2009

**Table 3:** Summary of the included articles' descriptive data.

Variable	Value
Length of enrolment period (years)	6.6 ± 5.1
Sample size (mean)	1066 ± 7773
Sample size (median)	42
Sample size [95% confidence interval]	[404, 1728]
Sample size (min, max)	(4, 112601)
Percentage of males	81 ± 12
Percentage of females	19 ± 12
Participant age (years)	41.6 ± 6.7
Study type, *n* (%)	
Prospective/cross‐sectional	240 (45.1%)
Retrospective	212 (39.8%)
Retrospective + prospective/cross‐sectional follow‐up	80 (15.0%)
Countries contributing the most articles, *n* (%)	
United States	77 (14.5)
United Kingdom	65 (12.2)
China	53 (10.0)
Turkey	33 (6.2)
Sweden	32 (6.0)
Italy	28 (5.3)
Denmark	26 (4.9)

*Note*: Presented as mean ± SD unless otherwise noted.

Abbreviation: SD, standard deviation.

### Study findings on sex distribution

**Figure 2:**
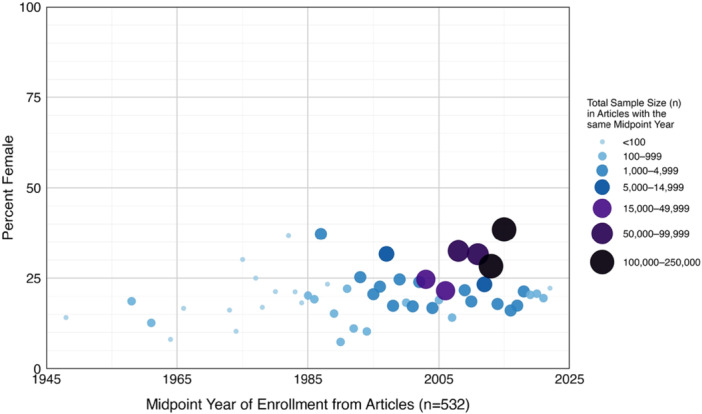
Percentage of females included in ATR publications by midpoint year of enrolment. Articles sharing a midpoint year (e.g., 2016) were grouped, and the total sample size and number of females were summed to calculate the percentage of females for that year. Circle size and colour represent the total sample size for each year. ATR, Achilles tendon rupture.

The midpoint year (of study enrolment) was significantly related to the percentage of females in ATR studies (*b* = 0.386, *p* < 0.001, *R* ^2^ = 0.073). These findings are illustrated in Figure 2.

### Sex‐disaggregated data

**Figure 3:**
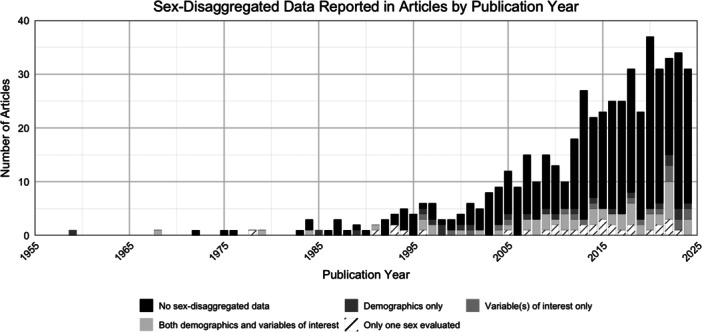
The number of included articles which reported sex‐disaggregated data, broken down by category, and the number that did not provide sex‐disaggregated data, all grouped by publication year. In single‐sex studies, males were the only sex evaluated.

Sex‐disaggregated data were not consistently provided in articles. For example, of the 532 articles included, 57 (10.7%) provided sex‐disaggregated data for both demographics/descriptives and variable(s) of interest, while 4.3% provided sex‐disaggregated data for demographics only, and 3.8% provided sex‐disaggregated data for variable(s) of interest only (Table 4). All descriptive data relating to the inclusion of sex disaggregated data is summarized in Table 4 and illustrated in Figure 3.

**Table 4:** Summary of articles' inclusion of sex disaggregated data.

Descriptor	*n* (%)
No sex disaggregated data	400 (75.2)
Sex considered analytically without the inclusion of sex‐disaggregated data	34/400 (8.5)
Sex disaggregated provided for Demographics ONLY	23 (4.3)
Sex disaggregated provided for variable(s) of interest ONLY	20 (3.8)
Sex disaggregated provided for BOTH demographics and variable(s) of interest	57 (10.7)
Sex disaggregated data provided in supplemental files	6 (1.1)
Only 1 sex was included/evaluated	64 (12.0)
Articles evaluating only males	64 (100)
Articles evaluating only females	0
Purpose of article was to evaluate sex in ATR	12 (2.3)

Abbreviation: ATR, Achilles tendon rupture.

## DISCUSSION

This systematic review represents the largest study to systematically evaluate temporal trends in the sex distribution across the ATR literature, evaluating 532 total articles. It was found that the percentage of females in ATR studies has had a modest change (approximately 0.4% per year) since 1948. The midpoint of study enrolment was used as the temporal measurement, as it more closely reflects when the injuries occurred compared to publication year, which can occur years after study completion. Another notable finding from this systematic review is that of the 532 articles included, 10.7% provided sex‐disaggregated data for both demographics and variable(s) of interest.

The results indicate a modest rise in the number of females with ATR represented in research articles from 1948 to 2022. This change is greater than the findings from Ho et al. [12], who reported an increase of 0.6% every 5 years from 1970 to 2015 (year of article publication) [12]. This discrepancy may be explained by the difference in included articles, with the current systematic review including 532 articles compared to the 120 articles included in the previous review [12]. Additionally, the exclusion of large datasets or databases in the previous review [12] likely resulted in less female representation.

The current findings may be interpreted alongside prior literature documenting a rise in ATR incidence rates [9, 14, 16, 17, 18, 22, 23]. Specifically, there is evidence for greater (relative) increases among females [14, 20]. These observed patterns have been attributed to a more active ageing population [14, 17, 18]. Unfortunately, many studies do not explicitly compare sex‐specific or age‐by‐sex incidence rates with formal statistical analyses [14, 16, 17, 20]. Further, the current systematic review is unable to determine if the change in percentage of females is concentrated in sport‐related activity or within a specific age group, as too few studies provided sex‐disaggregated data. If tied to sports participation, it is unknown if the trend is widespread across sports (i.e., basketball and volleyball), or if it is driven by the unique demands of a single sport (i.e., gymnastics) [3, 6, 15]. Future studies should evaluate if these trends are more commonly observed within sports or within specific age groups to determine if specific groups of females are at a higher risk of ATR now compared to historical trends.

Despite a steady rise in the percentage of females in the ATR literature, the current systematic review highlights a substantial lack of sex‐disaggregated data and very few studies explicitly investigating the role of sex. Although the sex distribution of study samples aligns with known epidemiological patterns [5, 14, 16, 18, 20, 23], data are commonly pooled and analyzed across sexes, which may obscure underlying sex‐specific patterns [1, 4]. The Simpson's paradox, a statistical phenomenon, illustrates how this practice among imbalanced group sizes (i.e., sex) can mask patterns and may even produce misleading results compared to the disaggregated data [1, 4]. While females have historically been affected by this practice [2], pooled analyses may obscure or misrepresent patterns for both sexes [1, 4, 19, 27, 28]. While it may not always be possible to perform sex‐specific analyses (i.e., too few females enroled) [25, 27], reporting data disaggregated by sex in manuscript tables (in text or in the supplemental materials) may be a feasible starting point to unveil sex‐specific trends [24], given ATR incidence is generally greater among males [5, 18]. A greater number of studies with sex‐disaggregated data could allow for trends to be assessed across articles. Ultimately, the inclusion of sex‐disaggregated data can lead to meaningful improvements in the current understanding of ATR in both males and females [2, 27].

## LIMITATIONS

There are limitations to this systematic review that should be considered in the interpretation of the results. This study sought to evaluate the change in the sex distribution across the ATR literature over time. Although the midpoint year was associated with the percentage of females, the proportion of variance was small, indicating that temporal trends account for a limited portion of the variability in the percentage of females in studies over time. This suggests additional factors likely contribute to the increase percentage of females, such as changes in sports participation among females or study recruitment strategies. For example, there was a push to increase female inclusion in research studies by the National Institutes of Health and other funding agencies beginning in the 1990s [21, 32]. However, the current systematic review was not designed to identify underlying factors driving these changes. Therefore, the current findings should be interpreted alongside epidemiological studies which have investigated ATR incidence within specific regions and time periods. Additionally, the current systematic review was unable to evaluate sex‐specific patterns in the literature due to the limited availability of sex‐disaggregated data. Finally, articles were classified as providing sex‐disaggregated data if at least one variable was reported, rather than requiring disaggregation across all reported variables. As such, the estimates presented may overstate the extent to which sex‐disaggregated data are available in the literature.

### Clinical Implications

There has been a modest rise in the percentage of females in the ATR literature since the late 1940s. Despite this increase, sex‐disaggregated data remain scarce, limiting the current understanding of the role of sex in ATR (i.e., presentation and recovery). This systematic review also highlights the importance of improving the inclusion of sex‐disaggregated data in future studies, and its potential benefits for both males and females with ATR. Additionally, future research should investigate whether the observed change in sex distribution reflects a genuine epidemiologic shift and if this pattern is observed within specific subgroups of females to better inform future research needs and clinical care.

## CONCLUSION

This systematic review revealed temporal patterns in the sex distribution in ATR literature. Specifically, it was found that the percentage of females in ATR literature has had a modest increase over the past 74 years. Despite the increase in females, males make up the majority of the studied samples, which aligns with established epidemiological patterns. The paucity of sex‐disaggregated data found in this systematic review limited the evaluation of trends, particularly among females, and highlights that there may be a limited understanding of sex‐specific patterns in ATR.

## AUTHOR CONTRIBUTIONS

Morgan N. Potter and Karin Grävare Silbernagel were responsible for the conceptualization of the study. All authors were involved in study design/methodology. Morgan N. Potter, Jenna Mlecko, Marianne Christensen, Susanna Aufwerber, Sarah E. Katz and Karin Grävare Silbernagel were involved in data collection/data curation. Formal analysis and data visualization was performed by Morgan N. Potter, Ryan T. Pohlig and Karin Grävare Silbernagel. All authors were involved in writing, reviewing, and editing the manuscript. All authors have read and agreed to the published version of the manuscript. Ryan T. Pohlig and Karin Grävare Silbernagel provided supervision to Morgan N. Potter for the study. Karin Grävare Silbernagel is the guarantor of this work.

## CONFLICT OF INTEREST STATEMENT

The authors declare no conflicts of interest.

## ETHICS STATEMENT

The authors have nothing to report.

## Supporting information

Supporting File 1.

Supporting File 2.

Supporting File 3.

## Data Availability

Data are available upon reasonable request by contacting the corresponding author via email. In the email, please outline the specific research questions and research plan, the data needed, and why the data are needed.
