# Supplementary material for: Changes in sex distribution in Achilles tendon rupture literature over 74 years: A systematic review
Source: Knee Surg Sports Traumatol Arthrosc. 2026 Jul 6;34(8):2978–86. doi: 10.1002/ksa.70520 (PMC13359244; doi:10.1002/ksa.70520)
Supplement: Supplementary file 1 — Supporting File 1. [file KSA-34-2978-s002.docx]

Changes in Sex Distribution in Achilles Tendon Rupture Literature Over 74 Years: A Systematic Review

KSSTA

Authors: Potter MN, Mlecko J, Christensen M, Aufwerber S, Katz SE, Pohlig RT, Silbernagel SG.

**Supplemental Table 1**. Search strategy used for each database.

| **Database** | **Search Terms** |
| --- | --- |
| PubMed | ("Achilles Tendon"[Mesh] AND "Rupture"[Mesh]) OR Acute achilles rupture[Title/Abstract] OR acute achilles tendon rupture[Title/Abstract] OR achilles tendon rupture[Title/Abstract] OR achilles tendon ruptures[Title/Abstract] OR achilles tendon repair[Title/Abstract] OR achilles tendon repairs[Title/Abstract] OR achilles ruptures[Title/Abstract] OR acute achilles ruptures[Title/Abstract] OR Calcaneal Tendon rupture[Title/Abstract] OR Calcaneal Tendon ruptures[Title/Abstract] OR (Calcaneal Tendon[Title/Abstract] AND rupture[Title/Abstract]) OR (Tendo Calcaneus[Title/Abstract] AND rupture[Title/Abstract]) OR (Tendo Calcaneus[Title/Abstract] AND ruptures[Title/Abstract]) NOT animal* NOT animals NOT cell* NOT cells NOT cadaver* NOT cadavers NOT rat NOT rats NOT dog NOT dogs NOT rabbit* NOT rabbits NOT bovine* NOT sheep* NOT cattle* NOT pig NOT pigs NOT swine* NOT cat NOT cats NOT mouse NOT mice  Limits:  Search 1: 01/01/0000-07/31/2023 AND Language (English)  Search 2: 07/01/2023-08/01/2024 AND Language (English) |
| Cochrane Central Register of Controlled Trials (CENTRAL) | ID Search Hits  #1 MeSH descriptor: [Achilles Tendon] explode all trees 437  #2 MeSH descriptor: [Rupture] explode all trees 1689  #3 #1 AND #2 132  #4 (Acute achilles rupture OR acute achilles tendon rupture OR achilles tendon rupture OR achilles tendon ruptures OR achilles tendon repair OR achilles tendon repairs OR achilles ruptures OR acute achilles ruptures OR Calcaneal Tendon rupture OR Calcaneal Tendon ruptures OR (Calcaneal Tendon AND rupture) OR (Tendo Calcaneus AND rupture)):ti,ab,kw 415  #5 #3 OR #4 416  #6 (animal*):ti,ab,kw 38260  #7 (cell*):ti,ab,kw 172189  #8 (cadaver*):ti,ab,kw 2775  #9 (rat*):ti,ab,kw 656847  #10 (dog*):ti,ab,kw 2838  #11 (rabbit*):ti,ab,kw 1845  #12 (bovine*):ti,ab,kw 3041  #13 (sheep*):ti,ab,kw 574  #14 (cattle*):ti,ab,kw 2188  #15 (pig*):ti,ab,kw 6951  #16 (swine*):ti,ab,kw 1134  #17 (cat*):ti,ab,kw 109439  #18 (mouse):ti,ab,kw 5794  #19 (mice):ti,ab,kw 5794  #20 #6 OR #7 OR #8 OR #9 OR #10 OR #11 OR #12 OR #13 OR  #14 OR #15 OR #16 OR #17 OR #18 OR #19 835131  #21 #5 NOT #20 269  Search 1: Date (inception – 7/31/2024) AND Language (English)  Search 2: Date (7/1/2023-8/30/2024) AND Language (English) |
| SPORTDiscus with Full Text | AB ( (DE "ACHILLES tendon rupture") OR (Acute achilles rupture OR acute achilles tendon rupture OR achilles tendon rupture OR achilles tendon ruptures OR achilles tendon repair OR achilles tendon repairs OR achilles ruptures OR acute achilles ruptures OR Calcaneal Tendon rupture OR Calcaneal Tendon ruptures OR (Calcaneal Tendon AND rupture) OR (Tendo Calcaneus AND rupture)) ) NOT ( (animal* NOT cell* NOT cadaver* NOT rat* NOT dog* NOT rabbit* NOT bovine* NOT sheep* NOT cattle* NOT pig* NOT swine* NOT cat* NOT cats NOT mouse NOT mice ) )  Limits:  Search 1: Date (inception – July 2023) AND Language (English) Search 2: Date (July 2023 – August 2024) AND Language (English) |
| Web of Science  (All Databases) | (((TS=(“Acute achilles rupture” OR “acute achilles tendon rupture” OR “achilles tendon rupture” OR “achilles tendon ruptures” OR “achilles tendon repair” OR “achilles tendon repairs” OR “achilles ruptures” OR “acute achilles ruptures” OR “Calcaneal Tendon rupture” OR “Calcaneal Tendon ruptures” OR (“Calcaneal Tendon” AND rupture) OR (“Tendo Calcaneus” AND rupture))) NOT ALL=(animal*)) NOT ALL=(cell* )) NOT ALL=(cadaver*) NOT ALL=(rat*) NOT ALL=(dog*) NOT ALL=(rabbit*) NOT ALL=(bovine) NOT ALL=(sheep*) NOT ALL=(cattle) NOT ALL=(pig*) NOT ALL=(swine*) NOT ALL=(cat*) NOT ALL=(mouse) NOT ALL=(mice)  Limits:  Search 1: Publication Date: 1900-2023 AND Language: English AND Article (Document Types)  Search 2: Publication Date: 2023/07/01-2024/08/31 AND Language: English AND Article (Document Types) |
