# Supplementary material for: Changes in sex distribution in Achilles tendon rupture literature over 74 years: A systematic review
Source: Knee Surg Sports Traumatol Arthrosc. 2026 Jul 6;34(8):2978–86. doi: 10.1002/ksa.70520 (PMC13359244; doi:10.1002/ksa.70520)
Supplement: Supplementary file 2 — Supporting File 2. [file KSA-34-2978-s003.docx]

Changes in Sex Distribution in Achilles Tendon Rupture Literature Over 74 Years: A Systematic Review

KSSTA

Authors: Potter MN, Mlecko J, Christensen M, Aufwerber S, Katz SE, Pohlig RT, Silbernagel SG.

**Supplemental Table 2**. Included articles (n=532), organized by publication year, midpoint of enrollment year, and then by first author last name

| Publication Year | Author | Title | Journal/Publication Title | Enrollment Duration (yrs) | Midpoint of Enrollment | Total Sample Size | % Female |
| --- | --- | --- | --- | --- | --- | --- | --- |
| 1959 | ARNER, O.; LINDHOLM, A. | Subcutaneous rupture of the Achilles tendon; a study of 92 cases. | Acta chirurgica Scandinavica. Supplementum | 18 | 1948 | 92 | 14.1 |
| 1968 | Lea, R. B.; Smith, L. | Rupture of the achilles tendon. Nonsurgical treatment. | Clinical orthopaedics and related research | 3 | 1966 | 6 | 16.7 |
| 1972 | Kristensen, J. K.; Andersen, P. T. | Rupture of the Achilles tendon: a series and a review of literature. | The Journal of trauma | 9 | 1964 | 37 | 8.1 |
| 1975 | JESSING, P; HANSEN, E | SURGICAL TREATMENT OF 102 TENDO ACHILLES RUPTURES - SUTURE OR TENONTOPLASTY | ACTA CHIRURGICA SCANDINAVICA | 29 | 1958 | 102 | 18.6 |
| 1976 | Nillius, S. A.; Nilsson, B. E.; Westlin, N. E. | The incidence of Achilles tendon rupture. | Acta orthopaedica Scandinavica | 24 | 1961 | 229 | 12.7 |
| 1978 | Sjöström, M.; Fugl-Meyer, A. R.; Wählby, L. | Achilles tendon injury. Plantar flexion strength and structure of the soleus muscle after surgical repair. | Acta chirurgica Scandinavica | 3 | 1974 | 9 | 0.0 |
| 1979 | Persson, A.; Wredmark, T. | The treatment of total ruptures of the Achilles tendon by plaster immobilisation. | International orthopaedics | 4 | 1974 | 20 | 15.0 |
| 1983 | Marti, R. K.; van der Werken, C.; Schütte, P. R.; Bast, T. J. | Operative repair of ruptured achilles tendon and functional after-treatment--I. Acute rupture. | The Netherlands journal of surgery | 4 | 1978 | 17 | 35.3 |
| 1984 | Hosey, T.; Wertheimer, S. | A retrospective study on surgical repair of the Achilles tendon. | The Journal of foot surgery | 11 | 1977 | 8 | 25.0 |
| 1984 | Keller, J.; Rasmussen, T. B. | Closed treatment of Achilles tendon rupture. | Acta orthopaedica Scandinavica | 5 | 1980 | 37 | 27.0 |
| 1984 | Parsons, J. R.; Rosario, A.; Weiss, A. B.; Alexander, H. | Achilles tendon repair with an absorbable polymer-carbon fiber composite. | Foot & ankle | 3 | 1982 | 14 | 28.6 |
| 1985 | Kellam, J. F.; Hunter, G. A.; McElwain, J. P. | Review of the operative treatment of Achilles tendon rupture. | Clinical orthopaedics and related research | 11 | 1973 | 68 | 16.2 |
| 1986 | Andersen, E.; Hvass, I. | Suture of achilles tendon rupture under local anesthesia. | Acta orthopaedica Scandinavica | 2 | 1983 | 30 | 23.3 |
| 1987 | Carden, D. G.; Noble, J.; Chalmers, J.; Lunn, P.; Ellis, J. | Rupture of the calcaneal tendon. The early and late management. | The Journal of bone and joint surgery. British volume | 13 | 1975 | 73 | 30.1 |
| 1987 | Beskin, J. L.; Sanders, R. A.; Hunter, S. C.; Hughston, J. C. | Surgical repair of Achilles tendon ruptures. | The American journal of sports medicine | 12 | 1978 | 42 | 9.5 |
| 1987 | Crolla, R. M.; van Leeuwen, D. M.; van Ramshorst, B.; van der Werken, C. | Acute rupture of the tendo calcaneus. Surgical repair with functional aftertreatment. | Acta orthopaedica Belgica | 4 | 1983 | 28 | 21.4 |
| 1988 | Lieberman, J. R.; Lozman, J.; Czajka, J.; Dougherty, J. | Repair of Achilles tendon ruptures with Dacron vascular graft. | Clinical orthopaedics and related research | 4 | 1983 | 8 | 12.5 |
| 1989 | Aldam, C. H. | Repair of calcaneal tendon ruptures. A safe technique. | The Journal of bone and joint surgery. British volume | 5 | 1982 | 41 | 46.3 |
| 1989 | Kaalund, S.; Lass, P.; Høgsaa, B.; Nøhr, M. | Achilles tendon rupture in badminton. | British journal of sports medicine | 3 | 1985 | 39 | 10.3 |
| 1990 | Sejberg, D.; Hansen, L. B.; Dalsgaard, S. | Achilles tendon ruptures operated on under local anesthesia. Retrospective study of 81 nonhospitalized patients. | Acta orthopaedica Scandinavica | 7 | 1985 | 97 | 21.6 |
| 1991 | Respizzi, S.; Melloni Ribas, M. | Valutazione isocinetica delle ricostruzioni del tendine d'Achille. / Achilles tendon reconstructions. | Journal of Sports Traumatology & Related Research | 8 | 1980 | 10 | 0.0 |
| 1991 | Mann, R. A.; Holmes, G. B. Jr; Seale, K. S.; Collins, D. N. | Chronic rupture of the Achilles tendon: a new technique of repair. | The Journal of bone and joint surgery. American volume | 4 | 1986 | 5 | 40.0 |
| 1992 | Fruensgaard, S.; Helmig, P.; Riis, J.; Stovring, J. O. | Conservative treatment for acute rupture of the Achilles tendon. | International orthopaedics | 5 | 1984 | 66 | 18.2 |
| 1992 | Carter, T. R.; Fowler, P. J.; Blokker, C. | Functional postoperative treatment of Achilles tendon repair. | The American journal of sports medicine | 3 | 1985 | 21 | 33.3 |
| 1992 | WAGDYMAHMOUD, S; MEGAHED, AH; ELSHESHTAWY, OE | REPAIR OF THE CALCANEAL TENDON - AN IMPROVED TECHNIQUE | JOURNAL OF BONE AND JOINT SURGERY-BRITISH VOLUME | 7 | 1986 | 5 | 20.0 |
| 1993 | FitzGibbons, R. E.; Hefferon, J.; Hill, J. | Percutaneous Achilles tendon repair. | The American journal of sports medicine | 8 | 1985 | 14 | 0.0 |
| 1993 | Rantanen, J.; Hurme, T.; Paananen, M. | Immobilization in neutral versus equinus position after Achilles tendon repair. A review of 32 patients. | Acta orthopaedica Scandinavica | 12 | 1985 | 39 | 20.5 |
| 1993 | Steele, G.J.; Harter, R.A.; Ting, A.J. | Comparison of functional ability following percutaneous and open surgical repairs of acutely ruptured achilles tendons. | Journal of Sport Rehabilitation | 4 | 1987 | 20 | 0.0 |
| 1993 | Saw, Y.; Baltzopoulos, V.; Lim, A.; Rostron, P. K.; Bolton-Maggs, B. G.; Calver, R. F. | Early mobilization after operative repair of ruptured Achilles tendon. | Injury | 2 | 1991 | 19 | 15.8 |
| 1994 | Cetti, R.; Henriksen, L. O.; Jacobsen, K. S. | A new treatment of ruptured Achilles tendons. A prospective randomized study. | Clinical orthopaedics and related research | 2 | 1985 | 60 | 16.7 |
| 1994 | Massari, L.; Cinotti, A.; Mannella, P.; Traina, G. C. | Clinical and ultrasound follow-up of 62 patients submitted to the surgical treatment of subcutaneous rupture of the Achilles tendon. | La Chirurgia degli organi di movimento | 7 | 1986 | 65 | 12.3 |
| 1994 | Giannini, S.; Girolami, M.; Ceccarelli, F.; Catani, F.; Stea, S. | Surgical repair of achilles tendon ruptures using polypropylene braid augmentation. | Foot & ankle international | 3 | 1988 | 15 | 20.0 |
| 1994 | Sölveborn, S. A.; Moberg, A. | Immediate free ankle motion after surgical repair of acute Achilles tendon ruptures. | The American journal of sports medicine | 2 | 1989 | 17 | 11.8 |
| 1994 | Kosanović, M.; Cretnik, A.; Batista, M. | Subcutaneous suturing of the ruptured Achilles tendon under local anaesthesia. | Archives of orthopaedic and trauma surgery | 2 | 1991 | 35 | 0.0 |
| 1995 | Boyden, E. M.; Kitaoka, H. B.; Cahalan, T. D.; An, K. N. | Late versus early repair of Achilles tendon rupture. Clinical and biomechanical evaluation. | Clinical orthopaedics and related research | 13 | 1982 | 21 | 23.8 |
| 1995 | Kakiuchi, M. | A combined open and percutaneous technique for repair of tendo Achillis. Comparison with open repair. | The Journal of bone and joint surgery. British volume | 4 | 1986 | 22 | 13.6 |
| 1995 | Troop, R. L.; Losse, G. M.; Lane, J. G.; Robertson, D. B.; Hastings, P. S.; Howard, M. E. | Early motion after repair of Achilles tendon ruptures. | Foot & ankle international | 4 | 1989 | 13 | 15.4 |
| 1995 | Gallant, G. G.; Massie, C.; Turco, V. J. | Assessment of eversion and plantar flexion strength after repair of Achilles tendon rupture using peroneus brevis tendon transfer. | American journal of orthopedics (Belle Mead, N.J.) | 5 | 1990 | 8 | 12.5 |
| 1996 | Leppilahti, J.; Puranen, J.; Orava, S. | ABO blood group and Achilles tendon rupture. | Annales chirurgiae et gynaecologiae | 16 | 1986 | 215 | 16.7 |
| 1996 | Leppilahti, J.; Puranen, J.; Orava, S. | Incidence of Achilles tendon rupture. | Acta orthopaedica Scandinavica | 16 | 1986 | 110 | 15.5 |
| 1996 | Efstathopoulos, N.; Agoropoulos, Z.; Papachristou, G.; Karachalios, G. G.; Kokorogiannis, K.; Kaloudis, J. | The modified Bosworth technique for the treatment of acute traumatic Achilles tendon rupture. | European journal of orthopaedic surgery & traumatology : orthopedie traumatologie | 12 | 1988 | 15 | 26.7 |
| 1996 | Leppilahti, J.; Sira, P.; Vanharanta, H.; Orava, S. | Isokinetic evaluation of calf muscle performance after Achilles rupture repair. / Evaluation isocinetique de la performance des muscles du mollet apres une reparation de rupture du tendon d ' Achille. | International Journal of Sports Medicine | 6 | 1989 | 101 | 14.9 |
| 1996 | Möller, A.; Astron, M.; Westlin, N. | Increasing incidence of Achilles tendon rupture. | Acta orthopaedica Scandinavica | 5 | 1989 | 153 | 13.7 |
| 1996 | Karjalainen, P. T.; Ahovuo, J.; Pihlajamäki, H. K.; Soila, K.; Aronen, H. J. | Postoperative MR imaging and ultrasonography of surgically repaired Achilles tendon ruptures. | Acta radiologica (Stockholm, Sweden : 1987) | 3 | 1992 | 13 | 0.0 |
| 1997 | Levi, N. | The incidence of Achilles tendon rupture in Copenhagen. | Injury | 18 | 1986 | 209 | 25.8 |
| 1997 | Buchgraber, A.; Pässler, H. H. | Percutaneous repair of Achilles tendon rupture. Immobilization versus functional postoperative treatment. | Clinical orthopaedics and related research | 6 | 1989 | 48 | 20.8 |
| 1997 | Porter, D. A.; Mannarino, F. P.; Snead, D.; Gabel, S. J.; Ostrowski, M. | Primary repair without augmentation for early neglected Achilles tendon ruptures in the recreational athlete. | Foot & ankle international | 8 | 1990 | 11 | 9.1 |
| 1997 | McComis, G. P.; Nawoczenski, D. A.; DeHaven, K. E. | Functional bracing for rupture of the Achilles tendon. Clinical results and analysis of ground-reaction forces and temporal data. | The Journal of bone and joint surgery. American volume | 4 | 1991 | 15 | 13.3 |
| 1997 | Motta, P.; Errichiello, C.; Pontini, I. | Achilles tendon rupture: a new technique for easy surgical repair and immediate movement of the ankle and foot. | American Journal of Sports Medicine | 6 | 1991 | 71 | 15.5 |
| 1997 | Karjalainen, P. T.; Aronen, H. J.; Pihlajamäki, H. K.; Soila, K.; Paavonen, T.; Böstman, O. M. | Magnetic resonance imaging during healing of surgically repaired Achilles tendon ruptures. | The American journal of sports medicine | 1 | 1994 | 20 | 20.0 |
| 1998 | Maffulli, N. | The clinical diagnosis of subcutaneous tear of the Achilles tendon. A prospective study in 174 patients. | The American journal of sports medicine | 14 | 1989 | 174 | 14.4 |
| 1998 | Fahlström, M.; Björnstig, U.; Lorentzon, R. | Acute Achilles tendon rupture in badminton players. | The American journal of sports medicine | 5 | 1992 | 31 | 12.9 |
| 1998 | Aoki, M.; Ogiwara, N.; Ohta, T.; Nabeta, Y. | Early active motion and weightbearing after cross-stitch achilles tendon repair. | The American journal of sports medicine | 2 | 1995 | 22 | 50.0 |
| 1999 | Maffulli, N.; Waterston, S. W.; Squair, J.; Reaper, J.; Douglas, A. S. | Changing incidence of Achilles tendon rupture in Scotland: a 15-year study. | Clinical journal of sport medicine : official journal of the Canadian Academy of Sport Medicine | 16 | 1987 | 4201 | 37.4 |
| 1999 | Mortensen, H. M.; Skov, O.; Jensen, P. E. | Early motion of the ankle after operative treatment of a rupture of the Achilles tendon. A prospective, randomized clinical and radiographic study. | The Journal of bone and joint surgery. American volume | 2 | 1991 | 71 | 28.2 |
| 1999 | Webb, J. M.; Bannister, G. C. | Percutaneous repair of the ruptured tendo Achillis. | The Journal of bone and joint surgery. British volume | 5 | 1994 | 27 | 22.2 |
| 2000 | Horstmann, T; Lukas, C; Mayer, F; Winter, E; Ambacher, T; Heitkamp, HC; Dickhuth, HH | Isokinetic strength and strength endurance of the lower limb musculature ten years after achilles tendon repair | ISOKINETICS AND EXERCISE SCIENCE | 12 | 1985 | 63 | 23.8 |
| 2000 | Leppilahti, J.; Lähde, S.; Forsman, K.; Kangas, J.; Kauranen, K.; Orava, S. | Relationship between calf muscle size and strength after achilles rupture repair. | Foot & ankle international | 6 | 1989 | 85 | 14.1 |
| 2000 | Nestorson, J.; Movin, T.; Möller, M.; Karlsson, J. | Function after Achilles tendon rupture in the elderly: 25 patients older than 65 years followed for 3 years. | Acta orthopaedica Scandinavica | 6 | 1994 | 25 | 16.0 |
| 2000 | Maffulli, N.; Barrass, V.; Ewen, S.W.B. | Light microscopic histology of Achilles tendon ruptures: a comparison with unruptured tendons. / Histologie legere microscopique des ruptures du tendon d ' Achille: comparaison avec les tendons sains. | American Journal of Sports Medicine | 2 | 1997 | 38 | 28.9 |
| 2001 | Roberts, C. P.; Palmer, S.; Vince, A.; Deliss, L. J. | Dynamised cast management of Achilles tendon ruptures. | Injury | 14 | 1991 | 49 | 46.9 |
| 2001 | Jaakkola, JI; Beskin, JL; Griffith, LH; Cernansky, G | Early ankle motion after triple bundle technique repair vs. casting for acute Achilles tendon rupture | FOOT & ANKLE INTERNATIONAL | 15 | 1992 | 73 | 12.3 |
| 2001 | Pintore, E.; Barra, V.; Pintore, R.; Maffulli, N. | Peroneus brevis tendon transfer in neglected tears of the Achilles tendon. | The Journal of trauma | 7 | 1993 | 59 | 8.5 |
| 2001 | Maffulli, N.; Thorpe, A. P.; Smith, E. W. | Magnetic resonance imaging after operative repair of achilles tendon rupture. | Scandinavian journal of medicine & science in sports | 1 | 1996 | 16 | 25.0 |
| 2001 | Möller, M.; Movin, T.; Granhed, H.; Lind, K.; Faxén, E.; Karlsson, J. | Acute rupture of tendon Achillis. A prospective randomised study of comparison between surgical and non-surgical treatment. | The Journal of bone and joint surgery. British volume | 3 | 1996 | 112 | 11.6 |
| 2002 | Kerkhoffs, GMMJ; Struijs, PAA; Raaymakers, ELFB; Marti, RK | Functional treatment after surgical repair of acute Achilles tendon rupture: wrap vs walking cast | ARCHIVES OF ORTHOPAEDIC AND TRAUMA SURGERY | 4 | 1991 | 39 | 17.9 |
| 2002 | Coutts, A.; MacGregor, A.; Gibson, J.; Maffulli, N. | Clinical and functional results of open operative repair for Achilles tendon rupture in a non-specialist surgical unit. | Journal of the Royal College of Surgeons of Edinburgh | 10 | 1994 | 25 | 48.0 |
| 2002 | Kauranen, Kari; Kangas, Jarmo; Leppilahti, Juhana | Recovering motor performance of the foot after Achilles rupture repair: a randomized clinical study about early functional treatment vs. early immobilization of Achilles tendon in tension. | Foot & ankle international | 2 | 1995 | 30 | 13.3 |
| 2002 | Möller, Michael; Kälebo, Peter; Tidebrant, Göran; Movin, Tomas; Karlsson, Jon | The ultrasonographic appearance of the ruptured Achilles tendon during healing: a longitudinal evaluation of surgical and nonsurgical treatment, with comparisons to MRI appearance. | Knee surgery, sports traumatology, arthroscopy : official journal of the ESSKA | 3 | 1996 | 65 | 15.4 |
| 2002 | Follak, Niels; Ganzer, Dirk; Merk, Harry | The utility of gait analysis in the rehabilitation of patients after surgical treatment of Achilles tendon rupture. | European journal of orthopaedic surgery & traumatology : orthopedie traumatologie | 4 | 1997 | 30 | 20.0 |
| 2002 | Magnusson, S. P.; Qvortrup, K.; Larsen, J. O.; Rosager, S.; Hanson, P.; Aagaard, P.; Krogsgaard, M.; Kjaer, M. | Collagen fibril size and crimp morphology in ruptured and intact Achilles tendons. | Matrix biology : journal of the International Society for Matrix Biology | 2 | 2000 | 10 | 40.0 |
| 2003 | van der Linden, Paul D.; Sturkenboom, Miriam C. J. M.; Herings, Ron M. C.; Leufkens, Hubert M. G.; Rowlands, Sam; Stricker, Bruno H. Ch | Increased risk of achilles tendon rupture with quinolone antibacterial use, especially in elderly patients taking oral corticosteroids. | Archives of internal medicine | 11 | 1993 | 1367 | 30.6 |
| 2003 | Josey, Robert A.; Marymont, John V.; Varner, Kevin E.; Borom, Andy; O'Connor, Dan; Oates, Jay C. | Immediate, full weightbearing cast treatment of acute Achilles tendon ruptures: a long-term follow-up study. | Foot & ankle international | 10 | 1994 | 39 | 10.3 |
| 2003 | Weber, Martin; Niemann, Marco; Lanz, Renate; Müller, Thorsten | Nonoperative treatment of acute rupture of the achilles tendon: results of a new protocol and comparison with operative treatment. | The American journal of sports medicine | 6 | 1995 | 40 | 30.0 |
| 2003 | Halasi, Tamás; Tállay, András; Berkes, István | Percutaneous Achilles tendon repair with and without endoscopic control. | Knee surgery, sports traumatology, arthroscopy : official journal of the ESSKA | 5 | 1996 | 144 | 14.6 |
| 2003 | Maffulli, N.; Tallon, C.; Wong, J.; Peng Lim, K.; Bleakney, R. | No adverse effect of early weight bearing following open repair of acute tears of the Achilles tendon. | The Journal of sports medicine and physical fitness | 3 | 1997 | 53 | 15.1 |
| 2003 | Maffulli, Nicola; Tallon, Cheryl; Wong, Jason; Lim, Kim Peng; Bleakney, Robert | Early weightbearing and ankle mobilization after open repair of acute midsubstance tears of the achilles tendon. | The American journal of sports medicine | 2 | 1998 | 53 | 15.1 |
| 2003 | Costa, M. L.; Shepstone, L.; Darrah, C.; Marshall, T.; Donell, S. T. | Immediate full-weight-bearing mobilisation for repaired Achilles tendon ruptures: a pilot study. | Injury | 2 | 1999 | 28 | 14.3 |
| 2003 | Ozgurtas, Taner; Yildiz, Cemil; Serdar, Muhittin; Atesalp, Sabri; Kutluay, Turker | Is high concentration of serum lipids a risk factor for Achilles tendon rupture? | Clinica chimica acta; international journal of clinical chemistry | 3 | 1999 | 47 | 12.8 |
| 2004 | Bruggeman, Nicholas B.; Turner, Norman S.; Dahm, Diane L.; Voll, Anthony E.; Hoskin, Tanya L.; Jacofsky, David J.; Haidukewych, George J. | Wound complications after open Achilles tendon repair: an analysis of risk factors. | Clinical orthopaedics and related research | 24 | 1989 | 164 | 17.1 |
| 2004 | Arøen, A.; Helgø, D.; Granlund, O. G.; Bahr, R. | Contralateral tendon rupture risk is increased in individuals with a previous Achilles tendon rupture. | Scandinavian journal of medicine & science in sports | 8 | 1993 | 168 | 15.5 |
| 2004 | Cretnik, Andrej; Frank, Aleksander | Incidence and outcome of rupture of the Achilles tendon. | Wiener klinische Wochenschrift | 6 | 1993 | 113 | 5.3 |
| 2004 | Haji, Amyn; Sahai, Arun; Symes, Andrew; Vyas, J. K. | Percutaneous versus open tendo achillis repair. | Foot & ankle international | 18 | 1993 | 108 | 28.7 |
| 2004 | Cretnik, Andrej; Kosanović, Milos; Smrkolj, Vladimir | Percutaneous suturing of the ruptured Achilles tendon under local anesthesia. | The Journal of foot and ankle surgery : official publication of the American College of Foot and Ankle Surgeons | 7 | 1994 | 132 | 6.1 |
| 2004 | Wallace, Richard G. H.; Traynor, Ingrid E. R.; Kernohan, W. George; Eames, Michael H. A. | Combined conservative and orthotic management of acute ruptures of the Achilles tendon. | The Journal of bone and joint surgery. American volume | 7 | 1995 | 140 | 27.9 |
| 2004 | van der Linden-van der Zwaag, Henrica M. J.; Nelissen, Rob G. H. H.; Sintenie, Jan B. | Results of surgical versus non-surgical treatment of Achilles tendon rupture. | International orthopaedics | 12 | 1995 | 292 | 27.1 |
| 2004 | Kraus, Ralf; Stahl, Jens-Peter; Meyer, Christof; Pavlidis, Theodorus; Alt, Volker; Horas, Uwe; Schnettler, Reinhard | Frequency and effects of intratendinous and peritendinous calcifications after open Achilles tendon repair. | Foot & ankle international | 3 | 2000 | 36 | 11.1 |
| 2005 | Rettig, Arthur C.; Liotta, Ferdinand J.; Klootwyk, Thomas E.; Porter, David A.; Mieling, Paul | Potential risk of rerupture in primary achilles tendon repair in athletes younger than 30 years of age. | The American journal of sports medicine | 8 | 1990 | 89 | 6.7 |
| 2005 | Miller, D.; Waterston, S.; Reaper, J.; Barrass, V.; Maffulli, N. | Conservative management, percutaneous or open repair of acute Achilles tendon rupture: a retrospective study. | Scottish medical journal | 7 | 1993 | 172 | 19.8 |
| 2005 | Cretnik, Andrej; Kosanovic, Milos; Smrkolj, Vladimir | Percutaneous versus open repair of the ruptured Achilles tendon: a comparative study. | The American journal of sports medicine | 7 | 1994 | 237 | 5.9 |
| 2005 | Lee, Yih-Shiunn; Lin, Chien-Chung; Chen, Cheng-Nan; Chen, Shih-Hao; Liao, Wen-Yun; Huang, Chien-Rae | Reconstruction for neglected Achilles tendon rupture: the modified Bosworth technique. | Orthopedics | 10 | 1995 | 12 | 25.0 |
| 2005 | Garabito, Agustin; Martinez-Miranda, Jesus; Sanchez-Sotelo, Joaquin | Augmented repair of acute Achilles tendon ruptures using gastrocnemius-soleus fascia. | International orthopaedics | 7 | 1998 | 54 | 11.1 |
| 2005 | Ingvar, Jonas; Tägil, Magnus; Eneroth, Magnus | Nonoperative treatment of Achilles tendon rupture: 196 consecutive patients with a 7% re-rupture rate. | Acta orthopaedica | 5 | 1998 | 196 | 13.3 |
| 2005 | Wagnon, Raymond; Akayi, Mathieu | The Webb-Bannister percutaneous technique for acute Achilles' tendon ruptures: a functional and MRI assessment. | The Journal of foot and ankle surgery : official publication of the American College of Foot and Ankle Surgeons | 9 | 1998 | 57 | 14.0 |
| 2005 | Maffulli, Nicola; Leadbetter, Wayne B. | Free Gracilis Tendon Graft in Neglected Tears of the Achilles Tendon. | Clinical Journal of Sport Medicine | 4 | 1999 | 21 | 23.8 |
| 2005 | Rajasekar, Kumariah; Gholve, Purushottam; Faraj, Adnan A.; Kosygan, Kornad P. | A subjective outcome analysis of tendo-Achilles rupture. | The Journal of foot and ankle surgery : official publication of the American College of Foot and Ankle Surgeons | 5 | 1999 | 35 | 28.6 |
| 2005 | Goren, David; Ayalon, Moshe; Nyska, Meir | Isokinetic strength and endurance after percutaneous and open surgical repair of Achilles tendon ruptures. | Foot & ankle international | 4 | 2000 | 20 | 10.0 |
| 2005 | Kuskucu, Mesih; Mahirogullari, Mahir; Solakoglu, Can; Akmaz, Ibrahim; Rodop, Osman; Kiral, Ahmet; Kaplan, Haluk | Treatment of rupture of the Achilles tendon with fibrin sealant. | Foot & ankle international | 6 | 2000 | 29 | 0.0 |
| 2005 | Suchak, Amar A.; Bostick, Geoff; Reid, David; Blitz, Sandra; Jomha, Nadr | The incidence of Achilles tendon ruptures in Edmonton, Canada. | Foot & ankle international | 5 | 2000 | 391 | 19.9 |
| 2005 | Burssens, Peter; Forsyth, Ramses; Steyaert, Adelheid; Van Ovost, Edwin; Praet, Marleen; Verdonk, René | Influence of burst TENS stimulation on collagen formation after Achilles tendon suture in man. A histological evaluation with Movat's pentachrome stain. | Acta orthopaedica Belgica | 2 | 2002 | 20 | 25.0 |
| 2006 | Hufner, Tobias M.; Brandes, Dirk B.; Thermann, Hajo; Richter, Martinus; Knobloch, Karsten; Krettek, Christian | Long-Term Results After Functional Nonoperative Treatment of Achilles Tendon Rupture. | Foot & Ankle International | 7 | 1993 | 125 | 16.0 |
| 2006 | Maes, Renaud; Copin, Gerard; Averous, Christophe | Is percutaneous repair of the Achilles tendon a safe technique? A study of 124 cases. | Acta orthopaedica Belgica | 6 | 1995 | 124 | 36.3 |
| 2006 | Ng, E. S.; Ng, Y. O.; Gupta, R.; Lim, F.; Mah, E. | Repair of acute Achilles tendon rupture using a double-ended needle. | Journal of orthopaedic surgery (Hong Kong) | 6 | 1997 | 68 | 30.9 |
| 2006 | Majewski, M; Rohrbach, M; Czaja, S; Ochsner, P | Avoiding sural nerve injuries during percutaneous Achilles tendon repair | AMERICAN JOURNAL OF SPORTS MEDICINE | 10 | 1998 | 84 | 16.7 |
| 2006 | Tezeren, Gündüz; Kuru, Ilhami | Augmentation vs Nonaugmentation Techniques for Open Repairs of Achilles Tendon Ruptures with Early Functional Treatment: A Prospective Randomized Study. | Journal of sports science & medicine | 4 | 1998 | 24 | 25.0 |
| 2006 | Seeger, John D.; West, William A.; Fife, Daniel; Noel, Gary J.; Johnson, Larry N.; Walker, Alexander M. | Achilles tendon rupture and its association with fluoroquinolone antibiotics and other potential risk factors in a managed care population. | Pharmacoepidemiology and drug safety | 5 | 1999 | 947 | 26.9 |
| 2006 | Bassi, Jiwan Lal; Mahindra, Pankaj | A modified flap technique as an alternate procedure for open Achilles tendon repair (the Bassi Method). | Operative Orthopadie und Traumatologie | 4 | 2002 | 11 | 36.4 |
| 2006 | Kotnis, Rohit; David, Saronjini; Handley, Robert; Willett, Keith; Ostlere, Simon | Dynamic ultrasound as a selection tool for reducing achilles tendon reruptures. | The American journal of sports medicine | 6 | 2002 | 125 | 29.6 |
| 2007 | Strauss, Eric J.; Ishak, Charbel; Jazrawi, Laith; Sherman, Orrin; Rosen, Jeffrey | Operative treatment of acute Achilles tendon ruptures: an institutional review of clinical outcomes. | Injury | 14 | 1995 | 52 | 15.4 |
| 2007 | Kangas, Jarmo; Pajala, Ari; Ohtonen, Pasi; Leppilahti, Juhana | Achilles tendon elongation after rupture repair: a randomized comparison of 2 postoperative regimens. | The American journal of sports medicine | 4 | 1996 | 50 | 8.0 |
| 2007 | Sode, Jacob; Obel, Niels; Hallas, Jesper; Lassen, Annmarie | Use of fluroquinolone and risk of Achilles tendon rupture: a population-based cohort study. | European journal of clinical pharmacology | 12 | 1996 | 1538 | 25.0 |
| 2007 | Twaddle, Bruce C.; Poon, Peter | Early motion for Achilles tendon ruptures: is surgery important? A randomized, prospective study. | The American journal of sports medicine | 6 | 1999 | 42 | 33.3 |
| 2007 | Sánchez, Mikel; Anitua, Eduardo; Azofra, Juan; Andía, Isabel; Padilla, Sabino; Mujika, Iñigo | Comparison of surgically repaired Achilles tendon tears using platelet-rich fibrin matrices. | The American journal of sports medicine | 8 | 2000 | 12 | 0.0 |
| 2007 | Lansdaal, J. R.; Goslings, J. C.; Reichart, M.; Govaert, G. A. M.; van Scherpenzeel, K. M.; Haverlag, R.; Ponsen, K. J. | The results of 163 Achilles tendon ruptures treated by a minimally invasive surgical technique and functional aftertreatment. | Injury | 7 | 2001 | 163 | 21.5 |
| 2007 | Elias, Ilan; Besser, Marcus; Nazarian, Levon N.; Raikin, Steven M. | Reconstruction for missed or neglected Achilles tendon rupture with V-Y lengthening and flexor hallucis longus tendon transfer through one incision. | Foot & ankle international | 4 | 2002 | 15 | 33.3 |
| 2007 | Fujikawa, Akira; Kyoto, Yukishige; Kawaguchi, Masahisa; Naoi, Yutaka; Ukegawa, Yo | Achilles tendon after percutaneous surgical repair: serial MRI observation of uncomplicated healing. | AJR. American journal of roentgenology | 5 | 2002 | 39 | 7.7 |
| 2007 | Lapidus, Lasse J.; Rosfors, Stefan; Ponzer, Sari; Levander, Catharina; Elvin, Anders; Lärfars, Gerd; de Bri, Edin | Prolonged thromboprophylaxis with dalteparin after surgical treatment of achilles tendon rupture: a randomized, placebo-controlled study. | Journal of orthopaedic trauma | 4 | 2002 | 105 | 21.0 |
| 2007 | Uchiyama, Eiji; Nomura, Akiko; Takeda, Yasushi; Hiranuma, Kenji; Iwaso, Hiroshi | A modified operation for Achilles tendon ruptures. | The American journal of sports medicine | 5 | 2002 | 100 | 30.0 |
| 2007 | Yasuda, Toshito; Kinoshita, Mitsuo; Okuda, Ryuzo | Reconstruction of chronic achilles tendon rupture with the use of interposed tissue between the stumps. | The American journal of sports medicine | 3 | 2002 | 6 | 33.3 |
| 2007 | Blankstein, Alexander; Israeli, Amnon; Dudkiewicz, Israel; Chechik, Aharon; Ganel, Avraham | Percutaneous Achilles tendon repair combined with real-time sonography. | The Israel Medical Association journal : IMAJ | 3 | 2003 | 20 | 15.0 |
| 2007 | Schepull, Thorsten; Kvist, Joanna; Andersson, Christer; Aspenberg, Per | Mechanical properties during healing of Achilles tendon ruptures to predict final outcome: a pilot Roentgen stereophotogrammetric analysis in 10 patients. | BMC musculoskeletal disorders | 1 | 2004 | 10 | 20.0 |
| 2008 | Hohendorff, B; Siepen, W; Spiering, L; Staub, L; Schmuck, T; Boss, A | Long-term results after operatively treated Achilles tendon rupture: Fibrin glue versus suture | JOURNAL OF FOOT & ANKLE SURGERY | 13 | 1993 | 58 | 15.5 |
| 2008 | Kosanović, Miloš; Brilej, Drago | Chronic rupture of Achilles tendon: is the percutaneous suture technique effective? | Archives of Orthopaedic & Trauma Surgery | 15 | 1998 | 22 | 9.1 |
| 2008 | Ateschrang, Atesch; Gratzer, Christoph; Weise, Kuno | Incidence and effect of calcifications after open-augmented Achilles tendon repair. | Archives of orthopaedic and trauma surgery | 6 | 1999 | 163 | 19.0 |
| 2008 | Fortis, Athanasios P.; Dimas, Anastasios; Lamprakis, Andreas A. | Repair of achilles tendon rupture under endoscopic control. | Arthroscopy : the journal of arthroscopic & related surgery : official publication of the Arthroscopy Association of North America and the International Arthroscopy Association | 6 | 2000 | 20 | 10.0 |
| 2008 | Majewski, M; Schaeren, S; Kohlhaas, U; Ochsner, PE | Postoperative rehabilitation after percutaneous Achilles tendon repair: Early functional therapy versus cast immobilization | DISABILITY AND REHABILITATION | 9 | 2001 | 28 | 7.1 |
| 2008 | Ebinesan, Ananthan Dave; Sarai, Bhupinder Singh; Walley, Gayle D.; Maffulli, Nicola | Conservative, open or percutaneous repair for acute rupture of the Achilles tendon. | Disability and rehabilitation | 3 | 2002 | 63 | 31.7 |
| 2008 | Fotiadis, Elias; Chatzisimeon, Apostolos; Samoladas, Efthimios; Antonarakos, Petros; Akritopoulos, Panagiotis; Akritopoulou, Kiriaki | A Combined Repair Technique for Early Neglected Achilles Tendon Ruptures. | European journal of trauma and emergency surgery : official publication of the European Trauma Society | 5 | 2002 | 9 | 11.1 |
| 2008 | Chan, S. K.; Chung, Stephen C. Y.; Ho, Y. F. | Minimally invasive repair of ruptured Achilles tendon. | Hong Kong medical journal = Xianggang yi xue za zhi | 4 | 2003 | 15 | 6.7 |
| 2008 | Hong Geun Jung; Keun Bae Lee; Sang Gwon Cho; Taek Rim Yoon | Outcome of Achilles Tendon Ruptures Treated by a Limited Open Technique. | Foot & Ankle International | 4 | 2004 | 28 | 32.1 |
| 2008 | Metz, Roderick; Verleisdonk, Egbert-Jan M. M.; van der Heijden, Geert J.-M.-G.; Clevers, Geert-Jan; Hammacher, Erik R.; Verhofstad, Michiel H. J.; van der Werken, Christiaan | Acute Achilles tendon rupture: minimally invasive surgery versus nonoperative treatment with immediate full weightbearing--a randomized controlled trial. | The American journal of sports medicine | 2 | 2004 | 83 | 20.5 |
| 2008 | Schönberger, T. J. A.; Janzing, H. M. J.; Morrenhof, J. W.; de Visser, A. C.; Muitjens, P. | Operative treatment of acute Achilles tendon rupture: Open end-to-end-reconstruction versus reconstruction with Mitek-anchors. | Acta chirurgica Belgica | 2 | 2004 | 40 | 22.5 |
| 2008 | Suchak, Amar A.; Bostick, Geoff P.; Beaupré, Lauren A.; Durand, D'Arcy C.; Jomha, Nadr M. | The influence of early weight-bearing compared with non-weight-bearing after surgical repair of the Achilles tendon. | The Journal of bone and joint surgery. American volume | 4 | 2004 | 110 | 15.5 |
| 2009 | Hohendorff, Bernd; Siepen, Wolf; Staub, Lukas | Treatment of acute Achilles tendon rupture: fibrin glue versus fibrin glue augmented with the plantaris longus tendon. | The Journal of foot and ankle surgery : official publication of the American College of Foot and Ankle Surgeons | 13 | 1993 | 31 | 22.6 |
| 2009 | De Carli, A.; Vadalà, A.; Ciardini, R.; Iorio, R.; Ferretti, A. | Spontaneous Achilles tendon ruptures treated with a mini-open technique: clinical and functional evaluation. | The Journal of sports medicine and physical fitness | 7 | 1998 | 20 | 30.0 |
| 2009 | Pajala, Ari; Kangas, Jarmo; Siira, Pertti; Ohtonen, Pasi; Leppilahti, Juhana | Augmented compared with nonaugmented surgical repair of a fresh total Achilles tendon rupture. A prospective randomized study. | The Journal of bone and joint surgery. American volume | 4 | 1999 | 60 | 11.7 |
| 2009 | El Shewy, Mohamed Taha; El Barbary, Hassan Magdy; Abdel-Ghani, Hisham | Repair of chronic rupture of the achilles tendon using 2 intratendinous flaps from the proximal gastrocnemius-soleus complex. | The American journal of sports medicine | 4 | 2000 | 11 | 18.2 |
| 2009 | Doral, Mahmut Nedim; Bozkurt, Murat; Turhan, Egemen; Ayvaz, Mehmet; Atay, Ozgür Ahmet; Uzümcügil, Akin; Leblebicioğlu, Gürsel; Kaya, Defne; Aydoğ, Tolga | Percutaneous suturing of the ruptured Achilles tendon with endoscopic control. | Archives of orthopaedic and trauma surgery | 7 | 2002 | 62 | 6.5 |
| 2009 | Mahajan, Ravindra H.; Dalal, Rakesh B. | Flexor hallucis longus tendon transfer for reconstruction of chronically ruptured Achilles tendons. | Journal of orthopaedic surgery (Hong Kong) | 6 | 2002 | 25 | 28.0 |
| 2009 | Bertelli, R.; Gaiani, L.; Palmonari, M. | Neglected rupture of the Achilles tendon treated with a percutaneous technique. | Foot and ankle surgery : official journal of the European Society of Foot and Ankle Surgeons | 8 | 2003 | 20 | 10.0 |
| 2009 | Dargel, Jens; Ninck, Jutta; Koebke, Jürgen; Appell, Hans-Joachim; Pennig, Dietmar; Hillekamp, Jörn | Influence of knee flexion on plantarflexion moments after open or percutaneous Achilles tendon repair. | Foot & ankle international | 6 | 2003 | 32 | 34.4 |
| 2009 | Goel, Danny P.; Chan, Denise; Watson, Kathryn; Mohtadi, Nicholas | Safety and hospital costs of Achilles tendon surgery: the serendipitous impact of a randomized clinical trial. | Canadian journal of surgery. Journal canadien de chirurgie | 4 | 2003 | 282 | 17.7 |
| 2009 | Ibrahim, Samir Abdul Razik | Surgical treatment of chronic Achilles tendon rupture. | The Journal of foot and ankle surgery : official publication of the American College of Foot and Ankle Surgeons | 4 | 2003 | 14 | 0.0 |
| 2009 | Ozkaya, Ufuk; Parmaksizoglu, Atilla Sancar; Kabukcuoglu, Yavuz; Sokucu, Sami; Basilgan, Seckin | Open minimally invasive Achilles tendon repair with early rehabilitation: functional results of 25 consecutive patients. | Injury | 2 | 2004 | 25 | 24.0 |
| 2009 | Aktas, Seref; Kocaoglu, Baris | Open versus minimal invasive repair with Achillon device. | Foot & ankle international | 4 | 2005 | 40 | 12.5 |
| 2009 | Nilsson-Helander, Katarina; Thurin, Anders; Karlsson, Jon; Eriksson, Bengt I. | High incidence of deep venous thrombosis after Achilles tendon rupture: a prospective study. | Knee surgery, sports traumatology, arthroscopy : official journal of the ESSKA | 4 | 2005 | 95 | 16.8 |
| 2010 | Tenenbaum, Shay; Dreiangel, Niv; Segal, Ayal; Herman, Amir; Israeli, Amnon; Chechik, Ahron | The percutaneous surgical approach for repairing acute Achilles tendon rupture: a comprehensive outcome assessment. | Journal of the American Podiatric Medical Association | 8 | 2000 | 29 | 13.8 |
| 2010 | Neumayer, Felix; Mouhsine, Elyazid; Arlettaz, Yvan; Gremion, Gérald; Wettstein, Michael; Crevoisier, Xavier | A new conservative-dynamic treatment for the acute ruptured Achilles tendon. | Archives of Orthopaedic & Trauma Surgery | 8 | 2001 | 57 | 21.1 |
| 2010 | Tay, Darren; Lin, Heng An; Tan, Benjamin Sa; Chong, Keen Wai; Rikhraj, Inderjeet Singh | Chronic Achilles tendon rupture treated with two turndown flaps and flexor hallucis longus augmentation - two-year clinical outcome. | Annals of the Academy of Medicine, Singapore | 8 | 2001 | 9 | 22.2 |
| 2010 | Maffulli, Nicola; Spiezia, Filippo; Longo, Umile Giuseppe; Denaro, Vincenzo | Less-invasive reconstruction of chronic achilles tendon ruptures using a peroneus brevis tendon transfer. | The American journal of sports medicine | 3 | 2002 | 32 | 12.5 |
| 2010 | Solakoğlu, Can; Mahiroğulları, Mahir; Cakmak, Selami; Tamam, Cüneyt; Kuşkucu, Mesih | Fibrin sealant in the treatment of acute ruptures of the Achilles tendon: long-term results. | Eklem hastaliklari ve cerrahisi = Joint diseases & related surgery | 10 | 2002 | 42 | 0.0 |
| 2010 | Willits, Kevin; Amendola, Annunziato; Bryant, Dianne; Mohtadi, Nicholas G.; Giffin, J. Robert; Fowler, Peter; Kean, Crystal O.; Kirkley, Alexandra | Operative versus Nonoperative Treatment of Acute Achilles Tendon Ruptures: A Multicenter Randomized Trial Using Accelerated Functional Rehabilitation. | Journal of Bone & Joint Surgery, American Volume | 6 | 2002 | 144 | 18.1 |
| 2010 | Quagliarella, Livio; Sasanelli, Nicola; Belgiovine, Giuseppe; Moretti, Lorenzo; Moretti, Biagio | Evaluation of standing vertical jump by ankles acceleration measurement. | Journal of strength and conditioning research | 5 | 2003 | 25 | 0.0 |
| 2010 | Mukundan, C.; El Husseiny, M.; Rayan, F.; Salim, J.; Budgen, A. | "Mini-open" repair of acute tendo Achilles ruptures--the solution? | Foot and ankle surgery : official journal of the European Society of Foot and Ankle Surgeons | 4 | 2005 | 21 | 61.9 |
| 2010 | Nilsson-Helander, Katarina; Silbernagel, Karin Grävare; Thomeé, Roland; Faxén, Eva; Olsson, Nicklas; Eriksson, Bengt I.; Karlsson, Jon | Acute achilles tendon rupture: a randomized, controlled study comparing surgical and nonsurgical treatments using validated outcome measures. | The American journal of sports medicine | 4 | 2005 | 97 | 18.6 |
| 2010 | Yotsumoto, Tadahiko; Miyamoto, Wataru; Uchio, Yuji | Novel approach to repair of acute achilles tendon rupture: early recovery without postoperative fixation or orthosis. | The American journal of sports medicine | 3 | 2005 | 20 | 30.0 |
| 2010 | Feldbrin, Zeev; Hendel, David; Lipkin, Alexander; Zin, Dan; Schorr, Louis | Achilles tendon rupture and our experience with the Achillon device. | The Israel Medical Association journal : IMAJ | 5 | 2006 | 14 | 14.3 |
| 2010 | Garrido, Ignacio Martínez; Deval, Juan Cervera; Bosch, Marta Navarro; Mediavilla, Daniel Herrero; Garcia, Vicente Pellicer; González, María Sánchez | Treatment of acute Achilles tendon ruptures with Achillon device: clinical outcomes and kinetic gait analysis. | Foot and ankle surgery : official journal of the European Society of Foot and Ankle Surgeons | 3 | 2007 | 18 | 5.6 |
| 2010 | Soubeyrand, Marc; Serra-Tosio, Géraldine; Campagna, Raphael; Molina, Véronique; Sitbon, Philippe; Biau, David J. | Intraoperative ultrasonography during percutaneous Achilles tendon repair. | Foot & ankle international | 1 | 2008 | 21 | 9.5 |
| 2011 | Saxena, Amol; Ewen, Brynn; Maffulli, Nicola | Rehabilitation of the operated achilles tendon: parameters for predicting return to activity. | The Journal of foot and ankle surgery : official publication of the American College of Foot and Ankle Surgeons | 16 | 1997 | 27 | 18.5 |
| 2011 | Demirel, Murat; Turhan, Egemen; Dereboy, Ferit; Yazar, Tarik | Augmented repair of acute tendo Achilles ruptures with gastrosoleus turn down flap. | Indian journal of orthopaedics | 12 | 1998 | 78 | 11.5 |
| 2011 | Maffulli, Nicola; Longo, Umile Giuseppe; Maffulli, Gayle D.; Khanna, Anil; Denaro, Vincenzo | Achilles tendon ruptures in diabetic patients. | Archives of Orthopaedic & Trauma Surgery | 8 | 1999 | 36 | 22.2 |
| 2011 | Maffulli, Nicola; Longo, Umile Giuseppe; Maffulli, Gayle D.; Khanna, Anil; Denaro, Vincenzo | Achilles tendon ruptures in elite athletes. | Foot & ankle international | 8 | 1999 | 17 | 23.5 |
| 2011 | Metz, Roderik; van der Heijden, Geert J. M. G.; Verleisdonk, Egbert-Jan M. M.; Kolfschoten, Nicky; Verhofstad, Michiel H. J.; van der Werken, Christiaan | Effect of complications after minimally invasive surgical repair of acute achilles tendon ruptures: report on 211 cases. | The American journal of sports medicine | 8 | 2000 | 340 | 19.4 |
| 2011 | Maffulli, Nicola; Longo, Umile Giuseppe; Maffulli, Gayle D.; Rabitti, Carla; Khanna, Anil; Denaro, Vincenzo | Marked pathological changes proximal and distal to the site of rupture in acute Achilles tendon ruptures. | Knee Surgery, Sports Traumatology, Arthroscopy | 2 | 2002 | 29 | 27.6 |
| 2011 | Gwynne-Jones, David P.; Sims, Martyn; Handcock, Deb | Epidemiology and outcomes of acute Achilles tendon rupture with operative or nonoperative treatment using an identical functional bracing protocol. | Foot & ankle international | 10 | 2003 | 363 | 45.7 |
| 2011 | Macquet, Adrian Jules; Christensen, Rosie Jane; Debenham, Matthew; Wyatt, Michael; Panting, Allan Leslie | Open repair of the acutely torn Achilles tendon under local anaesthetic. | ANZ journal of surgery | 5 | 2003 | 87 | 37.9 |
| 2011 | Saxena, Amol; Granot, Allison | Use of an anti-gravity treadmill in the rehabilitation of the operated achilles tendon: a pilot study. | The Journal of foot and ankle surgery : official publication of the American College of Foot and Ankle Surgeons | 2 | 2007 | 4 | 25.0 |
| 2011 | Schepull, T; Kvist, J; Norrman, H; Trinks, M; Berlin, G; Aspenberg, P | Autologous Platelets Have No Effect on the Healing of Human Achilles Tendon Ruptures | American journal of sports medicine | 2 | 2007 | 30 | 20.0 |
| 2012 | Horstmann, T.; Lukas, C.; Merk, J.; Brauner, T.; Mündermann, A. | Deficits 10-years after Achilles tendon repair. | International journal of sports medicine | 12 | 1985 | 63 | 23.8 |
| 2012 | Wise, Barton L.; Peloquin, Christine; Choi, Hyon; Lane, Nancy E.; Zhang, Yuqing | Impact of age, sex, obesity, and steroid use on quinolone-associated tendon disorders. | The American journal of medicine | 24 | 1997 | 7685 | 31.9 |
| 2012 | Talbot, JC; Williams, GT; Bismil, Q; Shaw, DL; Schilders, E | Results of Accelerated Postoperative Rehabilitation Using Novel "Suture Frame" Repair of Achilles Tendon Rupture | JOURNAL OF FOOT & ANKLE SURGERY | 6 | 2001 | 15 | 13.3 |
| 2012 | Bergkvist, Dan; Åström, Ingrid; Josefsson, Per-Olof; Dahlberg, Leif E. | Acute Achilles tendon rupture: a questionnaire follow-up of 487 patients. | The Journal of bone and joint surgery. American volume | 5 | 2004 | 487 | 16.0 |
| 2012 | Patel, Vishal C.; Lozano-Calderon, Santiago; McWilliam, James | Immediate weight bearing after modified percutaneous Achilles tendon repair. | Foot & ankle international | 10 | 2004 | 52 | 23.1 |
| 2012 | Garras, David N.; Raikin, Steven M.; Bhat, Suneel B.; Taweel, Nicholas; Karanjia, Homyar | MRI is unnecessary for diagnosing acute Achilles tendon ruptures: clinical diagnostic criteria. | Clinical orthopaedics and related research | 9 | 2005 | 132 | 15.2 |
| 2012 | Lapidus, Lasse J.; Ray, Bill Adams; Hamberg, Per | Medial Achilles tendon island flap--a novel technique to treat reruptures and neglected ruptures of the Achilles tendon. | International orthopaedics | 9 | 2005 | 6 | 33.3 |
| 2012 | Vadalà, A.; De Carli, A.; Vulpiani, M. C.; Iorio, R.; Vetrano, M.; Scapellato, S.; Suarez, T.; Di Salvo, F.; Ferretti, A. | Clinical, functional and radiological results of Achilles tenorraphy surgically treated with mini-open technique. | The Journal of sports medicine and physical fitness | 11 | 2005 | 80 | 22.5 |
| 2012 | Wang, Chen-Chie; Chen, Pei-Yu; Wang, Ting-Ming; Wang, Chung-Li | Ultrasound-Guided Minimally Invasive Surgery for Achilles Tendon Rupture: Preliminary Results. | Foot & Ankle International | 7 | 2005 | 48 | 16.7 |
| 2012 | Grubor, Predrag; Grubor, Milan | Treatment of Achilles tendon rupture using different methods. | Vojnosanitetski pregled | 8 | 2006 | 42 | 11.9 |
| 2012 | Henríquez, Hugo; Muñoz, Roberto; Carcuro, Giovanni; Bastías, Christian | Is percutaneous repair better than open repair in acute Achilles tendon rupture? | Clinical orthopaedics and related research | 4 | 2006 | 32 | 12.5 |
| 2012 | Jielile, Jiasharete; Sabirhazi, Gulnur; Chen, Jiangtao; Aldyarhan, Kayrat; Zheyiken, Jangannuer; Zhao, Qin; Bai, Jingping | Novel surgical technique and early kinesiotherapy for acute Achilles tendon rupture. | Foot & ankle international | 11 | 2006 | 107 | 21.5 |
| 2012 | Park, Yong-Serk; Sung, Ki-Sun | Surgical reconstruction of chronic achilles tendon ruptures using various methods. | Orthopedics | 8 | 2006 | 12 | 8.3 |
| 2012 | Sarzaeem, Mohammad Mahdi; Lemraski, Mohammad Mahdi Bagherian; Safdari, Farshad | Chronic Achilles tendon rupture reconstruction using a free semitendinosus tendon graft transfer. | Knee surgery, sports traumatology, arthroscopy : official journal of the ESSKA | 5 | 2006 | 11 | 0.0 |
| 2012 | Schepull, T.; Kvist, J.; Aspenberg, P. | Early E-modulus of healing Achilles tendons correlates with late function: similar results with or without surgery. | Scandinavian journal of medicine & science in sports | 3 | 2006 | 30 | 16.7 |
| 2012 | Valente, Maurizio; Crucil, Marina; Alecci, Vincenzo; Frezza, Giovanni | Minimally invasive repair of acute Achilles tendon ruptures with Achillon device. | Musculoskeletal surgery | 5 | 2007 | 35 | 8.6 |
| 2012 | Kearney, Rebecca S.; Achten, Juul; Lamb, Sarah E.; Parsons, Nicholas; Costa, Matthew L. | The Achilles tendon total rupture score: a study of responsiveness, internal consistency and convergent validity on patients with acute Achilles tendon ruptures. | Health and quality of life outcomes | 3 | 2008 | 64 | 25.0 |
| 2013 | Maffulli, N; Del Buono, A; Spiezia, F; Maffulli, GD; Longo, UG; Denaro, V | Less-Invasive Semitendinosus Tendon Graft Augmentation for the Reconstruction of Chronic Tears of the Achilles Tendon | AMERICAN JOURNAL OF SPORTS MEDICINE | 4 | 2002 | 26 | 11.5 |
| 2013 | Rosso, Claudio; Vavken, Patrick; Polzer, Caroline; Buckland, Daniel M.; Studler, Ueli; Weisskopf, Lukas; Lottenbach, Marc; Müller, Andreas Marc; Valderrabano, Victor | Long-term outcomes of muscle volume and Achilles tendon length after Achilles tendon ruptures. | Knee surgery, sports traumatology, arthroscopy : official journal of the ESSKA | 11 | 2003 | 52 | 21.2 |
| 2013 | Muezzinoglu, S; Memisoglu, K; Sarman, H; Aydin, A; Atmaca, H | Internal Splinting: A New Technique for Achilles Tendon Repair | TECHNIQUES IN FOOT AND ANKLE SURGERY | 14 | 2004 | 24 | 8.3 |
| 2013 | Doral, Mahmut Nedim | What is the effect of the early weight-bearing mobilisation without using any support after endoscopy-assisted Achilles tendon repair? | Knee surgery, sports traumatology, arthroscopy : official journal of the ESSKA | 14 | 2005 | 32 | 0.0 |
| 2013 | Fan, Yifang; Fan, Yubo; Li, Zhiyu; Newman, Tony; Lv, Changsheng; Zhou, Yi | Screening method based on walking plantar impulse for detecting musculoskeletal senescence and injury. | PloS one | 12 | 2005 | 7 | 0.0 |
| 2013 | Jackson, Gillian; Sinclair, Victoria F.; McLaughlin, Charles; Barrie, James | Outcomes of functional weight-bearing rehabilitation of Achilles tendon ruptures. | Orthopedics | 7 | 2005 | 80 | 23.8 |
| 2013 | Chih-Hao Chiu; Wen-Lin Yeh; Min-Chien Tsai; Shih-Sheng Chang; Kuo-Yao Hsu; Yi-Sheng Chan | Endoscopy-Assisted Percutaneous Repair of Acute Achilles Tendon Tears. | Foot & Ankle International | 4 | 2007 | 19 | 5.3 |
| 2013 | Chiu, Chih-Hao; Yeh, Wen-Lin; Tsai, Min-Chien; Chang, Shih-Sheng; Hsu, Kuo-Yao; Chan, Yi-Sheng | Endoscopy-assisted percutaneous repair of acute Achilles tendon tears. | Foot & ankle international | 4 | 2007 | 19 | 5.3 |
| 2013 | Guillo, Stephane; Del Buono, Angelo; Dias, Marion; Denaro, Vincenzo; Maffulli, Nicola | Percutaneous repair of acute ruptures of the tendo Achillis. | The surgeon : journal of the Royal Colleges of Surgeons of Edinburgh and Ireland | 2 | 2007 | 23 | 8.7 |
| 2013 | Jallageas, R.; Bordes, J.; Daviet, J.-C.; Mabit, C.; Coste, C. | Evaluation of surgical treatment for ruptured Achilles tendon in 31 athletes. | Orthopaedics & traumatology, surgery & research : OTSR | 5 | 2007 | 31 | 16.1 |
| 2013 | Khiami, F.; Di Schino, M.; Sariali, E.; Cao, D.; Rolland, E.; Catonné, Y. | Treatment of chronic Achilles tendon rupture by shortening suture and free sural triceps aponeurosis graft. | Orthopaedics & traumatology, surgery & research : OTSR | 6 | 2007 | 23 | 13.0 |
| 2013 | Carmont, M. R.; Heaver, C.; Pradhan, A.; Mei-Dan, O.; Gravare Silbernagel, K. | Surgical repair of the ruptured Achilles tendon: the cost-effectiveness of open versus percutaneous repair. | Knee surgery, sports traumatology, arthroscopy : official journal of the ESSKA | 7 | 2008 | 84 | 16.7 |
| 2013 | Lins, C; Ninomya, AF; Bittar, CK; de Carvalho, AE; Cliquet, A | Kinetic and Kinematic Evaluation of the Ankle Joint After Achilles Tendon Reconstruction With Free Semitendinosus Tendon Graft: Preliminary Results | ARTIFICIAL ORGANS | 2 | 2008 | 13 | 23.1 |
| 2013 | Carmont, Michael R.; Silbernagel, Karin Grävare; Nilsson-Helander, Katarina; Mei-Dan, Omer; Karlsson, Jon; Maffulli, Nicola | Cross cultural adaptation of the Achilles tendon Total Rupture Score with reliability, validity and responsiveness evaluation. | Knee surgery, sports traumatology, arthroscopy : official journal of the ESSKA | 2 | 2009 | 49 | 26.5 |
| 2013 | Kołodziej, Lukas; Bohatyrewicz, Andrzej; Kromuszczyńska, Justyna; Jezierski, Jarosław; Biedroń, Maciej | Efficacy and complications of open and minimally invasive surgery in acute Achilles tendon rupture: a prospective randomised clinical study--preliminary report. | International orthopaedics | 3 | 2009 | 47 | 4.3 |
| 2013 | Maffulli, Nicola; Loppini, Mattia; Longo, Umile Giuseppe; Maffulli, Gayle D.; Denaro, Vincenzo | Minimally invasive reconstruction of chronic achilles tendon ruptures using the ipsilateral free semitendinosus tendon graft and interference screw fixation. | The American journal of sports medicine | 3 | 2009 | 28 | 25.0 |
| 2013 | Makhdom, Asim M.; Cota, Adam; Saran, Neil; Chaytor, Ruth | Incidence of symptomatic deep venous thrombosis after Achilles tendon rupture. | The Journal of foot and ankle surgery : official publication of the American College of Foot and Ankle Surgeons | 7 | 2009 | 115 | 13.9 |
| 2013 | McNair, Peter; Nordez, Antoine; Olds, Margie; Young, Simon W.; Cornu, Christophe | Biomechanical properties of the plantar flexor muscle-tendon complex 6 months post-rupture of the Achilles tendon. | Journal of orthopaedic research : official publication of the Orthopaedic Research Society | 3 | 2009 | 30 | 50.0 |
| 2013 | Olsson, Nicklas; Silbernagel, Karin Grävare; Eriksson, Bengt I.; Sansone, Mikael; Brorsson, Annelie; Nilsson-Helander, Katarina; Karlsson, Jón | Stable surgical repair with accelerated rehabilitation versus nonsurgical treatment for acute Achilles tendon ruptures: a randomized controlled study. | The American journal of sports medicine | 2 | 2009 | 100 | 14.0 |
| 2013 | Carmont, Michael R.; Silbernagel, Karin Grävare; Edge, Antonia; Mei-Dan, Omer; Karlsson, Jón; Maffulli, Nicola | Functional Outcome of Percutaneous Achilles Repair: Improvements in Achilles Tendon Total Rupture Score During the First Year. | Orthopaedic journal of sports medicine | 4 | 2010 | 73 | 17.8 |
| 2013 | Domeij-Arverud, E.; Latifi, A.; Labruto, F.; Nilsson, G.; Ackermann, P. W. | Can foot compression under a plaster cast prevent deep-vein thrombosis during lower limb immobilisation? | The bone & joint journal | 1 | 2010 | 24 | 8.3 |
| 2013 | Ganestam, Ann; Barfod, Kristoffer; Klit, Jakob; Troelsen, Anders | Validity and reliability of the Achilles tendon total rupture score. | The Journal of foot and ankle surgery : official publication of the American College of Foot and Ankle Surgeons | 4 | 2010 | 90 | 30.0 |
| 2013 | Keyhani, Sohrab; Mardani-Kivi, Mohsen; Abbasian, Mohammadreza; Emami-Moghaddam Tehrani, Mohammad; Lahiji, Farivar Abdollahzadeh | Achilles tendon repair, a modified technique. | The archives of bone and joint surgery | 3 | 2010 | 30 | 13.3 |
| 2013 | Orr, Justin D.; McCriskin, Brendan; Dutton, Jason R. | Achillon mini-open Achilles tendon repair: early outcomes and return to duty results in U.S. military service members. | Journal of surgical orthopaedic advances | 4 | 2010 | 15 | 0.0 |
| 2013 | Schepull, Thorsten; Aspenberg, Per | Early controlled tension improves the material properties of healing human achilles tendons after ruptures: a randomized trial. | The American journal of sports medicine | 3 | 2010 | 35 | 14.3 |
| 2013 | Ozsoy, Mehmet Hakan; Cengiz, Bertan; Ozsoy, Arzu; Aksekili, Mehmet Atif Erol; Yucel, Mehmet; Fakioglu, Onur; Dincel, Veysel Ercan; Aydogan, Nevres Hurriyet | Minimally invasive Achilles tendon repair: a modification of the Achillon technique. | Foot & ankle international | 4 | 2011 | 13 | 7.7 |
| 2014 | Scott, Alex; Grewal, Navdeep; Guy, Pierre | The seasonal variation of Achilles tendon ruptures in Vancouver, Canada: a retrospective study. | BMJ open | 24 | 1998 | 543 | 17.5 |
| 2014 | Ding, Wen-Ge; Li, Huan; Zhu, Ya-Ping; Liu, Zhi-wei | Comparison between tenocutaneous suture and Kessler suture techniques in treating acute closed Achilles tendon rupture. | Foot and ankle surgery : official journal of the European Society of Foot and Ankle Surgeons | 16 | 2000 | 33 | 33.3 |
| 2014 | Porter, David A.; Barnes, Adam F.; Rund, Angela M.; Kaz, Ari J.; Tyndall, James A.; Millis, Andrew A. | Acute achilles tendon repair: strength outcomes after an acute bout of exercise in recreational athletes. | Foot & ankle international | 9 | 2000 | 40 | 10.0 |
| 2014 | Bevoni, R.; Angelini, A.; D'Apote, G.; Berti, L.; Fusaro, I.; Ellis, S.; Schuh, R.; Girolami, M. | Long term results of acute Achilles repair with triple-bundle technique and early rehabilitation protocol. | Injury | 13 | 2001 | 66 | 15.2 |
| 2014 | Ceccarelli, Francesco; Calderazzi, Filippo; Pedrazzi, Giuseppe | Is there a relation between AOFAS ankle-hindfoot score and SF-36 in evaluation of Achilles ruptures treated by percutaneous technique? | The Journal of foot and ankle surgery : official publication of the American College of Foot and Ankle Surgeons | 4 | 2006 | 17 | 17.6 |
| 2014 | Huttunen, Tuomas T.; Kannus, Pekka; Rolf, Christer; Felländer-Tsai, Li; Mattila, Ville M. | Acute achilles tendon ruptures: incidence of injury and surgery in Sweden between 2001 and 2012. | The American journal of sports medicine | 12 | 2006 | 27702 | 20.7 |
| 2014 | Esenyel, Cem Zeki; Tekin, Cagri; Cakar, Murat; Bayraktar, Kursat; Saygili, Selcuk; Esenyel, Meltem; Tekin, Zeynep N. | Surgical treatment of the neglected achilles tendon rupture with Hyalonect. | Journal of the American Podiatric Medical Association | 4 | 2008 | 10 | 0.0 |
| 2014 | Keller, Andres; Ortiz, Cristian; Wagner, Emilio; Wagner, Pablo; Mococain, Pablo | Mini-open tenorrhaphy of acute Achilles tendon ruptures: medium-term follow-up of 100 cases. | The American journal of sports medicine | 7 | 2008 | 100 | 9.0 |
| 2014 | Tejwani, Nirmal C.; Lee, James; Weatherall, Justin; Sherman, Orrin | Acute achilles tendon ruptures: a comparison of minimally invasive and open approach repairs followed by early rehabilitation. | American journal of orthopedics (Belle Mead, N.J.) | 5 | 2008 | 63 | 9.5 |
| 2014 | Young, Simon W.; Patel, Alpesh; Zhu, Mark; van Dijck, Stephanie; McNair, Peter; Bevan, Wesley P.; Tomlinson, Matthew | Weight-Bearing in the Nonoperative Treatment of Acute Achilles Tendon Ruptures: A Randomized Controlled Trial. | The Journal of bone and joint surgery. American volume | 3 | 2008 | 84 | 48.8 |
| 2014 | Arslan, Armağan; Çepni, Serdar Kamil; Sahinkaya, Türker; May, Cüneyt; Mutlu, Harun; Parmaksızoğlu, Atilla Sancar | Functional outcomes of repair of Achilles tendon using a biological open surgical method. | Acta orthopaedica et traumatologica turcica | 4 | 2009 | 22 | 0.0 |
| 2014 | Eid, Abdelsalam | Miniopen Repair of Ruptured Achilles Tendon in Diabetic Patients. | International scholarly research notices | 4 | 2009 | 13 | 69.2 |
| 2014 | Kaniki, Nicole; Willits, Kevin; Mohtadi, Nicholas G. H.; Fung, Vincent; Bryant, Dianne | A retrospective comparative study with historical control to determine the effectiveness of platelet-rich plasma as part of nonoperative treatment of acute achilles tendon rupture. | Arthroscopy : the journal of arthroscopic & related surgery : official publication of the Arthroscopy Association of North America and the International Arthroscopy Association | 3 | 2009 | 73 | 19.2 |
| 2014 | Karabinas, Panagiotis K.; Benetos, Ioannis S.; Lampropoulou-Adamidou, Kalliopi; Romoudis, Pavlos; Mavrogenis, Andreas F.; Vlamis, John | Percutaneous versus open repair of acute Achilles tendon ruptures. | European journal of orthopaedic surgery & traumatology : orthopedie traumatologie | 5 | 2009 | 34 | 17.6 |
| 2014 | Maffulli, N.; Del Buono, A.; Loppini, M.; Denaro, V. | Ipsilateral free semitendinosus tendon graft with interference screw fixation for minimally invasive reconstruction of chronic tears of the Achilles tendon. | Operative Orthopadie und Traumatologie | 3 | 2009 | 28 | 25.0 |
| 2014 | Vadalà, Antonio; Lanzetti, Riccardo Maria; Ciompi, Alessandro; Rossi, Cristina; Lupariello, Domenico; Ferretti, Andrea | Functional evaluation of professional athletes treated with a mini-open technique for achilles tendon rupture. | Muscles, ligaments and tendons journal | 3 | 2009 | 36 | 8.3 |
| 2014 | Lacoste, S.; Féron, J. M.; Cherrier, B. | Percutaneous Tenolig(®) repair under intra-operative ultrasonography guidance in acute Achilles tendon rupture. | Orthopaedics & traumatology, surgery & research : OTSR | 5 | 2010 | 75 | 20.0 |
| 2014 | Barfod, Kristoffer Weisskirchner; Bencke, Jesper; Lauridsen, Hanne Bloch; Ban, Ilija; Ebskov, Lars; Troelsen, Anders | Nonoperative dynamic treatment of acute achilles tendon rupture: the influence of early weight-bearing on clinical outcome: a blinded, randomized controlled trial. | The Journal of bone and joint surgery. American volume | 2 | 2011 | 57 | 15.8 |
| 2014 | Dumbre Patil, Sampat Shivajirao; Dumbre Patil, Vaishali Sampat; Basa, Vikas Rajeshwarrao; Dombale, Ajay Birappa | Semitendinosus Tendon Autograft for Reconstruction of Large Defects in Chronic Achilles Tendon Ruptures. | Foot & ankle international | 3 | 2011 | 35 | 42.9 |
| 2015 | Lantto, I.; Heikkinen, J.; Flinkkilä, T.; Ohtonen, P.; Leppilahti, J. | Epidemiology of Achilles tendon ruptures: increasing incidence over a 33-year period. | Scandinavian journal of medicine & science in sports | 33 | 1995 | 515 | 11.5 |
| 2015 | Todorov, Atanas; Schaub, Frederic; Blanke, Fabian; Heisterbach, Patricia; Sachser, Franciska; Gösele, Andreas; Majewski, Martin | Clinical assessment is sufficient to allow outcome evaluation following surgical management of Achilles tendon ruptures. | Muscles, ligaments and tendons journal | 11 | 2001 | 74 | 21.6 |
| 2015 | Heyes, G. J.; Tucker, A.; Michael, A. L. R.; Wallace, R. G. H. | The incidence of deep vein thrombosis and pulmonary embolism following cast immobilisation and early functional bracing of Tendo Achilles rupture without thromboprophylaxis. | European journal of trauma and emergency surgery : official publication of the European Trauma Society | 13 | 2002 | 949 | 27.1 |
| 2015 | Mavrodontidis, Alexandros; Lykissas, Marios; Koulouvaris, Panayiotis; Pafilas, Dimitrios; Kontogeorgakos, Vasilios; Zalavras, Charalampos | Percutaneous repair of acute Achilles tendon rupture: a functional evaluation study with a minimum 10-year follow-up. | Acta orthopaedica et traumatologica turcica | 5 | 2002 | 11 | 0.0 |
| 2015 | Rosso, Claudio; Buckland, Daniel M.; Polzer, Caroline; Sadoghi, Patrick; Schuh, Reinhard; Weisskopf, Lukas; Vavken, Patrick; Valderrabano, Victor | Long-term biomechanical outcomes after Achilles tendon ruptures. | Knee surgery, sports traumatology, arthroscopy : official journal of the ESSKA | 11 | 2003 | 52 | 21.2 |
| 2015 | Knobe, Matthias; Gradl, Gertraud; Klos, Kajetan; Corsten, Johannes; Dienstknecht, Thomas; Rath, Bjoern; Sönmez, Tolga Taha; Hoeckle, Christian; Pape, Hans-Christoph | Is percutaneous suturing superior to open fibrin gluing in acute Achilles tendon rupture? | International orthopaedics | 10 | 2004 | 64 | 14.1 |
| 2015 | Cukelj, Fabijan; Bandalovic, Ante; Knezevic, Josip; Pavic, Arsen; Pivalica, Bozen; Bakota, Bore | Treatment of ruptured Achilles tendon: Operative or non-operative procedure? | Injury | 16 | 2005 | 90 | 20.0 |
| 2015 | Maffulli, N.; Oliva, F.; Costa, V.; Del Buono, A. | The management of chronic rupture of the Achilles tendon: minimally invasive peroneus brevis tendon transfer. | The bone & joint journal | 6 | 2006 | 17 | 17.6 |
| 2015 | Geremia, Jeam Marcel; Bobbert, Maarten Frank; Casa Nova, Mayra; Ott, Rafael Duvelius; Lemos, Fernando de Aguiar; Lupion, Raquel de Oliveira; Frasson, Viviane Bortoluzzi; Vaz, Marco Aurélio | The structural and mechanical properties of the Achilles tendon 2 years after surgical repair. | Clinical biomechanics (Bristol, Avon) | 2 | 2008 | 18 | 0.0 |
| 2015 | Ji, Yunhan; Ma, Xin; Wang, Xu; Huang, Jiazhang; Zhang, Chao; Chen, Li | Different Sutures in the Surgical Treatment of Acute Closed Achilles Tendon Rupture. | The Indian journal of surgery | 5 | 2008 | 128 | 25.8 |
| 2015 | Sarman, Hakan; Atmaca, Halil; Cakir, Ozgur; Muezzinoglu, Umit Sefa; Anik, Yonca; Memisoglu, Kaya; Baran, Tuncay; Isik, Cengiz | Assessment of Postoperative Tendon Quality in Patients With Achilles Tendon Rupture Using Diffusion Tensor Imaging and Tendon Fiber Tracking. | The Journal of foot and ankle surgery : official publication of the American College of Foot and Ankle Surgeons | 8 | 2008 | 16 | 18.8 |
| 2015 | Hsu, Andrew R.; Jones, Carroll P.; Cohen, Bruce E.; Davis, W. Hodges; Ellington, J. Kent; Anderson, Robert B. | Clinical Outcomes and Complications of Percutaneous Achilles Repair System Versus Open Technique for Acute Achilles Tendon Ruptures. | Foot & ankle international | 10 | 2009 | 267 | 13.5 |
| 2015 | Kaya Mutlu, Ebru; Celik, Derya; Kiliçoglu, Önder; Ozdincler, Arzu Razak; Nilsson-Helander, Katarina | The Turkish version of the Achilles tendon Total Rupture Score: cross-cultural adaptation, reliability and validity. | Knee surgery, sports traumatology, arthroscopy : official journal of the ESSKA | 9 | 2009 | 74 | 1.4 |
| 2015 | Marican, Mohd Mizan; Fook-Chong, Stephanie Man Chung; Rikhraj, Inderjeet Singh | Incidence of postoperative wound infections after open tendo Achilles repairs. | Singapore medical journal | 3 | 2009 | 60 | 45.0 |
| 2015 | Korkmaz, Murat; Erkoc, Mustafa Fatih; Yolcu, Sadiye; Balbaloglu, Ozlem; Öztemur, Zekeriya; Karaaslan, Fatih | Weight bearing the same day versus non-weight bearing for 4 weeks in Achilles tendon rupture. | Journal of orthopaedic science : official journal of the Japanese Orthopaedic Association | 7 | 2010 | 47 | 17.0 |
| 2015 | Porter, Mark D.; Shadbolt, Bruce | Randomized controlled trial of accelerated rehabilitation versus standard protocol following surgical repair of ruptured Achilles tendon. | ANZ journal of surgery | 3 | 2010 | 51 | 17.6 |
| 2015 | Al-Mouazzen, Louay; Rajakulendran, Karthig; Najefi, Ali; Ahad, Nurul | Percutaneous repair followed by accelerated rehabilitation for acute Achilles tendon ruptures. | Journal of orthopaedic surgery (Hong Kong) | 2 | 2011 | 30 | 30.0 |
| 2015 | Sadek, Ahmed F.; Fouly, Ezzat H.; Laklok, Mohammed A.; Amin, Mohammed F. | Functional and MRI follow-up after reconstruction of chronic ruptures of the Achilles tendon Myerson type III using the triple-loop plantaris tendon wrapped with central turndown flap: a case series. | Journal of orthopaedic surgery and research | 5 | 2011 | 18 | 77.8 |
| 2015 | Carmont, Michael R.; Grävare Silbernagel, Karin; Brorsson, Annelie; Olsson, Nicklas; Maffulli, Nicola; Karlsson, Jon | The Achilles tendon resting angle as an indirect measure of Achilles tendon length following rupture, repair, and rehabilitation. | Asia-Pacific journal of sports medicine, arthroscopy, rehabilitation and technology | 2 | 2012 | 26 | 34.6 |
| 2015 | Chen, Hua; Ji, Xinran; Zhang, Qun; Liang, Xiangdang; Tang, Peifu | Channel-assisted minimally invasive repair of acute Achilles tendon rupture. | Journal of orthopaedic surgery and research | 3 | 2012 | 82 | 31.7 |
| 2015 | Domeij-Arverud, E.; Labruto, F.; Latifi, A.; Nilsson, G.; Edman, G.; Ackermann, P. W. | Intermittent pneumatic compression reduces the risk of deep vein thrombosis during post-operative lower limb immobilisation: a prospective randomised trial of acute ruptures of the Achilles tendon. | The bone & joint journal | 3 | 2012 | 140 | 15.0 |
| 2015 | Huang, Xiaowei; Huang, Gan; Ji, Ying; Ao, Rong guang; Yu, Baoqing; Zhu, Ya Long | Augmented Repair of Acute Achilles Tendon Rupture Using an Allograft Tendon Weaving Technique. | The Journal of foot and ankle surgery : official publication of the American College of Foot and Ankle Surgeons | 3 | 2012 | 59 | 10.2 |
| 2015 | Reddy, AK; Srinivas, B; Prashanth, V; Reddy, BKK; Rao, KCS | MANAGEMENT OF CHRONIC ACHILLES TENDON RUPTURES BY RECONSTRUCTING WITH FLEXOR HALLUCIS LONGUS AND PERONEUS BREVIS TENDONS: A COMPARATIVE STUDY | JOURNAL OF EVOLUTION OF MEDICAL AND DENTAL SCIENCES-JEMDS | 5 | 2012 | 20 | 25.0 |
| 2015 | Wang, Baocang; Feng, Xiaona; Yan, Ming; Wang, Hui; Li, Yong | Application of lariat lock catch knot suture in the achilles tendon rupture. | International journal of clinical and experimental medicine | 4 | 2012 | 32 | 18.8 |
| 2016 | Guclu, Berk; Basat, H. Cagdas; Yildirim, Tugrul; Bozduman, Omer; Us, Ali Kemal | Long-term Results of Chronic Achilles Tendon Ruptures Repaired With V-Y Tendon Plasty and Fascia Turndown. | Foot & ankle international | 7 | 1998 | 17 | 29.4 |
| 2016 | Seker, A; Kara, A; Armagan, R; Oc, Y; Varol, A; Sezer, HB | Reconstruction of neglected achilles tendon ruptures with gastrocnemius flaps: excellent results in long-term follow-up | ARCHIVES OF ORTHOPAEDIC AND TRAUMA SURGERY | 16 | 2002 | 21 | 0.0 |
| 2016 | Ganestam, Ann; Kallemose, Thomas; Troelsen, Anders; Barfod, Kristoffer Weisskirchner | Increasing incidence of acute Achilles tendon rupture and a noticeable decline in surgical treatment from 1994 to 2013. A nationwide registry study of 33,160 patients. | Knee surgery, sports traumatology, arthroscopy : official journal of the ESSKA | 20 | 2003 | 33160 | 24.8 |
| 2016 | Ecker, Timo M.; Bremer, Anne K.; Krause, Fabian G.; Müller, Thorsten; Weber, Martin | Prospective Use of a Standardized Nonoperative Early Weightbearing Protocol for Achilles Tendon Rupture: 17 Years of Experience. | The American journal of sports medicine | 18 | 2004 | 171 | 26.3 |
| 2016 | Sarman, Hakan; Muezzinoglu, Umit Sefa; Memisoglu, Kaya; Aydin, Adem; Atmaca, Halil; Baran, Tuncay; Odabas Ozgur, Bahar; Ozgur, Turgay; Kantar, Cengizhan | Comparison of Semi-Invasive "Internal Splinting" and Open Suturing Techniques in Achilles Tendon Rupture Surgery. | The Journal of foot and ankle surgery : official publication of the American College of Foot and Ankle Surgeons | 14 | 2004 | 40 | 10.0 |
| 2016 | Taşatan, Ersin; Emre, Tuluhan Yunus; Demircioğlu, Demet Tekdöş; Demiralp, Bahtiyar; Kırdemir, Vecihi | Long-Term Results of Mini-Open Repair Technique in the Treatment of Acute Achilles Tendon Rupture: A Prospective Study. | The Journal of foot and ankle surgery : official publication of the American College of Foot and Ankle Surgeons | 6 | 2005 | 20 | 10.0 |
| 2016 | Yasuda, Toshito; Shima, Hiroaki; Mori, Katsunori; Kizawa, Momoko; Neo, Masashi | Direct Repair of Chronic Achilles Tendon Ruptures Using Scar Tissue Located Between the Tendon Stumps. | The Journal of bone and joint surgery. American volume | 12 | 2006 | 30 | 46.7 |
| 2016 | Saper, David; Lybrand, Kyle; Creevy, William; Li, Xinning | Using a Posterior Compartment Fasciotomy and Paratenon Closure in Acute Achilles Tendon Repair. | Orthopedics | 11 | 2007 | 82 | 8.5 |
| 2016 | Ozer, Hamza; Selek, Hakan Y.; Harput, Gulcan; Oznur, Ali; Baltaci, Gul | Achilles Tendon Open Repair Augmented With Distal Turndown Tendon Flap and Posterior Crural Fasciotomy. | The Journal of foot and ankle surgery : official publication of the American College of Foot and Ankle Surgeons | 7 | 2008 | 23 | 4.3 |
| 2016 | Jielile, Jiasharete; Badalihan, Ayinazi; Qianman, Bayixiati; Satewalede, Tuerde; Wuerliebieke, Jianati; Kelamu, Mailamuguli; Jialihasi, Ayidaer | Clinical outcome of exercise therapy and early post-operative rehabilitation for treatment of neglected Achilles tendon rupture: a randomized study. | Knee surgery, sports traumatology, arthroscopy : official journal of the ESSKA | 6 | 2009 | 57 | 15.8 |
| 2016 | Lin, Yangjing; Yang, Liu; Yin, Li; Duan, Xiaojun | Surgical Strategy for the Chronic Achilles Tendon Rupture. | BioMed research international | 12 | 2009 | 29 | 20.7 |
| 2016 | Zambelli, Roberto; Pinto, Rafael Z.; Magalhães, João Murilo Brandão; Lopes, Fernando Araujo Silva; Castilho, Rodrigo Simões; Baumfeld, Daniel; Dos Santos, Thiago Ribeiro Teles; Maffulli, Nicola | Development of the Brazilian Portuguese version of the Achilles Tendon Total Rupture Score (ATRS BrP): a cross-cultural adaptation with reliability and construct validity evaluation. | BMC sports science, medicine & rehabilitation | 6 | 2009 | 46 | 15.2 |
| 2016 | Ofili, Kene P.; Pollard, Jason D.; Schuberth, John M. | The Neglected Achilles Tendon Rupture Repaired With Allograft: A Review of 14 Cases. | The Journal of foot and ankle surgery : official publication of the American College of Foot and Ankle Surgeons | 7 | 2010 | 14 | 42.9 |
| 2016 | Zhang, Li-ning; Wan, Wen-bo; Wang, Yue-xiang; Jiao, Zi-yu; Zhang, Li-hai; Luo, Yu-kun; Tang, Pei-fu | Evaluation of Elastic Stiffness in Healing Achilles Tendon After Surgical Repair of a Tendon Rupture Using In Vivo Ultrasound Shear Wave Elastography. | Medical science monitor : international medical journal of experimental and clinical research | 3 | 2010 | 26 | 19.2 |
| 2016 | Aujla, Randeep; Kumar, Amit; Bhatia, Maneesh | Non-surgical treatment of Achilles rupture: Does duration in functional weight bearing orthosis matter? | Foot and ankle surgery : official journal of the European Society of Foot and Ankle Surgeons | 3 | 2011 | 88 | 18.2 |
| 2016 | De Carli, Angelo; Lanzetti, Riccardo Maria; Ciompi, Alessandro; Lupariello, Domenico; Vadalà, Antonio; Argento, Giuseppe; Ferretti, Andrea; Vulpiani, M. C.; Vetrano, M. | Can platelet-rich plasma have a role in Achilles tendon surgical repair? | Knee surgery, sports traumatology, arthroscopy : official journal of the ESSKA | 1 | 2011 | 30 | 20.0 |
| 2016 | Fox, G.; Gabbe, B. J.; Richardson, M.; Oppy, A.; Page, R.; Edwards, E. R.; Hau, R.; Ekegren, C. L. | Twelve-month outcomes following surgical repair of the Achilles tendon. | Injury | 6 | 2011 | 3184 | 27.4 |
| 2016 | Kocialkowski, C.; Javed, S.; Rachha, R.; Shoaib, A. | Reduced length of stay with minimally invasive repair of ruptured achilles tendon. | Acta orthopaedica Belgica | 3 | 2011 | 65 | 16.9 |
| 2016 | Lantto, Iikka; Heikkinen, Juuso; Flinkkila, Tapio; Ohtonen, Pasi; Siira, Pertti; Laine, Vesa; Leppilahti, Juhana | A Prospective Randomized Trial Comparing Surgical and Nonsurgical Treatments of Acute Achilles Tendon Ruptures. | The American journal of sports medicine | 5 | 2011 | 60 | 8.3 |
| 2016 | MacMahon, Aoife; Deland, Jonathan T.; Do, Huong; Soukup, Dylan S.; Sofka, Carolyn M.; Demetracopolous, Constantine A.; DeBlis, Ryan | MRI Evaluation of Achilles Tendon Rotation and Sural Nerve Anatomy: Implications for Percutaneous and Limited-Open Achilles Tendon Repair. | Foot & ankle international | 6 | 2011 | 21 | 4.8 |
| 2016 | MacMahon, Aoife; Deland, Jonathan T.; Do, Huong; Soukup, Dylan S.; Sofka, Carolyn M.; Demetracopolous, Constantine A.; DeBlis, Ryan | MRI Evaluation of Achilles Tendon Rotation and Sural Nerve Anatomy. | Foot & Ankle International | 6 | 2011 | 21 | 4.8 |
| 2016 | Ozkan, H.; Ege, T.; Koca, K.; Can, N.; Yurttas, Y.; Tunay, S. | Pedobarographic measurements after repair of Achilles tendon by minimal invasive surgery. | Acta orthopaedica Belgica | 3 | 2011 | 15 | 0.0 |
| 2016 | Busilacchi, A.; Olivieri, M.; Ulisse, S.; Gesuita, R.; Skrami, E.; Lording, T.; Fusini, F.; Gigante, A. | Real-time sonoelastography as novel follow-up method in Achilles tendon surgery. | Knee surgery, sports traumatology, arthroscopy : official journal of the ESSKA | 3 | 2012 | 25 | 12.0 |
| 2016 | Daghino, W.; Enrietti, E.; Sprio, A. E.; di Prun, N. Barbasetti; Berta, G. N.; Massè, A. | Subcutaneous Achilles tendon rupture: A comparison between open technique and mini-invasive tenorrhaphy with Achillon(®) suture system. | Injury | 5 | 2012 | 140 | 15.0 |
| 2016 | Liu, CC; Cui, CW; Pan, ZH; Feng, WL | Effects of evolutionary "(sic)"- shaped incision on surgical treatment for achilles tendon rupture | INTERNATIONAL JOURNAL OF CLINICAL AND EXPERIMENTAL MEDICINE | 3 | 2012 | 19 | 15.8 |
| 2016 | Renninger, Christopher H.; Kuhn, Kevin; Fellars, Todd; Youngblood, Scot; Bellamy, Joseph | Operative and Nonoperative Management of Achilles Tendon Ruptures in Active Duty Military Population. | Foot & ankle international | 4 | 2012 | 57 | 0.0 |
| 2016 | Zou, J; Mo, XL; Shi, ZM; Li, TZ; Xue, JF; Mei, GH; Li, XL | A Prospective Study of Platelet-Rich Plasma as Biological Augmentation for Acute Achilles Tendon Rupture Repair | BIOMED RESEARCH INTERNATIONAL | 2 | 2013 | 36 | 2.8 |
| 2017 | Lonzarić, Dragan; Kruščić, Aleksandar; Dinevski, Dejan; Povalej Bržan, Petra; Jesenšek Papež, Breda | Primary surgical repair of acute Achilles tendon rupture: comparative results of three surgical techniques. | Wiener klinische Wochenschrift | 9 | 2004 | 262 | 6.9 |
| 2017 | Leigheb, Massimiliano; Guzzardi, Giuseppe; Pogliacomi, Francesco; Sempio, Luca; Grassi, Federico A. | Comparison of clinical results after augmented versus direct surgical repair of acute Achilles tendon rupture. | Acta bio-medica : Atenei Parmensis | 16 | 2007 | 90 | 4.4 |
| 2017 | Maffulli, Gayle; Buono, Angelo Del; Richards, Paula; Oliva, Francesco; Maffulli, Nicola | Conservative, minimally invasive and open surgical repair for management of acute ruptures of the Achilles tendon: a clinical and functional retrospective study. | Muscles, ligaments and tendons journal | 2 | 2007 | 23 | 8.7 |
| 2017 | Sheth, U.; Wasserstein, D.; Jenkinson, R.; Moineddin, R.; Kreder, H.; Jaglal, S. | Practice patterns in the care of acute Achilles tendon ruptures : is there an association with level I evidence? | The bone & joint journal | 13 | 2008 | 29531 | 33.6 |
| 2017 | Rensing, Nicholas; Waterman, Brian R.; Frank, Rachel M.; Heida, Kenneth A.; Orr, Justin D. | Low Risk for Local and Systemic Complications After Primary Repair of 1626 Achilles Tendon Ruptures. | Foot & ankle specialist | 10 | 2009 | 1626 | 24.2 |
| 2017 | Ahmad, Jamal; Jones, Kennis | The Effect of Obesity on Surgical Treatment of Achilles Tendon Ruptures. | The Journal of the American Academy of Orthopaedic Surgeons | 9 | 2010 | 76 | 19.7 |
| 2017 | Zayni, Richard; Coursier, Raphaël; Zakaria, Moudasser; Desrousseaux, Jean-François; Cordonnier, Denis; Polveche, Gilles | Activity level recovery after acute Achilles tendon rupture surgically repaired: a series of 29 patients with a mean follow-up of 46 months. | Muscles, ligaments and tendons journal | 6 | 2010 | 29 | 17.2 |
| 2017 | Toyooka, Seikai; Takeda, Hideki; Nakajima, Kohei; Masujima, Atsushi; Miyamoto, Wataru; Pagliazzi, Gherardo; Nakagawa, Takumi; Kawano, Hirotaka | Correlation Between Recovery of Triceps Surae Muscle Strength and Level of Activity After Open Repair of Acute Achilles Tendon Rupture. | Foot & ankle international | 5 | 2011 | 96 | 24.0 |
| 2017 | Miyamoto, Wataru; Imade, Shinji; Innami, Ken; Kawano, Hirotaka; Takao, Masato | Acute Achilles Tendon Rupture Treated by Double Side-Locking Loop Suture Technique With Early Rehabilitation. | Foot & ankle international | 4 | 2012 | 44 | 43.2 |
| 2017 | Obut, Sinan; Gultekin, Alper; Unal, Meric; Serarslan, Ulaş; Tuhanioğlu, Ümit | A simple suture-guiding device for minimally invasive Achilles tendon repair. | Journal of orthopaedic surgery (Hong Kong) | 3 | 2012 | 20 | 5.0 |
| 2017 | Ozan, Firat; Dogar, Fatih; Gurbuz, Kaan; Ekinci, Yakup; Koyuncu, Semmi; Sekban, Hazim | Chronic Achilles Tendon Rupture Reconstruction Using the Lindholm Method and the Vulpius Method. | Journal of clinical medicine research | 6 | 2012 | 15 | 13.3 |
| 2017 | Shoaib, Ahmed; Mishra, Viren | Surgical repair of symptomatic chronic achilles tendon rupture using synthetic graft augmentation. | Foot and ankle surgery : official journal of the European Society of Foot and Ankle Surgeons | 2 | 2012 | 5 | 60.0 |
| 2017 | Lawrence, J. E.; Nasr, P.; Fountain, D. M.; Berman, L.; Robinson, A. H. N. | Functional outcomes of conservatively managed acute ruptures of the Achilles tendon. | The bone & joint journal | 4 | 2013 | 38 | 23.7 |
| 2017 | Park, Young Hwan; Jeong, Seong Min; Choi, Gi Won; Kim, Hak Jun | How early must an acute Achilles tendon rupture be repaired? | Injury | 5 | 2013 | 65 | 12.3 |
| 2017 | Carmont, Michael R.; Zellers, Jennifer A.; Brorsson, Annelie; Olsson, Nicklas; Nilsson-Helander, Katarina; Karlsson, Jon; Silbernagel, Karin Grävare | Functional Outcomes of Achilles Tendon Minimally Invasive Repair Using 4- and 6-Strand Nonabsorbable Suture: A Cohort Comparison Study. | Orthopaedic journal of sports medicine | 3 | 2014 | 65 | 18.5 |
| 2017 | Baumfeld, Daniel; Baumfeld, Tiago; Figueiredo, André Rocha; de Araujo Junior, Luis Fernando; Macedo, Benjamim; Silva, Thiago Alexandre Alves; Raduan, Fernando; Nery, Caio | Endoscopic Flexor Halluces Longus transfer for Chronic Achilles Tendon rupture - technique description and early post-operative results. | Muscles, ligaments and tendons journal | 2 | 2015 | 6 | 33.3 |
| 2017 | Minkwitz, Susann; Schmock, Aysha; Kurtoglu, Alper; Tsitsilonis, Serafeim; Manegold, Sebastian; Wildemann, Britt; Klatte-Schulz, Franka | Time-Dependent Alterations of MMPs, TIMPs and Tendon Structure in Human Achilles Tendons after Acute Rupture. | International journal of molecular sciences | 3 | 2015 | 37 | 8.1 |
| 2017 | Zellers, Jennifer A.; Cortes, Daniel H.; Corrigan, Patrick; Pontiggia, Laura; Silbernagel, Karin Grävare | Side-to-side differences in Achilles tendon geometry and mechanical properties following achilles tendon rupture. | Muscles, ligaments and tendons journal | 3 | 2015 | 20 | 25.0 |
| 2018 | Nyyssönen, Timo; Lantto, Iikka; Lüthje, Peter; Selander, Tuomas; Kröger, Heikki | Drug treatments associated with Achilles tendon rupture. A case-control study involving 1118 Achilles tendon ruptures. | Scandinavian journal of medicine & science in sports | 2 | 1998 | 1118 | 21.0 |
| 2018 | Ateschrang, Atesch; Körner, Daniel; Joisten, Konrad; Ahrend, Marc-Daniel; Schröter, Steffen; Stöckle, Ulrich; Riedmann, Stephan | Incidence and risk factors for postoperative Achilles tendon calcifications after percutaneous repair. | Archives of orthopaedic and trauma surgery | 8 | 2006 | 81 | 19.8 |
| 2018 | Humbyrd, Casey Jo; Bae, Sunjae; Kucirka, Lauren M.; Segev, Dorry L. | Incidence, Risk Factors, and Treatment of Achilles Tendon Rupture in Patients With End-Stage Renal Disease. | Foot & ankle international | 15 | 2006 | 1091 | 44.5 |
| 2018 | Manegold, Sebastian; Tsitsilonis, Serafim; Schumann, Jakob; Gehlen, Tobias; Agres, Alison N.; Keller, Johannes; Gesslein, Markus; Wichlas, Florian | Functional outcome and complication rate after percutaneous suture of fresh Achilles tendon ruptures with the Dresden instrument. | Journal of orthopaedics and traumatology : official journal of the Italian Society of Orthopaedics and Traumatology | 8 | 2006 | 118 | 15.3 |
| 2018 | Lever, C. J.; Bosman, H. A.; Robinson, A. H. N. | The functional and dynamometer-tested results of transtendinous flexor hallucis longus transfer for neglected ruptures of the Achilles tendon at six years' follow-up. | The bone & joint journal | 9 | 2007 | 20 | 20.0 |
| 2018 | Maffulli, Nicola; Oliva, Francesco; Maffulli, Gayle D.; Buono, Angelo Del; Gougoulias, Nikolaos | Surgical management of chronic Achilles tendon ruptures using less invasive techniques. | Foot and ankle surgery : official journal of the European Society of Foot and Ankle Surgeons | 7 | 2007 | 62 | 37.1 |
| 2018 | Anathallee, M. Yasser; Liu, Ben; Budgen, Adam; Stanley, James | Is Achillon repair safe and reliable in delayed presentation Achilles tendon rupture? A five-year follow-up. | Foot and ankle surgery : official journal of the European Society of Foot and Ankle Surgeons | 3 | 2008 | 24 | 25.0 |
| 2018 | Pean, Christian A.; Christiano, Anthony; Rubenstein, William J.; Konda, Sanjit R.; Egol, Kenneth A. | Risk factors for complications after primary repair of Achilles tendon ruptures. | Journal of orthopaedics | 8 | 2008 | 1164 | 25.6 |
| 2018 | Frankewycz, B.; Penz, A.; Weber, J.; da Silva, N. P.; Freimoser, F.; Bell, R.; Nerlich, M.; Jung, E. M.; Docheva, D.; Pfeifer, C. G. | Achilles tendon elastic properties remain decreased in long term after rupture. | Knee surgery, sports traumatology, arthroscopy : official journal of the ESSKA | 11 | 2009 | 41 | 17.1 |
| 2018 | Alfredson, Håkan; Spang, Christoph | Clinical presentation and surgical management of chronic Achilles tendon disorders - A retrospective observation on a set of consecutive patients being operated by the same orthopedic surgeon. | Foot and ankle surgery : official journal of the European Society of Foot and Ankle Surgeons | 11 | 2010 | 12 | 41.7 |
| 2018 | Karatekin, Yavuz Selim; Karaismailoglu, Bedri; Kaynak, Gokhan; Ogut, Tahir; Dikici, Atilla Suleyman; Ure Esmerer, Emel; Aydingoz, Onder; Botanlioglu, Huseyin | Does elasticity of Achilles tendon change after suture applications? Evaluation of repair area by acoustic radiation force impulse elastography. | Journal of orthopaedic surgery and research | 9 | 2010 | 19 | 0.0 |
| 2018 | Orishimo, Karl F.; Schwartz-Balle, Sidse; Tyler, Timothy F.; McHugh, Malachy P.; Bedford, Benjamin B.; Lee, Steven J.; Nicholas, Stephen J. | Can Weakness in End-Range Plantar Flexion After Achilles Tendon Repair Be Prevented? | Orthopaedic journal of sports medicine | 9 | 2010 | 18 | 11.1 |
| 2018 | Haapasalo, Heidi; Peltoniemi, Ulla; Laine, Heikki-Jussi; Kannus, Pekka; Mattila, Ville M. | Treatment of acute Achilles tendon rupture with a standardised protocol. | Archives of orthopaedic and trauma surgery | 7 | 2011 | 411 | 19.7 |
| 2018 | Jildeh, Toufic R.; Okoroha, Kelechi R.; Marshall, Nathan E.; Abdul-Hak, Abraham; Zeni, Ferras; Moutzouros, Vasilios | Infection and Rerupture After Surgical Repair of Achilles Tendons. | Orthopaedic journal of sports medicine | 7 | 2011 | 423 | 35.2 |
| 2018 | Myhrvold, Ståle Bergman; Sandnes, Øystein; Hoelsbrekken, Sigurd Erik | Validity and reliability of the Norwegian translation of the Achilles tendon Total Rupture Score. | Knee surgery, sports traumatology, arthroscopy : official journal of the ESSKA | 4 | 2011 | 94 | 24.5 |
| 2018 | Rungprai, Chamnanni; Phisitkul, Phinit | Outcomes and Complications Following Endoscopically Assisted Percutaneous Achilles Tendon Repair. | Arthroscopy : the journal of arthroscopic & related surgery : official publication of the Arthroscopy Association of North America and the International Arthroscopy Association | 8 | 2011 | 23 | 21.7 |
| 2018 | Aujla, Randeep; Patel, Shakil; Jones, Annette; Bhatia, Maneesh | Predictors of functional outcome in non-operatively managed Achilles tendon ruptures. | Foot and ankle surgery : official journal of the European Society of Foot and Ankle Surgeons | 6 | 2012 | 236 | 15.7 |
| 2018 | Marcel, John J. Jr; Sage, Katherine; Guyton, Gregory P. | Complications of Supine Surgical Achilles Tendon Repair. | Foot & ankle international | 6 | 2012 | 45 | 13.3 |
| 2018 | Reito, A; Logren, HL; Ahonen, K; Nurmi, H; Paloneva, J | Risk Factors for Failed Nonoperative Treatment and Rerupture in Acute Achilles Tendon Rupture | FOOT & ANKLE INTERNATIONAL | 8 | 2012 | 210 | 25.2 |
| 2018 | Telleria, Jessica J. M.; Smith, Jeremy T.; Ready, Lauren V.; Bluman, Eric M. | Outcomes of Limited Open Achilles Repair Using Modified Ring Forceps. | Orthopaedic journal of sports medicine | 8 | 2012 | 32 | 15.6 |
| 2018 | Bąkowski, Paweł; Rubczak, Szymon; Wolff-Stefaniak, Maria; Grygorowicz, Monika; Piontek, Tomasz | Reliability and validity of the Polish version of the Achilles tendon Total Rupture Score. | Knee surgery, sports traumatology, arthroscopy : official journal of the ESSKA | 5 | 2013 | 71 | 0.0 |
| 2018 | Eliasson, Pernilla; Agergaard, Anne-Sofie; Couppé, Christian; Svensson, René; Hoeffner, Rikke; Warming, Susan; Warming, Nichlas; Holm, Christina; Jensen, Mikkel Holm; Krogsgaard, Michael; Kjaer, Michael; Magnusson, S. Peter | The Ruptured Achilles Tendon Elongates for 6 Months After Surgical Repair Regardless of Early or Late Weightbearing in Combination With Ankle Mobilization: A Randomized Clinical Trial. | The American journal of sports medicine | 4 | 2013 | 75 | 20.0 |
| 2018 | Hassan, A; Aziz, AMK | Flexor hallucis longus tendon transfer: a definitive procedure for Achilles tendon tear in Achilles tendinopathy. A 2-year prospective study | CURRENT ORTHOPAEDIC PRACTICE | 3 | 2013 | 22 | 22.7 |
| 2018 | Opdam, K. T. M.; Zwiers, R.; Wiegerinck, J. I.; Kleipool, A. E. B.; Haverlag, R.; Goslings, J. C.; van Dijk, C. N. | Reliability and validation of the Dutch Achilles tendon Total Rupture Score. | Knee surgery, sports traumatology, arthroscopy : official journal of the ESSKA | 3 | 2013 | 103 | 26.2 |
| 2018 | Vega, Jordi; Vilá, Jesus; Batista, Jorge; Malagelada, Francesc; Dalmau-Pastor, Miki | Endoscopic Flexor Hallucis Longus Transfer for Chronic Noninsertional Achilles Tendon Rupture. | Foot & ankle international | 4 | 2013 | 22 | 27.3 |
| 2018 | Yin, Liangjun; Wu, Yahong; Ren, Changsong; Wang, Yizhong; Fu, Ting; Cheng, Xiangjun; Li, Ruidong; Nie, Mao; Mu, Yuan | Treatment of acute achilles tendon rupture with the panda rope bridge technique. | Injury | 4 | 2013 | 11 | 0.0 |
| 2018 | Dong, Zhijun; Qiu, Bing; Pan, Yuan; Wu, Shengzhong; Hong, Xiao; Liu, Fuyao | Improved Krackow Method Combined with Unilateral Mattress Suture for Treating Recent Achilles Tendon Rupture. | Journal of the College of Physicians and Surgeons--Pakistan : JCPSP | 4 | 2014 | 19 | 5.3 |
| 2018 | Hiramatsu, Kunihiko; Tsujii, Akira; Nakamura, Norimasa; Mitsuoka, Tomoki | Ultrasonographic Evaluation of the Early Healing Process After Achilles Tendon Repair. | Orthopaedic journal of sports medicine | 5 | 2014 | 26 | 30.8 |
| 2018 | Lemme, Nicholas J.; Li, Neill Y.; DeFroda, Steven F.; Kleiner, Justin; Owens, Brett D. | Epidemiology of Achilles Tendon Ruptures in the United States: Athletic and Nonathletic Injuries From 2012 to 2016. | Orthopaedic journal of sports medicine | 5 | 2014 | 854 | 23.0 |
| 2018 | Park, Young Hwan; Chang, An Seong; Choi, Gi Won; Kim, Hak Jun | A comparison of three methods of skin closure following repair of Achilles tendon rupture. | Injury | 5 | 2014 | 122 | 13.1 |
| 2018 | Vascellari, Alberto; Spennacchio, Pietro; Combi, Alberto; Grassi, Alberto; Patella, Silvio; Bisicchia, Salvatore; Canata, Gian Luigi; Zaffagnini, Stefano | Cross-cultural adaptation and multi-centric validation of the Italian version of the Achilles tendon Total Rupture Score (ATRS). | Knee surgery, sports traumatology, arthroscopy : official journal of the ESSKA | 2 | 2014 | 80 | 7.5 |
| 2018 | van Maele, Margaux; Misselyn, Dominique; Metsemakers, Willem-Jan; Sermon, An; Nijs, Stefaan; Hoekstra, Harm | Is open acute Achilles tendon rupture repair still justified? A single center experience and critical appraisal of the literature. | Injury | 5 | 2014 | 105 | 15.2 |
| 2018 | Abubeih, Hossam; Khaled, Mohamed; Saleh, Waleed Riad; Said, Galal Z. | Flexor hallucis longus transfer clinical outcome through a single incision for chronic Achilles tendon rupture. | International orthopaedics | 2 | 2015 | 21 | 28.6 |
| 2018 | Husebye, Elisabeth Ellingsen; Molund, Marius; Hvaal, Kjetil Harald; Stødle, Are Haukåen | Endoscopic Transfer of Flexor Hallucis Longus Tendon for Chronic Achilles Tendon Rupture: Technical Aspects and Short-Time Experiences. | Foot & ankle specialist | 1 | 2015 | 6 | 16.7 |
| 2018 | Li, Qianru; Zhang, Qi; Cai, Yehua; Hua, Yinghui | Patients with Achilles Tendon Rupture Have a Degenerated Contralateral Achilles Tendon: An Elastography Study. | BioMed research international | 4 | 2015 | 33 | 18.2 |
| 2018 | MacDonald, David R. W.; Neilly, David; Littlechild, Joseph; Harrold, Fraser; Roberts, Sam C. | Acute Achilles tendon rupture: Do cast boots produce adequate equinus when used for functional rehabilitation? | Foot (Edinburgh, Scotland) | 2 | 2016 | 29 | 17.2 |
| 2019 | Čretnik, Andrej; Kosanović, Miloš; Košir, Roman | Long-Term Results With the Use of Modified Percutaneous Repair of the Ruptured Achilles Tendon Under Local Anaesthesia (15-Year Analysis With 270 Cases). | The Journal of foot and ankle surgery : official publication of the American College of Foot and Ankle Surgeons | 15 | 1998 | 267 | 7.5 |
| 2019 | Pendse, Aniruddha; Kankate, Raghubir | Reconstruction of chronic achilles tendon ruptures in elderly patients, with vascularized flexor hallucis longus tendon transfer using single incision technique. | Acta orthopaedica Belgica | 6 | 2007 | 16 | 25.0 |
| 2019 | Dombrowski, Malcolm; Murawski, Christopher D.; Yasui, Youichi; Chen, Antonia F.; Ewalefo, Samuel O.; Fourman, Mitchell S.; Kennedy, John G.; Hogan, MaCalus V. | Medical comorbidities increase the rate of surgical site infection in primary Achilles tendon repair. | Knee surgery, sports traumatology, arthroscopy : official journal of the ESSKA | 8 | 2008 | 24142 | 41.7 |
| 2019 | Khalid, Mohammed A.; Weiss, William M.; Iloanya, Michael; Panchbhavi, Vinod K. | Dual Purpose Use of Flexor Hallucis Longus Tendon for Management of Chronic Achilles Tendon Ruptures. | Foot & ankle specialist | 12 | 2010 | 10 | 50.0 |
| 2019 | Koh, Don; Lim, Jeremy; Chen, Jerry Y.; Singh, Inderjeet R.; Koo, Kevin | Flexor hallucis longus transfer versus turndown flaps augmented with flexor hallucis longus transfer in the repair of chronic Achilles tendon rupture. | Foot and ankle surgery : official journal of the European Society of Foot and Ankle Surgeons | 11 | 2010 | 49 | 46.9 |
| 2019 | Aujla, Randeep S.; Patel, Shakil; Jones, Annette; Bhatia, Maneesh | Non-operative functional treatment for acute Achilles tendon ruptures: The Leicester Achilles Management Protocol (LAMP). | Injury | 4 | 2012 | 234 | 19.2 |
| 2019 | Bai, Lu; Guan, Siyao; You, Tian; Zhang, Wentao; Chen, Peng | Comparison of Gastrocnemius Turn Flap and Hamstring Graft for the Treatment of Kuwada Type 3 Chronic Ruptures of the Achilles Tendon: A Retrospective Study. | Orthopaedic journal of sports medicine | 3 | 2013 | 26 | 3.8 |
| 2019 | Bisaccia, Michele; Rinonapoli, Giuseppe; Meccariello, Luigi; Bisaccia, Olga; Ceccarini, Paolo; Rollo, Giuseppe; Ibáñez-Vicente, Cristina; Cervera-Irimia, Javier; Sánchez-Sánchez, Felix; Ribes-Iborra, Angela; Gomez-Garrido, David; Caraffa, Auro | Validity and Reliability of Mini-Invasive Surgery Assisted by Ultrasound in Achilles Tendon Rupture. | Acta informatica medica : AIM : journal of the Society for Medical Informatics of Bosnia & Herzegovina : casopis Drustva za medicinsku informatiku BiH | 4 | 2013 | 56 | 28.6 |
| 2019 | Jiang, Xian-Jun; Shen, Jian-Jian; Huang, Jie-Feng; Tong, Pei-Jian | Reconstruction of Myerson type III chronic Achilles tendon ruptures using semitendinosus tendon and gracilis tendon autograft. | Journal of orthopaedic surgery (Hong Kong) | 4 | 2013 | 7 | 14.3 |
| 2019 | Nam, Il Hyun; Park, Young Uk; Cho, Jae Ho; Lee, Doo Hyung; Min, Kyung Jun | Comparison Between Early Functional Rehabilitation and Cast Immobilization After Minimally Invasive Repair for an Acute Achilles Tendon Rupture. | The Journal of foot and ankle surgery : official publication of the American College of Foot and Ankle Surgeons | 2 | 2013 | 41 | 14.6 |
| 2019 | Baumfeld, Daniel; Baumfeld, Tiago; Spiezia, Filippo; Nery, Caio; Zambelli, Roberto; Maffulli, Nicola | Isokinetic functional outcomes of open versus percutaneous repair following Achilles tendon tears. | Foot and ankle surgery : official journal of the European Society of Foot and Ankle Surgeons | 2 | 2014 | 38 | 7.9 |
| 2019 | Ernat, Justin J.; Johnson, John D.; Anderson, Claude D.; Ryan, Paul M.; Yim, Duke G. | Does Clinical Exam and Ultrasound Compare With MRI Findings When Assessing Tendon Approximation in Acute Achilles Tendon Tears? A Clinical Study. | The Journal of foot and ankle surgery : official publication of the American College of Foot and Ankle Surgeons | 2 | 2014 | 18 | 11.1 |
| 2019 | Park, YH; Lim, JW; Choi, GW; Kim, HJ | Quantitative Magnetic Resonance Imaging Analysis of the Common Site of Acute Achilles Tendon Rupture: 5 to 8 cm Above the Distal End of the Calcaneal Insertion | AMERICAN JOURNAL OF SPORTS MEDICINE | 8 | 2014 | 195 | 8.7 |
| 2019 | Chegini Kord, Mohammadhossein; Ebrahimpour, Adel; Sadighi, Mehrdad; Chehrassan, Mohammadreza; Nazari, Leili; Najafi, Arvin; Minator Sajjadi, Mohammadreza | Minimally Invasive Repair of Acute Achilles Tendon Rupture Using Gift Box Technique. | The archives of bone and joint surgery | 1 | 2015 | 24 | 29.2 |
| 2019 | Frantz, Travis L.; Everhart, Joshua S.; Jamieson, Marissa; Fisk, Erica; Fredrickson, Saul; Kanney, Jill; Miller, Timothy L. | Patient-Reported Outcomes of Achilles Tendon Repair Using the Modified Gift-Box Technique With Nonabsorbable Suture Loop: A Consecutive Case Series. | The Journal of foot and ankle surgery : official publication of the American College of Foot and Ankle Surgeons | 5 | 2015 | 59 | 18.6 |
| 2019 | Manent, Andrea; López, Laia; Corominas, Hèctor; Santamaría, Alejandro; Domínguez, Alejandro; Llorens, Natalia; Sales, Miquel; Videla, Sebastián | Acute Achilles Tendon Ruptures: Efficacy of Conservative and Surgical (Percutaneous, Open) Treatment-A Randomized, Controlled, Clinical Trial. | The Journal of foot and ankle surgery : official publication of the American College of Foot and Ankle Surgeons | 4 | 2015 | 34 | 8.8 |
| 2019 | Cui, Juncheng; Chen, Zhiwei; Wu, Wente | Expression of TGF-β1 and VEGF in patients with Achilles tendon rupture and the clinical efficacy. | Experimental and therapeutic medicine | 2 | 2016 | 42 | 23.8 |
| 2019 | Ko, PY; Huang, MT; Li, CL; Su, WR; Jou, IM; Wu, PT | Jigless knotless internal brace technique for acute Achilles tendon rupture: a case series study | JOURNAL OF ORTHOPAEDIC SURGERY AND RESEARCH | 3 | 2016 | 10 | 10.0 |
| 2019 | Peng, Wei-Chen; Chao, Yuan-Hung; Fu, Amy S. N.; Fong, Shirley S. M.; Rolf, Christer; Chiang, Hongsen; Chen, Shiyi; Wang, Hsing-Kuo | Muscular Morphomechanical Characteristics After an Achilles Repair. | Foot & ankle international | 3 | 2016 | 20 | 15.0 |
| 2020 | Fell, Daniel; Enocson, Anders; Lapidus, Lasse J. | Surgical repair of acute Achilles tendon ruptures: a follow-up of 639 consecutive cases. | European journal of orthopaedic surgery & traumatology : orthopedie traumatologie | 8 | 2001 | 639 | 16.0 |
| 2020 | Maempel, Julian F.; Clement, Nicholas D.; Wickramasinghe, Neil R.; Duckworth, Andrew D.; Keating, John F. | Operative repair of acute Achilles tendon rupture does not give superior patient-reported outcomes to nonoperative management. | The bone & joint journal | 5 | 2002 | 80 | 25.0 |
| 2020 | Lerch, Till D.; Schwinghammer, Andreas; Schmaranzer, Florian; Anwander, Helen; Ecker, Timo M.; Schmid, Timo; Weber, Martin; Krause, Fabian | Return to Sport and Patient Satisfaction at 5-Year Follow-up After Nonoperative Treatment for Acute Achilles Tendon Rupture. | Foot & ankle international | 17 | 2004 | 89 | 23.6 |
| 2020 | Hürmeydan, Önder Murat; Demirel, Mehmet; Valiyev, Natig; Sahinkaya, Turker; Kılıçoğlu, Önder İsmet | Relationship of Postoperative Achilles Tendon Elongation With Plantarflexion Strength Following Surgical Repair. | Foot & ankle international | 17 | 2006 | 40 | 37.5 |
| 2020 | Bilge, Ali; Kuru, Tolgahan | Results of Surgical Management of Achilles Tendon Rupture Using the Modified Lindholm Procedure. | Cureus | 8 | 2010 | 36 | 5.6 |
| 2020 | Park, Young Hwan; Kim, Tae Jin; Choi, Gi Won; Kim, Hak Jun | Age is a risk factor for contralateral tendon rupture in patients with acute Achilles tendon rupture. | Knee surgery, sports traumatology, arthroscopy : official journal of the ESSKA | 11 | 2010 | 226 | 9.3 |
| 2020 | Mutlu, T; Satilmis, AB | A novel suture technique in the percutaneous repair of Achilles tendon rupture (TANSEL TECHNIQUE) | ANNALS OF CLINICAL AND ANALYTICAL MEDICINE | 6 | 2012 | 34 | 11.8 |
| 2020 | Song, Yu-Jie; Chen, Gang; Jia, Shao-Hua; Xu, Wei-Bin; Hua, Ying-Hui | Good outcomes at mid-term following the reconstruction of chronic Achilles tendon rupture with semitendinosus allograft. | Knee surgery, sports traumatology, arthroscopy : official journal of the ESSKA | 5 | 2013 | 34 | 11.8 |
| 2020 | Ding, Haixiang; Xu, Yingshu; Li, Jie; Yue, Kai | Comparison of the curative effect of modified medial arc incision of achilles tendon and traditional straight incision in the treatment of old achilles tendon rupture. | Pakistan journal of pharmaceutical sciences | 7 | 2014 | 50 | 26.0 |
| 2020 | Maffulli, Nicola; D'Addona, Alessio; Maffulli, Gayle D.; Gougoulias, Nikolaos; Oliva, Francesco | Delayed (14-30 Days) Percutaneous Repair of Achilles Tendon Ruptures Offers Equally Good Results As Compared With Acute Repair. | The American journal of sports medicine | 4 | 2014 | 42 | 28.6 |
| 2020 | Aufwerber, Susanna; Heijne, Annette; Edman, Gunnar; Grävare Silbernagel, Karin; Ackermann, Paul W. | Early mobilization does not reduce the risk of deep venous thrombosis after Achilles tendon rupture: a randomized controlled trial. | Knee surgery, sports traumatology, arthroscopy : official journal of the ESSKA | 6 | 2015 | 149 | 22.8 |
| 2020 | Barfod, Kristoffer Weisskirchner; Hansen, Maria Swennergren; Hölmich, Per; Kristensen, Morten Tange; Troelsen, Anders | Efficacy of early controlled motion of the ankle compared with immobilisation in non-operative treatment of patients with an acute Achilles tendon rupture: an assessor-blinded, randomised controlled trial. | British journal of sports medicine | 3 | 2015 | 130 | 17.7 |
| 2020 | Carmont, Michael R.; Zellers, Jennifer A.; Brorsson, Annelie; Silbernagel, Karin Grävare; Karlsson, Jón; Nilsson-Helander, Katarina | No difference in strength and clinical outcome between early and late repair after Achilles tendon rupture. | Knee surgery, sports traumatology, arthroscopy : official journal of the ESSKA | 4 | 2015 | 18 | 5.6 |
| 2020 | Carmont, Michael R.; Zellers, Jennifer A.; Brorsson, Annelie; Nilsson-Helander, Katarina; Karlsson, Jón; Grävare Silbernagel, Karin | Age and Tightness of Repair Are Predictors of Heel-Rise Height After Achilles Tendon Rupture. | Orthopaedic journal of sports medicine | 7 | 2015 | 122 | 22.1 |
| 2020 | Liu, Jun-Yi; Duan, Wei-Feng; Shen, Sheng; Ye, Ye; Sun, Yong-Qiang; He, Wei | Achillon versus modified minimally invasive repair treatment in acute Achilles tendon rupture. | Journal of orthopaedic surgery (Hong Kong) | 5 | 2015 | 114 | 14.9 |
| 2020 | Maempel, Julian F.; Clement, Nick D.; Duckworth, Andrew D.; Keenan, Oisin J. F.; White, Tim O.; Biant, Leela C. | A Randomized Controlled Trial Comparing Traditional Plaster Cast Rehabilitation With Functional Walking Boot Rehabilitation for Acute Achilles Tendon Ruptures. | The American journal of sports medicine | 6 | 2015 | 140 | 18.6 |
| 2020 | Makulavičius, Aleksas; Mazarevičius, Giedrius; Klinga, Mindaugas; Urmanavičius, Matas; Masionis, Povilas; Oliva, Xavier Martin; Uvarovas, Valentinas; Porvaneckas, Narūnas | Outcomes of open "crown" type v. percutaneous Bunnell type repair of acute Achilles tendon ruptures. Randomized control study. | Foot and ankle surgery : official journal of the European Society of Foot and Ankle Surgeons | 5 | 2015 | 87 | 11.5 |
| 2020 | Nilsson, Niklas; Nilsson Helander, Katarina; Hamrin Senorski, Eric; Holm, Anna; Karlsson, Jón; Svensson, Mikael; Westin, Olof | The economic cost and patient-reported outcomes of chronic Achilles tendon ruptures. | Journal of experimental orthopaedics | 6 | 2015 | 40 | 27.5 |
| 2020 | Batista, JP; Abdelatif, NMN; Del Vecchio, JJ; Diniz, P; Pereira, H | Endoscopic Flexor Hallucis Longus Transfer for the Management of Acute Achilles Tendon Ruptures: A Prospective Case Series Report With a Minimum of 18 Months' Follow-Up | JOURNAL OF FOOT & ANKLE SURGERY | 4 | 2016 | 51 | 0.0 |
| 2020 | Hansen, Maria Swennergren; Kristensen, Morten Tange; Budolfsen, Thomas; Ellegaard, Karen; Hölmich, Per; Barfod, Kristoffer Weisskirchner | Reliability of the Copenhagen Achilles length measure (CALM) on patients with an Achilles tendon rupture. | Knee surgery, sports traumatology, arthroscopy : official journal of the ESSKA | 2 | 2016 | 84 | 20.2 |
| 2020 | Holzgrefe, Russell E.; McCarthy, Timothy P.; Wilson, Jacob M.; Bariteau, Jason T.; Labib, Sameh | Association of Strength Following Achilles Tendon Repair With Return to Same Level of Play in High-Level Athletes. | Foot & ankle international | 5 | 2016 | 36 | 27.8 |
| 2020 | Joannas, German; Arrondo, Guillermo; Eslava, Santiago; Casola, Leandro; Drago, Juan; Barousse, Rafael; Niño Gomez, Daniel; Amlang, Michael; Rammelt, Stefan | Percutaneous Achilles tendon repair with the Dresden instrument. Clinical and MRI evaluation of 90 patients. | Foot and ankle surgery : official journal of the European Society of Foot and Ankle Surgeons | 3 | 2016 | 90 | 3.3 |
| 2020 | Lee, Jeong-Kil; Kang, Chan; Hwang, Deuk-Soo; Kang, Dong-Hun; Lee, Gi-Soo; Hwang, Jung-Mo; Song, Jae-Hwang; Lee, Cheol-Won | A comparative study of innovative percutaneous repair and open repair for acute Achilles tendon rupture: Innovative usage of intraoperative ultrasonography. | Journal of orthopaedic surgery (Hong Kong) | 3 | 2016 | 30 | 10.0 |
| 2020 | Reb, Christopher W.; McDonald, Elizabeth; Shakked, Rachel J.; Winters, Brian S.; Pedowitz, David I.; Raikin, Steven M.; Daniel, Joseph N. | Brake Response Time Recovery After Achilles Tendon Repair. | Foot & ankle specialist | 3 | 2016 | 59 | 20.3 |
| 2020 | Yang, YL; Jia, HL; Zhang, WP; Xu, SH; Wang, F; Wang, BM; Li, QH; Wang, YH; Han, SM | Intraoperative ultrasonography assistance for minimally invasive repair of the acute Achilles tendon rupture | JOURNAL OF ORTHOPAEDIC SURGERY AND RESEARCH | 3 | 2016 | 36 | 22.2 |
| 2020 | Buckinx, F.; Lecoq, G.; Bornheim, S.; Van Beveren, J.; Valcu, A.; Daniel, C.; Bruyère, O.; Reginster, J. Y.; D'Hooghe, P.; Kaux, J. F. | French translation and validation of the Achilles Tendon Total Rupture Score "ATRS". | Foot and ankle surgery : official journal of the European Society of Foot and Ankle Surgeons | 2 | 2017 | 44 | 27.3 |
| 2020 | Costa, Matthew L.; Achten, Juul; Marian, Ioana R.; Dutton, Susan J.; Lamb, Sarah E.; Ollivere, Benjamin; Maredza, Mandy; Petrou, Stavros; Kearney, Rebecca S. | Plaster cast versus functional brace for non-surgical treatment of Achilles tendon rupture (UKSTAR): a multicentre randomised controlled trial and economic evaluation. | Lancet (London, England) | 3 | 2017 | 538 | 20.8 |
| 2020 | Frankewycz, Borys; Henssler, Leopold; Weber, Johannes; Silva, Natascha Platz Batista da; Koch, Matthias; Jung, Ernst Michael; Docheva, Denitsa; Alt, Volker; Pfeifer, Christian G. | Changes of Material Elastic Properties during Healing of Ruptured Achilles Tendons Measured with Shear Wave Elastography: A Pilot Study. | International journal of molecular sciences | 3 | 2017 | 12 | 8.3 |
| 2020 | Mubark, Islam; Abouelela, Amr; Arya, Swati; Buchanan, Donald; Elgalli, Mosab; Parker, Jennifer; Ashwood, Neil; Karagkevrekis, Charalampos | Achilles Tendon Rupture: Can the Tendon Gap on Ultrasound Scan Predict the Outcome of Functional Rehabilitation Program? | Cureus | 3 | 2017 | 56 | 37.5 |
| 2020 | Wang, Xiaomeng; Liu, Huixin; Li, Dengke; Luo, Zixuan; Li, Yansen; Zhang, Fengqi | Modified Bunnell suture repair versus bundle-to-bundle suture repair for acute Achilles tendon rupture: a prospective comparative study of patients aged <45 years. | BMC musculoskeletal disorders | 3 | 2017 | 61 | 8.2 |
| 2020 | Yang, Yu-Ping; Tao, Li-Yuan; Gao, Jia-Ning; Wang, Peng; Jiang, Yan-Fang; Zheng, Le-Min; Zhao, Yi-Ming; Ao, Ying-Fang | Elevated lipid levels in patients with achilles tendon ruptures: a retrospective matching study. | Annals of translational medicine | 1 | 2017 | 241 | 9.1 |
| 2020 | Yassin, Mohamed; Myatt, Richard; Thomas, William; Gupta, Vatsal; Hoque, Tagrit; Mahadevan, Devendra | Does size of tendon gap affect patient-reported outcome following Achilles tendon rupture treated with functional rehabilitation? | The bone & joint journal | 4 | 2017 | 82 | 25.6 |
| 2020 | Cho, Jaeho; Kim, Hyun-Joo; Lee, Jeong Seok; Kim, Jahyung; Won, Sung Hun; Yi, Young; Chun, Dong-Il | Comparing Absorbable and Nonabsorbable Suture Materials for Repair of Achilles Tendon Rupture: A Magnetic Resonance Imaging-Based Study. | Diagnostics (Basel, Switzerland) | 3 | 2018 | 22 | 18.2 |
| 2020 | Dams, Olivier C.; Reininga, Inge H. F.; Zwerver, Johannes; Diercks, Ronald L.; van den Akker-Scheek, Inge | The Achilles tendon Total Rupture Score is a responsive primary outcome measure: an evaluation of the Dutch version including minimally important change. | Knee surgery, sports traumatology, arthroscopy : official journal of the ESSKA | 3 | 2018 | 47 | 34.0 |
| 2020 | Illeu, M; Milosevic, I; Ilic, M; Matic, S; Tabakovic, D; Elbors, D; Parapid, B; Lugonja, S | Prognostic model of clinical scores in evaluation of treatment outcome in patients with acute Achilles tendon rupture - surgery vs. immobilization | SRPSKI ARHIV ZA CELOKUPNO LEKARSTVO | 3 | 2018 | 80 | 31.3 |
| 2020 | Okoroha, KR; Ussef, N; Jildeh, TR; Khalil, LS; Hasan, L; Bench, C; Zeni, F; Eller, E; Moutzouros, V | Comparison of Tendon Lengthening With Traditional Versus Accelerated Rehabilitation After Achilles Tendon Repair: A Prospective Randomized Controlled Trial | AMERICAN JOURNAL OF SPORTS MEDICINE | 3 | 2018 | 18 | 11.1 |
| 2021 | Sanchez, Rafael; Hodgens, Blake H.; Geller, Joseph S.; Huntley, Samuel; Kaplan, Jonathan; Aiyer, Amiethab | Effect of Achilles Tendon Repair on Performance Outcomes After Return to Play in National Collegiate Athletic Association Division I Basketball Athletes. | Orthopaedic journal of sports medicine | 20 | 2009 | 75 | 36.0 |
| 2021 | Saxena, Amol; Maffulli, Nicola; Jin, Anqi; Isa, Eghosa; Arthur, William Philip; Asthana, Saumya | Acute Achilles Tendon Rupture Repair in Athletically Active Patients: Results on 188 Tendons. | The Journal of foot and ankle surgery : official publication of the American College of Foot and Ankle Surgeons | 18 | 2009 | 186 | 21.0 |
| 2021 | Paczesny, L; Zabrzynski, J; Domzalski, M; Gagat, M; Termanowski, M; Szwedowski, D; Lapaj, L; Kruczynski, J | Mini-Invasive, Ultrasound Guided Repair of the Achilles Tendon Rupture-A Pilot Study | JOURNAL OF CLINICAL MEDICINE | 11 | 2010 | 35 | 20.0 |
| 2021 | Winson, Daniel M. G.; MacNair, Rory; Hutchinson, Anne-Marie; Owen, Nick J.; Evans, Rhodri; Williams, Paul | Delayed Achilles tendon rupture presentation: Non-operative management may be the SMART choice. | Foot (Edinburgh, Scotland) | 7 | 2011 | 19 | 15.8 |
| 2021 | Biz, Carlo; Cerchiaro, Mariachiara; Belluzzi, Elisa; Bragazzi, Nicola Luigi; De Guttry, Giacomo; Ruggieri, Pietro | Long Term Clinical-Functional and Ultrasound Outcomes in Recreational Athletes after Achilles Tendon Rupture: Ma and Griffith versus Tenolig. | Medicina (Kaunas, Lithuania) | 5 | 2012 | 90 | 17.8 |
| 2021 | Eken, Gökay; Misir, Abdulhamit; Tangay, Cem; Atici, Teoman; Demirhan, Nevzat; Sener, Nadir | Effect of muscle atrophy and fatty infiltration on mid-term clinical, and functional outcomes after Achilles tendon repair. | Foot and ankle surgery : official journal of the European Society of Foot and Ankle Surgeons | 13 | 2012 | 46 | 4.3 |
| 2021 | Gunaratne, R; Chong, YC; Heng, Y; Hahn, J; Lek, J; Randazzo, A; Brankov, B | Chronic Achilles tendon rupture: a novel modification of surgical technique described by El Shewy | ANZ JOURNAL OF SURGERY | 11 | 2012 | 13 | 15.4 |
| 2021 | Li, Yang; Jiang, Yiqiu; Tao, Tianqi; Pan, Zhu; Zhang, Kaibin; Gui, Jianchao | Endoscopic reconstruction for chronic Achilles tendon ruptures using a hamstring tendon autograft. | Journal of orthopaedic science : official journal of the Japanese Orthopaedic Association | 6 | 2012 | 26 | 15.4 |
| 2021 | Fischer, Sebastian; Colcuc, Christin; Gramlich, Yves; Stein, Thomas; Abdulazim, Ahmed; von Welck, Stephanie; Hoffmann, Reinhard | Prospective randomized clinical trial of open operative, minimally invasive and conservative treatments of acute Achilles tendon tear. | Archives of orthopaedic and trauma surgery | 4 | 2013 | 90 | 10.0 |
| 2021 | Gillissen, Stijn; Halperin, Ilan; Balesar, Vinay; Gorter, Erwin; Greeven, Alexander | Functional and early weight-bearing protocol for achilles tendon ruptures: a retrospective study. | European journal of trauma and emergency surgery : official publication of the European Trauma Society | 12 | 2013 | 304 | 21.7 |
| 2021 | Yamaguchi, Satoshi; Kimura, Seiji; Akagi, Ryuichiro; Yoshimura, Kensuke; Kawasaki, Yohei; Shiko, Yuki; Sasho, Takahisa; Ohtori, Seiji | Increase in Achilles Tendon Rupture Surgery in Japan: Results From a Nationwide Health Care Database. | Orthopaedic journal of sports medicine | 8 | 2013 | 112601 | 33.1 |
| 2021 | Akoh, Craig C.; Fletcher, Amanda; Sharma, Akhil; Parekh, Selene G. | Clinical Outcomes and Complications Following Limited Open Achilles Repair Without an Instrumented Guide. | Foot & ankle international | 9 | 2014 | 33 | 21.2 |
| 2021 | Jiang, Xiang; Qian, Shenglong; Chen, Cheng; Wu, Helin; Zhi, Xiaosong; Xu, Dan; Lian, Junhong; Liu, Ximing; Wei, Shijun; Xu, Feng | Modified mini-incision "internal splinting" versus percutaneous repair technique of acute Achilles tendon rupture: five year retrospective case-controlled study. | International orthopaedics | 5 | 2014 | 53 | 7.5 |
| 2021 | McKissack, Haley; McLynn, Ryan; Pitts, Charles; Alexander, Bradley; Jones, James; Andrews, Nicholas A.; Littlefield, Zachary L.; Shah, Ashish | Safety and Efficacy of Achilles Repair Using the Mini-Open Approach in Supine Versus Prone Position: A Retrospective Study. | Cureus | 8 | 2014 | 80 | 16.3 |
| 2021 | Röell, Anna E.; Timmers, Tim K.; van der Ven, Denise J. C.; van Olden, Ger D. J. | Rehabilitation After Surgical Repair of Acute Achilles Tendon Rupture: Functional Outcome With a Minimum Follow-Up of 6 Months. | The Journal of foot and ankle surgery : official publication of the American College of Foot and Ankle Surgeons | 2 | 2014 | 32 | 15.6 |
| 2021 | Tsukada, Keisuke; Yasui, Youichi; Kubo, Maya; Miki, Shinya; Matsui, Kentaro; Sasahara, Jun; Kawano, Hirotaka; Miyamoto, Wataru | Operative Outcome of Side-Locking Loop Suture Technique Accompanied by Autologous Semitendinosus Tendon Grafting for Chronic Rupture of Achilles Tendon. | Foot & ankle orthopaedics | 6 | 2014 | 10 | 40.0 |
| 2021 | Cramer, Allan; Jacobsen, Nanna Cecilie; Hansen, Maria Swennergren; Sandholdt, Håkon; Hölmich, Per; Barfod, Kristoffer Weisskirchner | Diabetes and treatment with orally administrated corticosteroids negatively affect treatment outcome at follow-up after acute Achilles tendon rupture. | Knee surgery, sports traumatology, arthroscopy : official journal of the ESSKA | 8 | 2015 | 2004 | 20.6 |
| 2021 | Ma, Yanming; Meng, Xiangjun; Su, Yun; Yan, Zuofa; Shao, Quansheng; Chen, Yiqing | Evaluation of a Modified Spoon-Shaped Medial Incision in the Surgical Repair of a Chronic Achilles Tendon Rupture. | The Journal of foot and ankle surgery : official publication of the American College of Foot and Ankle Surgeons | 5 | 2015 | 50 | 42.0 |
| 2021 | Park, Chul Hyun; Yan, Hongfei; Park, Jeongjin; Chang, Min Cheol | Mini-open Repair for Acute Achilles Tendon Rupture: Ring Forceps vs the Achillon Device. | The American journal of sports medicine | 8 | 2015 | 50 | 18.0 |
| 2021 | Peterson, Joshua G.; Tjong, Vehniah K.; Mehta, Mitesh P.; Goyette, Bailey N.; Patel, Milap; Kadakia, Anish R. | A qualitative assessment of return to sport following Achilles tendon repair. | Journal of orthopaedics | 6 | 2015 | 23 | 17.4 |
| 2021 | Yassin, Mohamed; Gupta, Vatsal; Martins, Andre; Mahadevan, Devendra; Bhatia, Maneesh | Patient reported outcomes and satisfaction following single incision Flexor Hallucis Longus (FHL) augmentation for chronic Achilles tendon pathologies. | Journal of clinical orthopaedics and trauma | 11 | 2015 | 24 | 12.5 |
| 2021 | Zellers, Jennifer A.; Baxter, Josh R.; Grävare Silbernagel, Karin | Functional Ankle Range of Motion but Not Peak Achilles Tendon Force Diminished With Heel-Rise and Jumping Tasks After Achilles Tendon Repair. | The American journal of sports medicine | 3 | 2015 | 11 | 9.1 |
| 2021 | Zou, Yunxuan; Li, Xue; Wang, Lei; Tan, Caixia; Zhu, Yongzhan | Endoscopically Assisted, Minimally Invasive Reconstruction for Chronic Achilles Tendon Rupture With a Double-Bundle Flexor Hallucis Longus. | Orthopaedic journal of sports medicine | 2 | 2015 | 19 | 10.5 |
| 2021 | Maffulli, Nicola; Oliva, Francesco; Migliorini, Filippo | Check-rein technique for Achilles tendon elongation following conservative management for acute Achilles tendon ruptures: a two-year prospective clinical study. | Journal of orthopaedic surgery and research | 7 | 2016 | 43 | 23.3 |
| 2021 | Park, Chul Hyun; Na, Ho Dong; Chang, Min Cheol | Clinical Outcomes of Minimally Invasive Repair Using Ring Forceps for Acute Achilles Tendon Rupture. | The Journal of foot and ankle surgery : official publication of the American College of Foot and Ankle Surgeons | 5 | 2016 | 26 | 15.4 |
| 2021 | Prejbeanu, Radu; Vermesan, Dinu; Balanescu, Andrei; Mioc, Mihail Lazar; Haragus, Horia | Repair of acute Achilles tears with plantaris augmentation. | International orthopaedics | 5 | 2016 | 46 | 19.6 |
| 2021 | Bian, DL; Wang, XM; Huang, K; Zhang, Z | Evaluation of Functional Recovery Following Achilles Tendon Ruptures by Shear Wave Elastography: A Before-and-After Study | IRANIAN JOURNAL OF RADIOLOGY | 3 | 2017 | 35 | 0.0 |
| 2021 | Caolo, Kristin C.; Eble, Stephanie K.; Rider, Carson; Elliott, Andrew J.; Demetracopoulos, Constantine A.; Deland, Jonathan T.; Drakos, Mark C.; Ellis, Scott J. | Clinical Outcomes and Complications With Open vs Minimally Invasive Achilles Tendon Repair. | Foot & ankle orthopaedics | 4 | 2017 | 185 | 19.5 |
| 2021 | Gamal, O; Shams, A; Mesregah, MK | Augmented Repair of Acute Total Achilles Tendon Rupture With Peroneus Brevis Tendon Transfer Using Oblique Transosseous Calcaneal Tunnel: A Prospective Case Series | JOURNAL OF FOOT & ANKLE SURGERY | 3 | 2017 | 42 | 21.4 |
| 2021 | Li, Y; Jiang, Q; Chen, H; Xin, HK; He, Q; Ruan, DK | Comparison of mini-open repair system and percutaneous repair for acute Achilles tendon rupture | BMC MUSCULOSKELETAL DISORDERS | 3 | 2017 | 68 | 8.8 |
| 2021 | Slagers, Anton J.; Dams, Olivier C.; van Zalinge, Sara D.; Geertzen, Jan Hb; Zwerver, Johannes; Reininga, Inge Hf; van den Akker-Scheek, Inge | Psychological Factors Change During the Rehabilitation of an Achilles Tendon Rupture: A Multicenter Prospective Cohort Study. | Physical therapy | 2 | 2017 | 50 | 32.0 |
| 2021 | Braccagni, Marc; Grange, Sylvain; Arcade, Aubin; Klasan, Antonio; Boyer, Bertrand; Farizon, Frederic; Philippot, Remi; Neri, Thomas | What Are the Sonographic Outcomes of Acute Achilles Tendon Rupture? Nonoperative Versus Surgical Repair? | Surgical technology international | 2 | 2018 | 30 | 16.7 |
| 2021 | Carmont, Michael R.; Brorsson, Annelie; Barfod, Kristoffer Weisskirchner; Ginder, Laurence; Littlehales, Julie; Karlsson, Jón; Nilsson-Helander, Katarina | The reliability, reproducibility and utilization of the radiographic Achilles Tendon Loading Angle in the management of Achilles Tendon rupture. | Foot and ankle surgery : official journal of the European Society of Foot and Ankle Surgeons | 3 | 2018 | 18 | 22.2 |
| 2021 | Park, Young Hwan; Cho, Hyun Woo; Choi, Jung Woo; Kim, Hak Jun | Validation and cross-cultural adaptation of the Korean translation of the Achilles tendon Total Rupture Score. | BMC musculoskeletal disorders | 3 | 2018 | 38 | 15.8 |
| 2021 | Wei, SJ; Chen, J; Kong, CW; Xu, F; Zhi, XS; Cai, XH | Endoscopic "internal splinting" repair technique for acute Achilles tendon rupture | ARCHIVES OF ORTHOPAEDIC AND TRAUMA SURGERY | 2 | 2018 | 23 | 4.3 |
| 2022 | Leino, Oskari; Keskinen, Heli; Laaksonen, Inari; Mäkelä, Keijo; Löyttyniemi, Eliisa; Ekman, Elina | Incidence and Treatment Trends of Achilles Tendon Ruptures in Finland: A Nationwide Study. | Orthopaedic journal of sports medicine | 23 | 2008 | 30162 | 24.6 |
| 2022 | Oliva, Francesco; Marsilio, Emanuela; Asparago, Giovanni; Giai Via, Alessio; Biz, Carlo; Padulo, Johnny; Spoliti, Marco; Foti, Calogero; Oliva, Gabriella; Mannarini, Stefania; Rossi, Alessandro Alberto; Ruggieri, Pietro; Maffulli, Nicola | Achilles Tendon Rupture and Dysmetabolic Diseases: A Multicentric, Epidemiologic Study. | Journal of clinical medicine | 19 | 2012 | 340 | 17.4 |
| 2022 | Sikorski, Łukasz; Czamara, Andrzej | Retrospective Study from a Single Center in Poland of Postoperative Outcomes of Muscle Strength in Patients After Surgical Suturing of the Achilles Tendon Using a Kessler's Suture and 28 Weeks of Supervised Postoperative Physiotherapy. | Medical science monitor : international medical journal of experimental and clinical research | 12 | 2013 | 20 | 0.0 |
| 2022 | Ahn, Jungtae; Jeong, Bi O. | Return to Sports Activities After Flexor Hallucis Longus Transfer for Neglected Achilles Tendon Rupture. | The Journal of foot and ankle surgery : official publication of the American College of Foot and Ankle Surgeons | 10 | 2014 | 28 | 39.3 |
| 2022 | Sanada, Takaki; Iwaso, Hiroshi; Fukai, Atsushi; Honda, Eisaburo; Yoshitomi, Hiroki | Comparison Study of Mini-Incision Versus Original Open Technique of the Half-Mini-Bunnell Achilles Tendon Repair. | The Journal of foot and ankle surgery : official publication of the American College of Foot and Ankle Surgeons | 2 | 2014 | 143 | 34.3 |
| 2022 | Carmont, Michael R.; Knutsson, Sara Brandt; Brorsson, Annelie; Karlsson, Jón; Nilsson-Helander, Katarina | The release of adhesions improves outcome following minimally invasive repair of Achilles tendon rupture. | Knee surgery, sports traumatology, arthroscopy : official journal of the ESSKA | 13 | 2015 | 14 | 14.3 |
| 2022 | Chen, Daniel L.; Beran, Matthew C.; Duncan, Molly; Young, Julie A.; Napolitano, Jonathan T.; MacDonald, James | Achilles Tendon Injuries Requiring Surgical Treatment in the Pediatric and Adolescent Population: A Case Series. | Current sports medicine reports | 11 | 2015 | 8 | 50.0 |
| 2022 | Dai, Gaole; Zheng, Yijing; Lu, Xiaolang; Liu, Yang; Weng, Qihao; Hong, Jianjun | The percutaneous oval forceps suture-guiding method with anchor nails for Achilles tendon repair. | Injury | 4 | 2015 | 32 | 15.6 |
| 2022 | Maffulli, Nicola; Gougoulias, Nikolaos; Maffulli, Gayle D.; Oliva, Francesco; Migliorini, Filippo | Slowed-Down Rehabilitation Following Percutaneous Repair of Achilles Tendon Rupture. | Foot & ankle international | 4 | 2015 | 60 | 28.3 |
| 2022 | Myhrvold, Ståle B.; Brouwer, Espen F.; Andresen, Tor K. M.; Rydevik, Karin; Amundsen, Madeleine; Grün, Wolfram; Butt, Faisal; Valberg, Morten; Ulstein, Svend; Hoelsbrekken, Sigurd E. | Nonoperative or Surgical Treatment of Acute Achilles' Tendon Rupture. | The New England journal of medicine | 6 | 2015 | 526 | 25.7 |
| 2022 | Won Lee, Ki; Bae, Joo-Yul; Ho, Byeong Cheol; Kim, Joon Hee; Seo, Dong-Kyo | Immediate Weightbearing and Ankle Motion Exercise After Acute Achilles Tendon Rupture Repair. | The Journal of foot and ankle surgery : official publication of the American College of Foot and Ankle Surgeons | 3 | 2015 | 56 | 37.5 |
| 2022 | Hoskins, Tyler; Patel, Jay; Choi, Joseph H.; Fitzpatrick, Brendan; Begley, Brian; Mazzei, Chris J.; Harrington, Colin J.; Miller, Justin M.; Wittig, James C.; Epstein, David | Mini-Open Achilles Tendon Repair: Improving Outcomes While Decreasing Complications. | Foot & ankle specialist | 8 | 2016 | 103 | 14.6 |
| 2022 | Hung, Chun-Yu; Lin, Shih-Jie; Yeh, Chia-Yi; Yeh, Wen-Ling | Effect of Platelet-Rich Plasma Augmentation on Endoscopy-Assisted Percutaneous Achilles Tendon Repair. | Journal of clinical medicine | 5 | 2016 | 62 | 4.8 |
| 2022 | Keene, DJ; Alsousou, J; Harrison, P; O'Connor, HM; Wagland, S; Dutton, SJ; Hulley, P; Lamb, SE; Willett, K; PATH 2 Trial Grp | Platelet- rich plasma injection for acute Achilles tendon rupture | BONE & JOINT JOURNAL | 3 | 2016 | 228 | 24.6 |
| 2022 | Li, J; Yu, H; Zhan, JF; Zhang, JS; Xu, XZ; Yao, YF; Tian, DS; Xie, J | Comparison of Ma-Griffith combined with a minimally invasive small incision to a modified suture technique for the treatment of acute achilles tendon ruptures | BMC MUSCULOSKELETAL DISORDERS | 9 | 2016 | 67 | 3.0 |
| 2022 | Teng, Zhao-Lin; Cao, Sheng-Xuan; Ma, Xin; Wang, Xu; Huang, Jia-Zhang; Zhang, Chao; Geng, Xiang | Epidemiological Characteristics of Patients Operated for Achilles Tendon Rupture in Shanghai. | Orthopaedic surgery | 8 | 2016 | 293 | 8.9 |
| 2022 | Yu, Hao; Wang, Fangyuan; Xie, Jia; Yao, Yunfeng; Jing, Juehua; Li, Jun | The Reinforced Ma-Griffith Method Combined with Minimally Invasive Small-Incision Suture for Acute Achilles Tendon Rupture. | Orthopaedic surgery | 9 | 2016 | 31 | 0.0 |
| 2022 | Abdelatif, Nasef Mohamed N.; Batista, Jorge Pablo | Outcomes of Percutaneous Achilles Repair Compared With Endoscopic Flexor Hallucis Longus Tendon Transfer to Treat Achilles Tendon Ruptures. | Foot & ankle international | 3 | 2017 | 117 | 6.0 |
| 2022 | Kokulu, Kamil; Altunok, İbrahim; Sert, Ekrem Taha; Özdemir, Serdar; Mutlu, Hüseyin; Akça, Hatice Şeyma | Diagnostic Value of Lateral Ankle Radiography in Achilles Tendon Rupture. | Foot & ankle specialist | 5 | 2017 | 154 | 12.3 |
| 2022 | Larsson, Elin; Brorsson, Annelie; Carling, Malin; Johansson, Christer; Carmont, Michael R.; Nilsson Helander, Katarina | Sex differences in patients' recovery following an acute Achilles tendon rupture - a large cohort study. | BMC musculoskeletal disorders | 6 | 2017 | 564 | 22.9 |
| 2022 | Samy, Ahmed Mohamed | Intra-operative ultrasound: does it improve the results of percutaneous repair of acute Achilles tendon rupture? | European journal of trauma and emergency surgery : official publication of the European Trauma Society | 7 | 2017 | 91 | 20.9 |
| 2022 | Tang, Kaiying; Deng, Zhibo; Wang, Ting; Sun, Xianding; Yin, Liangjun; Nie, Mao | Panda rope bridge technique versus open repair of acute Achilles tendon rupture: A comparative clinical study. | Injury | 5 | 2017 | 98 | 12.2 |
| 2022 | Haroun, Haitham Kamel; Abd Elrahman, Amr Ahmed; Morsi, Ahmed | Synthetic Graft Augmentation Is Safe and Effective for the Repair of Acute Achilles Tendon Rupture in Patients With Preexisting Tendinopathy. | Arthroscopy, sports medicine, and rehabilitation | 3 | 2018 | 13 | 38.5 |
| 2022 | Idarraga, Alexander J.; Bohl, Daniel D.; Barnard, Eric; Movassaghi, Kamran; Hamid, Kamran S.; Schiff, Adam P. | Adverse Events Following Minimally Invasive Achilles Tendon Repair. | Foot & ankle specialist | 3 | 2018 | 99 | 14.1 |
| 2022 | Larsson, Elin; Helander, Katarina Nilsson; Falkheden Henning, Lotta; Heiskanen, Mervi; Carmont, Michael R.; Grävare Silbernagel, Karin; Brorsson, Annelie | Achilles tendon resting angle is able to detect deficits after an Achilles tendon rupture, but it is not a surrogate for direct measurements of tendon elongation, function or symptoms. | Knee surgery, sports traumatology, arthroscopy : official journal of the ESSKA | 5 | 2018 | 60 | 21.7 |
| 2022 | Nguyen, Luong Van; Nguyen, Gioi Nang; Nguyen, Binh Lam | The modified mini-open technique for repairing total ruptured Achilles tendon using fiber wire with calcaneal fixation. A prospective case series. | Annals of medicine and surgery (2012) | 4 | 2018 | 21 | 19.0 |
| 2022 | Anil, R; Prabhu, A; Kumar, N | Management of Closed Tendoachilles | JOURNAL OF CLINICAL AND DIAGNOSTIC RESEARCH | 3 | 2019 | 11 | 18.2 |
| 2022 | Bansal, N; Dev, P; Tiwari, P; Jain, A | Clinical Evaluation of a Minimally Invasive Technique Using a Free Semitendinosus Tendon Graft for Reconstruction of a Chronic Achilles Tendon Tear With Wide Gap | TECHNIQUES IN ORTHOPAEDICS | 1 | 2019 | 10 | 30.0 |
| 2022 | Naskar, R.; Oliver, L.; Velazquez-Ruta, P.; Dhinsa, B.; Southgate, C. | Functional outcome of early weight bearing for acute Achilles tendon rupture treated conservatively in a weight-bearing orthosis. | Foot and ankle surgery : official journal of the European Society of Foot and Ankle Surgeons | 3 | 2019 | 39 | 17.9 |
| 2022 | Park, Young H.; Kim, Woon; Choi, Jung W.; Kim, Hak J. | Absorbable versus nonabsorbable sutures for the Krackow suture repair of acute Achilles tendon rupture: a prospective randomized controlled trial. | The bone & joint journal | 3 | 2019 | 36 | 13.9 |
| 2022 | Cramer, Allan; Moser, Claus; Fritz, Blaine Gabriel; Hölmich, Per; Barfod, Kristoffer Weisskirchner | Involvement of Bacteria in the Pathological Changes Before Achilles Tendon Rupture: A Case Series Investigating 16S rDNA in 20 Consecutive Ruptures. | Orthopaedic journal of sports medicine | 3 | 2020 | 20 | 30.0 |
| 2022 | Cramer, Allan; Ingelsrud, Lina Holm; Hansen, Maria Swennergren; Hölmich, Per; Barfod, Kristoffer Weisskirchner | Estimation of Patient Acceptable Symptom State (PASS) and Treatment Failure (TF) Threshold Values for the Achilles Tendon Total Rupture Score (ATRS) at 6 Months, 1 Year, and 2 Years After Acute Achilles Tendon Rupture. | The Journal of foot and ankle surgery : official publication of the American College of Foot and Ankle Surgeons | 2 | 2020 | 287 | 18.5 |
| 2023 | Tarczyńska, Marta; Szubstarski, Mateusz; Gawęda, Krzysztof; Przybylski, Piotr; Czekajska-Chehab, Elżbieta | Outcomes of Open Repair Treatment for Acute Versus Chronic Achilles Tendon Ruptures: Long-Term Retrospective Follow-Up of a Minimum 10 Years-A Pilot Study. | Medical sciences (Basel, Switzerland) | 16 | 2003 | 30 | 30.0 |
| 2023 | Čretnik, Andrej; Košir, Roman | Incidence of Achilles tendon rupture: 25-year regional analysis with a focus on bilateral ruptures. | The Journal of international medical research | 25 | 2003 | 524 | 7.3 |
| 2023 | Bishop, Meghan E.; Comer, Carly D.; Kane, Justin M.; Maltenfort, Mitchell G.; Raikin, Steven M. | Republication of "Open Repair of Acute Achilles Tendon Ruptures: Is the Incidence of Clinically Significant Wound Complications Overestimated?". | Foot & ankle orthopaedics | 15 | 2007 | 369 | 14.9 |
| 2023 | Suphinnapong, Pawichaya; Teeranon, Niphon; Teerakidpisan, Sikorn; Tansuthunluck, Sora; Apinun, Jirun | Validity and reliability of the Thai version of the Achilles tendon total rupture score. | Knee surgery, sports traumatology, arthroscopy : official journal of the ESSKA | 15 | 2010 | 50 | 28.0 |
| 2023 | Trivedi, Nikunj N.; Varshneya, Kunal; Calcei, J. Blake; Lin, Kenneth; Sochaki, Kyle R.; Voos, James E.; Safran, Marc R.; Calcei, Jacob G. | Achilles Tendon Repairs: Identification of Risk Factors for and Economic Impact of Complications and Reoperation. | Sports health | 10 | 2011 | 50279 | 32.1 |
| 2023 | Ahn, Hyeong Sik; Kim, Hyun Jung; Suh, Jin Soo; Kazmi, Sayada Zartasha; Kang, Tae Uk; Choi, Jun Young | The Association of Body Mass Index and Waist Circumference with the Risk of Achilles Tendon Problems: A Nationwide Population-Based Longitudinal Cohort Study. | Clinics in orthopedic surgery | 9 | 2013 | 31405 | 26.3 |
| 2023 | Jiménez-Carrasco, Cristina; Ammari-Sánchez-Villanueva, Fadi; Prada-Chamorro, Estefanía; García-Guirao, Antonio Jesús; Tejero, Sergio | Allograft and Autologous Reconstruction Techniques for Neglected Achilles Tendon Rupture: A Mid-Long-Term Follow-Up Analysis. | Journal of clinical medicine | 12 | 2013 | 17 | 17.6 |
| 2023 | Kosiol, Juana; Keiler, Alexander; Loizides, Alexander; Gruber, Hannes; Henninger, Benjamin; Bölderl, Andreas; Gruber, Leonhard | Operative versus conservative treatment of acute Achilles tendon ruptures: preliminary results of clinical outcome, kinematic MRI and contrast-enhanced ultrasound. | Archives of orthopaedic and trauma surgery | 8 | 2013 | 18 | 0.0 |
| 2023 | Stake, Ingrid K.; Matheny, Lauren M.; Comfort, Spencer M.; Dornan, Grant J.; Haytmanek, C. Thomas; Clanton, Thomas O. | Outcomes Following Repair of Achilles Midsubstance Tears: Percutaneous Knotless Repair vs Open Repair. | Foot & ankle international | 10 | 2013 | 63 | 20.6 |
| 2023 | Hong, Jee Young; Kang, Chan; Kim, Tae Gyun; Yi, Jin Woong; Song, Jae Hwang; Lee, Gi Soo; Seo, Kyung Deok; Shin, Woo Jin; Jo, Seong Kyeong | Risk Factors for Contralateral Tendon Rupture in Patients With Acute Achilles Tendon Rupture. | The Journal of foot and ankle surgery : official publication of the American College of Foot and Ankle Surgeons | 9 | 2014 | 181 | 15.5 |
| 2023 | Xu, Yangbo; Li, Cui; Liu, Tianyu; Xiang, Feifan; Deng, Yong; Li, Zhong; Wei, Daiqing | Long-term outcome of flexor hallucis longus tendon transfer for chronic Achilles tendon rupture with large defect: A retrospective series. | Medicine | 11 | 2014 | 28 | 17.9 |
| 2023 | Murdock, Christopher J.; Ochuba, Arinze J.; Xu, Amy L.; Snow, Morgan; Bronheim, Rachel; Vulcano, Ettore; Aiyer, Amiethab A. | Operative vs Nonoperative Management of Achilles Tendon Rupture: A Cost Analysis. | Foot & ankle orthopaedics | 11 | 2015 | 100825 | 39.3 |
| 2023 | Yang, Shuai; Shi, Weili; Yan, Wenqiang; Ao, Yingfang; Guo, Qinwei; Yang, Yuping | Comparison between primary repair and augmented repair with gastrocnemius turn-down flap for acute Achilles tendon rupture: a retrospective study with minimum 2-year follow-up. | BMC musculoskeletal disorders | 7 | 2015 | 68 | 5.9 |
| 2023 | Hammad, Mostafa Elsebai; Fayed, Aly Maher; Ayoub, Mostafa Ahmed; Emran, Ali Mahmoud | Early satisfactory results of percutaneous repair in neglected achilles tendon rupture. | BMC musculoskeletal disorders | 7 | 2016 | 24 | 29.2 |
| 2023 | McCormick, Brian P.; Trent, Sarah; Haislup, Brett D.; Bolster, Drew; Bubnash, Kimberly; Miller, Stuart D. | Dual Semitendinosus Allograft Reconstruction of Chronic Achilles Tendon Ruptures: Operative Technique and Outcomes. | Foot & ankle international | 9 | 2016 | 9 | 33.3 |
| 2023 | Xu, Tonglong; Liu, Xuanzhe; Tian, Jian; Liu, Shen; Mi, Jingyi; Xu, Yajun; Chen, Xueming; Zhang, Yuxuan | Endoscopic-assisted locking block modified Krackow technique combined with a V-Y flap for chronic Achilles tendon rupture. | Knee surgery, sports traumatology, arthroscopy : official journal of the ESSKA | 7 | 2016 | 29 | 0.0 |
| 2023 | Campillo-Recio, D.; Comas-Aguilar, M.; Ibáñez, M.; Maldonado-Sotoca, Y.; Albertí-Fitó, G. | Percutaneous Achilles tendon repair with absorbable suture: Outcomes and complications. | Revista espanola de cirugia ortopedica y traumatologia | 4 | 2017 | 52 | 7.7 |
| 2023 | Ge, Laurence; Saunders, Noah; Betts, Dakota; Holmes, James R.; Walton, David M.; Talusan, Paul G. | Midterm Outcomes of Operatively and Nonoperatively Managed Achilles Tendon Ruptures in Young Adults. | Foot & ankle orthopaedics | 8 | 2017 | 42 | 45.2 |
| 2023 | Maffulli, Nicola; Gougoulias, Nikolaos; Christidis, Panagiotis; Maffulli, Gayle D.; Oliva, Francesco | Primary augmentation of percutaneous repair with flexor hallucis longus tendon for Achilles tendon ruptures reduces tendon elongation and may improve functional outcome. | Knee surgery, sports traumatology, arthroscopy : official journal of the ESSKA | 5 | 2017 | 62 | 24.2 |
| 2023 | Poszepczyński, Jan; Pietrusiński, Michał; Borowiec, Maciej; Edward Domżalski, Marcin | Assessment of fibrillin-2 and elastin gene polymorphisms in patients with a traumatic Achilles tendon rupture: Is Achilles tendon rupture a genetic disease? | Acta orthopaedica et traumatologica turcica | 5 | 2017 | 106 | 12.3 |
| 2023 | Bronheim, Rachel S.; Shu, Henry T.; Jami, Meghana; Hsu, Nigel N.; Aiyer, Amiethab A. | Surgical Setting in Achilles Tendon Repair: How Does It Relate to Costs and Complications? | Foot & ankle orthopaedics | 7 | 2018 | 97 | 21.6 |
| 2023 | Hansen, Maria Swennergren; Bencke, Jesper; Kristensen, Morten Tange; Kallemose, Thomas; Hölmich, Per; Barfod, Kristoffer Weisskirchner | Achilles tendon gait dynamics after rupture: A three-armed randomized controlled trial comparing an individualized treatment algorithm vs. operative or non-operative treatment. | Foot and ankle surgery : official journal of the European Society of Foot and Ankle Surgeons | 2 | 2018 | 60 | 20.0 |
| 2023 | Lee, Conrad; Haarer, Francesca; Titheradge, Rachel; Iliopoulos, Efthymios | Thromboembolic Events During Weightbearing vs Nonweightbearing Accelerated Rehabilitation Protocols for Complete Achilles Tendon Ruptures. | Foot & ankle orthopaedics | 4 | 2018 | 305 | 31.5 |
| 2023 | Mashimo, Shota; Nozaki, Taiki; Amaha, Kentaro; Tanaka, Keita; Kubota, Junya; Sato, Hiroyuki; Kitamura, Nobuto | Quantitative Assessment of Calf Muscle Volume, Strength, and Quality After Achilles Tendon Rupture Repair: A 1-Year Prospective Follow-up Study. | The American journal of sports medicine | 4 | 2018 | 20 | 25.0 |
| 2023 | Park, Young Hwan; Kim, Woon; Choi, Jung Woo; Kim, Hak Jun | Ultrasonographic Finding of Contralateral Achilles Tendon in Patients With Acute Achilles Tendon Rupture: A Prospective Observational Study. | Clinical journal of sport medicine : official journal of the Canadian Academy of Sport Medicine | 3 | 2018 | 75 | 8.0 |
| 2023 | Reingrittha, Pissanu; Benjawongsathien, Kriangkamol; Visuthisakchai, Surawit | The Efficacy of Posterior Fasciotomy Versus Inserted Vacuum Drainage in Reducing Postoperative Surgical Site Infection in Open Achilles Tendon Repair: A Prospective Cohort Study With Inverse Probability Treatment Weight Propensity Score Analysis. | The Journal of foot and ankle surgery : official publication of the American College of Foot and Ankle Surgeons | 4 | 2018 | 60 | 30.0 |
| 2023 | Saab, Marc; Derousseaux, Romain; Beldame, Julien; Chantelot, Christophe; Laboute, Eric; Maynou, Carlos | Portable device for measuring isometric plantar-flexion force after open Achilles repair: Retrospective cohort of 30 recreational athletes with one-year minimum follow-up. | Orthopaedics & traumatology, surgery & research : OTSR | 4 | 2018 | 30 | 10.0 |
| 2023 | Wu, Chen-Xi; Xiong, Chang-Yue; Bai, Lu; Chen, Su-Meng; Yan, Yu-Xin; Wang, Lu; Zhang, Xin-Tao | Achilles tendon thickening does not affect elasticity and functional outcome after surgical repair of Achilles rupture: A retrospective study. | Chinese journal of traumatology = Zhonghua chuang shang za zhi | 5 | 2018 | 84 | 4.8 |
| 2023 | Yoshida, Keiichi; Itoigawa, Yoshiaki; Morikawa, Daichi; Maruyama, Yuichiro; Ishijima, Muneaki | Chronologic Changes in the Elastic Modulus of a Healing Achilles Tendon Rupture Measured Using Shear Wave Elastography. | Foot & ankle international | 6 | 2018 | 55 | 23.6 |
| 2023 | Bi, Andrew S.; Azam, Mohammad T.; Butler, James J.; Alaia, Michael J.; Jazrawi, Laith M.; Gonzalez-Lomas, Guillem; Kennedy, John G. | Increased incidence of acute achilles tendon ruptures in the peri-pandemic COVID era with parallels to the 2021-22 NFL season. | Knee surgery, sports traumatology, arthroscopy : official journal of the ESSKA | 5 | 2019 | 588 | 24.3 |
| 2023 | Cramer, Allan; Højfeldt, Grith; Schjerling, Peter; Agergaard, Jakob; van Hall, Gerrit; Olsen, Jesper; Hölmich, Per; Kjaer, Michael; Barfod, Kristoffer Weisskirchner | Achilles Tendon Tissue Turnover Before and Immediately After an Acute Rupture. | American Journal of Sports Medicine | 2 | 2019 | 18 | 33.3 |
| 2023 | Deng, Zhibo; Li, Zhi; Shen, Chen; Sun, Xianding; Wang, Ting; Nie, Mao; Tang, Kaiying | Outcomes of early versus late functional weight-bearing after the acute Achilles tendon rupture repair with minimally invasive surgery: a randomized controlled trial. | Archives of orthopaedic and trauma surgery | 4 | 2019 | 68 | 14.7 |
| 2023 | Schneebeli, Alessandro; Barbero, Marco; Filardo, Giuseppe; Testa, Enrique; Riegger, Martin; Sangiorgio, Alessandro; Cescon, Corrado; Soldini, Emiliano; Falla, Deborah | Shear Wave Tensiometry Can Detect Loading Differences Between Operated and Unaffected Achilles Tendon. | Foot & ankle international | 4 | 2019 | 21 | 0.0 |
| 2023 | Subaşı, İzzet Özay; Çepni, Şahin; Tanoğlu, Oğuzhan; Veizi, Enejd; Alkan, Hilmi; Yapici, Furkan; Firat, Ahmet | A clinical comparison of two different surgical techniques in the treatment of acute Achilles tendon ruptures: Limited-open approach vs. percutaneous approach. | Ulusal travma ve acil cerrahi dergisi = Turkish journal of trauma & emergency surgery : TJTES | 2 | 2019 | 53 | 35.8 |
| 2023 | Wu, Helin; Dong, Jingxian; Dong, Dandan; Wei, Shijun; Zheng, Boyu; Kong, Changwang; Xu, Feng; Hou, Wenguang | Correlation between the Coaptation and Regeneration of Tendon Stumps in Endoscopic Assisted Achilles Tendon Rupture Repair. | Orthopaedic surgery | 3 | 2019 | 46 | 2.2 |
| 2023 | Xu, Lihu; Jin, Jiaxin; Liu, Zhongcheng; Wu, Meng; Peng, Bo; Jiang, Jin; Liu, Guangyao; He, Jinwen; White, Sylvia; Xia, Yayi | A New Technique of Achilles Tendon Rupture Repaired by Double Transverse Mini-incision to Avoid Sural Nerve Injury: A Consecutive Retrospective Study. | Orthopaedic surgery | 2 | 2019 | 20 | 5.0 |
| 2023 | Bashir, Arshad; Parry, Mudasir Ahmad; Bhat, Ajaz Ahmad | Functional Outcome in Percutaneous Achilles Tendon Repair. | Indian journal of orthopaedics | 3 | 2020 | 25 | 28.0 |
| 2023 | Laboute, E.; Thoreux, P.; Beldame, J.; Caubere, A.; Giunta, J. C.; Coursier, R.; Saab, M. | Re-ruptures and early outcomes after surgical repair of acute Achilles tendon ruptures: prospective, comparative multicenter study. | International orthopaedics | 2 | 2020 | 112 | 19.6 |
| 2023 | Ramakanth, Rajagopalakrishnan; Sundararajan, Silvampatti Ramasamy; Thippeswamy, Venugopal; D Souza, Terence; Palanisamy, Arumugam; Rajasekaran, Shanmuganathan | "Foot peak pressures are comparable to normal foot after flexor hallucis longus transfer for chronic retracted tendo-achilles tear: A pedobarographic analysis of normal foot versus affected foot". | Journal of ISAKOS : joint disorders & orthopaedic sports medicine | 4 | 2020 | 46 | 37.0 |
| 2023 | Venkateshaiah, S; Reddy, AGR; Srikantaiah, VC | Transfer of the Flexor Hallucis Longus Tendon for Neglected and Degenerative Tendo Achillis Rupture: A Prospective Clinical Study | MLTJ-MUSCLES LIGAMENTS AND TENDONS JOURNAL | 3 | 2020 | 22 | 36.4 |
| 2023 | Wang, Zhuang; Chen, Weiwei; Jia, Honglei; Hu, Fangning; Wang, Bomin; Yang, Yongliang; Liu, Fanxiao | Application of Preoperative Ultrasonography in the Percutaneous Minimally Invasive Repair of Acute Closed Achilles Tendon Rupture. | BioMed research international | 1 | 2020 | 16 | 18.8 |
| 2023 | Shitrit, E.; Valentin, E.; Baudrier, N.; Bohu, Y.; Rauline, G.; Lopes, R.; Bauer, T.; Hardy, A. | The ALR-RSI score can be used to evaluate psychological readiness to return to sport after acute Achilles tendon tear. | Knee surgery, sports traumatology, arthroscopy : official journal of the ESSKA | 2 | 2021 | 50 | 16.0 |
| 2024 | Yüce, Ali; Yerli, Mustafa; Misir, Abdulhamit | The Injury Mechanism of Achilles Tendon Rupture in Professional Athletes: A Video Analysis Study. | Journal of the American Podiatric Medical Association | 51 | 1995 | 42 | 2.4 |
| 2024 | Weinberg, David; Shofer, Frances; Pan, Jason | Performance Differences in Elite National Basketball Association and Women's National Basketball Association Players Based Upon Whether the Dominant or Non-dominant Achilles Tendon Was Ruptured. | Cureus | 31 | 2005 | 18 | 22.2 |
| 2024 | Ifarraguerri, Anna M.; Quan, Theodore; Farley, Benjamin; Kuyl, Emile-Victor; Koch, John; Parel, Philip M.; Malyavko, Alisa; Tabaie, Sean | The effect of smoking on 30-day complications following primary repair of Achilles tendon ruptures. | European journal of orthopaedic surgery & traumatology : orthopedie traumatologie | 14 | 2012 | 4209 | 25.8 |
| 2024 | Brodeur, Peter G.; Salameh, Motasem; Boulos, Alexandre; Blankenhorn, Brad D.; Hsu, Raymond Y. | Surgical Management of Achilles Tendon Ruptures in the United States 2006-2020, an ABOS Part II Oral Examination Case List Database Study. | Foot & ankle orthopaedics | 15 | 2013 | 6677 | 20.2 |
| 2024 | Choi, Yoon H.; Kwon, Tae H.; Choi, Ji H.; Han, Hee S.; Lee, Kyoung M. | Factors associated with Achilles tendon re-rupture following operative fixation. | Bone & joint research | 11 | 2013 | 43287 | 19.2 |
| 2024 | Mzeihem, Majd; El Bachour, Joseph; Hemdanieh, Maya; El Baba, Bachar; Tamim, Hani; Nassereddine, Mohamad | Achilles tendon rupture primary repair technique: A comparative retrospective study between graft versus no-graft. | Journal of clinical orthopaedics and trauma | 17 | 2013 | 7010 | 25.5 |
| 2024 | Huang, Xin; Liu, Jia-Wei; Jiang, Yu; Zhu, Hong-Wei; Hu, Xing-Xing; Wu, Ke-Jian; Wang, Xiao-Ning; Zhang, Shuai | Peritendinous Submembrane Access Technique for Management of Acute Ruptures of the Achilles Tendon: A Retrospective Study of 249 Cases. | Orthopaedic surgery | 11 | 2014 | 249 | 4.0 |
| 2024 | Choi, Jun Young; Choo, Suk Kyu; Kim, Byung Ho; Suh, Jin Soo | Conservative treatment outcome for Achilles tendon re-rupture occurring in the subacute phase after primary repair. | Archives of orthopaedic and trauma surgery | 12 | 2015 | 385 | 15.3 |
| 2024 | Mitrogiannis, Leonidas; Mitrogiannis, George; Melaki, Kalliopi; Karamitros, Athanasios; Karantanas, Apostolos; Paxinos, Odysseas | An Early Functional Unsupervised Rehabilitation Protocol Allows Safe Return to Function After Achilles Tendon Repair. | Cureus | 6 | 2015 | 25 | 4.0 |
| 2024 | Arbab, Dorothe; Lichte, Philip; Gutteck, Natalia; Bouillon, Bertil; Arbab, Dariusch | Reliability and validation of the German Achilles tendon Total Rupture Score. | Knee surgery, sports traumatology, arthroscopy : official journal of the ESSKA | 11 | 2016 | 48 | 16.7 |
| 2024 | Carmont, Michael R.; Gunnarsson, Baldvin; Brorsson, Annelie; Nilsson-Helander, Katarina | Musculotendinous ruptures of the achilles tendon had greater heel-rise height index compared with mid-substance rupture with non-operative management: A retrospective cohort study. | Journal of ISAKOS : joint disorders & orthopaedic sports medicine | 15 | 2016 | 24 | 29.2 |
| 2024 | Hartman, Hayden; Cacace, Alexis; Leatherman, Hadley; Ashkani-Esfahani, Soheil; Guss, Daniel; Waryasz, Gregory; DiGiovanni, Christopher W.; Gianakos, Arianna L. | Gender Differences in Achilles Tendon Ruptures-A Retrospective Study and a Review of the Literature. | The Journal of foot and ankle surgery : official publication of the American College of Foot and Ankle Surgeons | 11 | 2016 | 863 | 18.3 |
| 2024 | Zhu, Sophie; Garofalo, Josh; Abuhantash, Monther; McRae, Sheila; MacDonald, Peter; Longstaffe, Rob; Ogborn, Dan | Functional Performance and Tendon Morphology After Operative or Nonoperative Treatment of Achilles Tendon Ruptures. | International journal of sports physical therapy | 6 | 2016 | 24 | 29.2 |
| 2024 | Jiang, Yi; Wang, Yuchao; Liu, Bokai; Zhao, Yilin; He, Qingquan; Wu, Kanglong | Hyperuricemia May Increase Risk of Achilles Tendon Rupture: A Case Control Study. | The Journal of foot and ankle surgery : official publication of the American College of Foot and Ankle Surgeons | 10 | 2017 | 257 | 6.6 |
| 2024 | Briggs-Price, Samuel; Mangwani, Jitendra; Houchen-Wolloff, Linzy; Modha, Gayatri; Fitzpatrick, Emma; Faizi, Murtaza; Shepherd, Jenna; O'Neill, Seth | Incidence, demographics, characteristics and management of acute Achilles tendon rupture: An epidemiological study. | PloS one | 7 | 2018 | 361 | 20.8 |
| 2024 | Hendriks, Joris R. H.; Baker, Riley J.; de Groot, Tom M.; Lans, Amanda; Waryasz, Gregory R.; Kerkhoffs, Gino M. M. J.; Ashkani-Esfahani, Soheil; DiGiovanni, Christopher W.; Guss, Daniel | The Influence of Patient Characteristics and Social Determinants of Health on Postoperative Complications Following Achilles Tendon Rupture. | Foot & ankle international | 7 | 2018 | 521 | 17.5 |
| 2024 | Saab, Marc; Beldame, Julien; Charpail, Christel; Kaba, Arnaud; Mainard, Didier; Caubère, Alexandre; Maynou, Carlos; Bredicianu, Rares; Ghorbani, Ali; Giunta, Jean-Charles; Coursier, Raphaël; Thoreux, Patricia; Laboute, Eric | Clinical and functional outcomes of 405 Achilles tendon ruptures after a minimum follow-up of 1 year. | Orthopaedics & traumatology, surgery & research : OTSR | 2 | 2018 | 405 | 25.7 |
| 2024 | Savage-Elliott, Ian; Li, Zachary I.; Moore, Michael R.; Lezak, Bradley; Jazrawi, Taylor; Golant, Alexander; Meislin, Robert J. | No difference in operative time, outcomes, cosmesis, or return to activity and/or sport after minimally invasive versus open repair of primary Achilles ruptures: a retrospective review. | European journal of orthopaedic surgery & traumatology : orthopedie traumatologie | 4 | 2018 | 63 | 20.6 |
| 2024 | Wagers, Kade; Ofori-Atta, Blessing S.; Tucker, William; Presson, Angela P.; Nixon, Devon | Evaluation of Costs Associated With Acute Achilles Tendon Repair. | Foot & ankle orthopaedics | 9 | 2018 | 224 | 18.8 |
| 2024 | Xue, Han; Xiao, Fengxu; Li, Ruochen; Wu, Guangwei; Zhu, Zheyue; Zhang, Chen; Li, Miao | Transversal calcaneal anchored Achilles tendon reconstruction with free semitendinosus tendon autograft for acute rupture of Achilles tendon: clinical evaluation. | Scientific reports | 6 | 2018 | 17 | 29.4 |
| 2024 | Christensen, Marianne; Silbernagel, Karin Grävare; Zellers, Jennifer A.; Kjær, Inge Lunding; Rathleff, Michael Skovdal | Feasibility of an early progressive resistance exercise program for acute Achilles tendon rupture. | Pilot and feasibility studies | 2 | 2019 | 16 | 18.8 |
| 2024 | Jin, Wen Tao; Huang, Li Fang; Guo, Hai Hua; Wang, Lei; Li, Xiang; Wang, Ze Jin | Two mini transverse-incision repair yields better results than percutaneous repair for acute closed midsubstance Achilles tendon rupture: a retrospective case-control study. | Journal of orthopaedic surgery and research | 4 | 2019 | 60 | 1.7 |
| 2024 | Hoeffner, Rikke; Agergaard, Anne-Sofie; Svensson, Rene B.; Cullum, Camilla; Mikkelsen, Rasmus Kramer; Konradsen, Lars; Krogsgaard, Michael; Boesen, Mikael; Kjaer, Michael; Magnusson, S. Peter | Tendon Elongation and Function After Delayed or Standard Loading of Surgically Repaired Achilles Tendon Ruptures: A Randomized Controlled Trial. | The American journal of sports medicine | 2 | 2020 | 48 | 27.1 |
| 2024 | Karlidag, Taner; Bingol, Olgun; Kulakoglu, Burak; Keskin, Omer Halit; Durgal, Atahan; Ozdemir, Guzelali | Effects of tendon elongation on plantar pressure and clinical outcomes: A comparative analysis between open repair and minimally invasive surgery. | Knee surgery, sports traumatology, arthroscopy : official journal of the ESSKA | 3 | 2020 | 65 | 12.3 |
| 2024 | Li, Chao; Li, Fu-Chun | Modified Minimally Invasive Bunnell Suture Surgery with Channel-assisted Minimally Invasive Reconstruction Device for Treating Achilles Tendon Rupture. | Zeitschrift fur Orthopadie und Unfallchirurgie | 3 | 2020 | 20 | 10.0 |
| 2024 | Nilsson, Niklas; Alim, M.D. Abdul; Dietrich-Zagonel, Franciele; Concaro, Sebastian; Brorsson, Annelie; Nilsson Helander, Katarina; Eliasson, Pernilla | The Delayed Presentation of Achilles Tendon Ruptures Is Associated With Marked Alterations in the Gene Expression of COL1A1, MMPs, TIMPs, and IL-6. | American Journal of Sports Medicine | 2 | 2020 | 27 | 37.0 |
| 2024 | Sukanen, Maria; Khair, Ra'ad M.; Reito, Aleksi; Ponkilainen, Ville; Paloneva, Juha; Cronin, Neil; Hautala, Arto J.; Finni, Taija | Early Predictors of Recovery From Nonoperatively Treated Achilles Tendon Rupture: 1 Year Follow-Up Study. | Scandinavian journal of medicine & science in sports | 5 | 2020 | 35 | 20.0 |
| 2024 | Wei, Shijun; Li, Qing; Wu, Helin; Kong, Changwang; Xu, Feng; Cai, Xianhua | All-inside endoscopic semiautomatic running locked stitch technique shows favourable outcomes for acute Achilles tendon ruptures. | Knee surgery, sports traumatology, arthroscopy : official journal of the ESSKA | 2 | 2020 | 40 | 5.0 |
| 2024 | Ibrahim, Mohamed A. A.; Abdelkader, Mohamed G.; Nematallah, Samir A.; Elsawy, Gamal A.; Alghandour, Sameh A.; Shwitter, Lotfy M. | Modified gastro-soleus turn-down flap for chronic or neglected achilles tendon ruptures. | Journal of orthopaedic surgery and research | 4 | 2021 | 18 | 27.8 |
| 2024 | Larsson, Elin; Nilsson, Niklas; Walstern, Julia; Brorsson, Annelie; Helander, Katarina Nilsson | Females present larger deficit in heel-rise height at 3 months following an Achilles tendon rupture compared with males. | Knee surgery, sports traumatology, arthroscopy : official journal of the ESSKA | 3 | 2021 | 127 | 19.7 |
| 2024 | Zhao, Yanrui; Wang, Hanzhou; Zhao, Binzhi; Diao, Shuo; Gao, Yuling; Zhou, Junlin; Liu, Yang | The learning curve for minimally invasive Achilles repair using the "lumbar puncture needle and oval forceps" technique. | BMC musculoskeletal disorders | 3 | 2021 | 36 | 19.4 |
| 2024 | Bentzen, Andreas; Jørgensen, Stian Langgård; Birch, Sara; Mortensen, Louise; Toft, Marianne; Lindvig, Michael Godsvig; Gundtoft, Per Hviid; Mechlenburg, Inger | Feasibility of Blood Flow Restriction Exercise in Adults with a Non-surgically Treated Achilles Tendon Rupture; a Case Series. | International journal of exercise science | 2 | 2022 | 18 | 22.2 |
