# Supplementary material for: Changes in sex distribution in Achilles tendon rupture literature over 74 years: A systematic review
Source: Knee Surg Sports Traumatol Arthrosc. 2026 Jul 6;34(8):2978–86. doi: 10.1002/ksa.70520 (PMC13359244; doi:10.1002/ksa.70520)
Supplement: Supplementary file 3 — Supporting File 3. [file KSA-34-2978-s001.docx]

Changes in Sex Distribution in Achilles Tendon Rupture Literature Over 74 Years: A Systematic Review

KSSTA

Authors: Potter MN, Mlecko J, Christensen M, Aufwerber S, Katz SE, Pohlig RT, Silbernagel SG.

**Supplemental Table 3**. Number of articles grouped by midpoint of enrollment year, and the summed sample size across articles for that year.

| **Midpoint Year** | **Number of Articles** | **Cumulative Sample Size Across Articles** | **Cumulative Female Sample Size** | **Cumulative Male Sample Size** | **Percentage of Females** | **Percentage of**  **Males** |
| --- | --- | --- | --- | --- | --- | --- |
| 1948 | 1 | 92 | 13 | 79 | 14.1 | 85.9 |
| 1958 | 1 | 102 | 19 | 83 | 18.6 | 81.4 |
| 1961 | 1 | 229 | 29 | 200 | 12.7 | 87.3 |
| 1964 | 1 | 37 | 3 | 34 | 8.1 | 91.9 |
| 1966 | 1 | 6 | 1 | 5 | 16.7 | 83.3 |
| 1973 | 1 | 68 | 11 | 57 | 16.2 | 83.8 |
| 1974 | 2 | 29 | 3 | 26 | 10.3 | 89.7 |
| 1975 | 1 | 73 | 22 | 51 | 30.1 | 69.9 |
| 1977 | 1 | 8 | 2 | 6 | 25.0 | 75.0 |
| 1978 | 2 | 59 | 10 | 49 | 16.9 | 83.1 |
| 1980 | 2 | 47 | 10 | 37 | 21.3 | 78.7 |
| 1982 | 3 | 76 | 28 | 48 | 36.8 | 63.2 |
| 1983 | 3 | 66 | 14 | 52 | 21.2 | 78.8 |
| 1984 | 1 | 66 | 12 | 54 | 18.2 | 81.8 |
| 1985 | 8 | 396 | 80 | 316 | 20.2 | 79.8 |
| 1986 | 7 | 631 | 121 | 510 | 19.2 | 80.8 |
| 1987 | 2 | 4,221 | 1,573 | 2,648 | 37.3 | 62.7 |
| 1988 | 2 | 30 | 7 | 23 | 23.3 | 76.7 |
| 1989 | 8 | 755 | 115 | 640 | 15.2 | 84.8 |
| 1990 | 3 | 108 | 8 | 100 | 7.4 | 92.6 |
| 1991 | 7 | 299 | 66 | 233 | 22.1 | 77.9 |
| 1992 | 3 | 117 | 13 | 104 | 11.1 | 88.9 |
| 1993 | 9 | 2,201 | 556 | 1,645 | 25.3 | 74.7 |
| 1994 | 7 | 505 | 52 | 453 | 10.3 | 89.7 |
| 1995 | 10 | 1,269 | 261 | 1,008 | 20.6 | 79.4 |
| 1996 | 6 | 1,925 | 436 | 1,489 | 22.6 | 77.4 |
| 1997 | 6 | 7,901 | 2,503 | 5,398 | 31.7 | 68.3 |
| 1998 | 13 | 2,533 | 440 | 2,093 | 17.4 | 82.6 |
| 1999 | 10 | 1,396 | 344 | 1,052 | 24.6 | 75.4 |
| 2000 | 12 | 971 | 177 | 794 | 18.2 | 81.8 |
| 2001 | 8 | 1051 | 181 | 870 | 17.2 | 82.8 |
| 2002 | 21 | 1,914 | 458 | 1,456 | 23.9 | 76.1 |
| 2003 | 14 | 34,676 | 8,556 | 26,120 | 24.7 | 75.3 |
| 2004 | 14 | 1,485 | 249 | 1,236 | 16.8 | 83.2 |
| 2005 | 15 | 786 | 149 | 637 | 19.0 | 81.0 |
| 2006 | 15 | 29,344 | 6,317 | 23,027 | 21.5 | 78.5 |
| 2007 | 16 | 864 | 122 | 742 | 14.1 | 85.9 |
| 2008 | 17 | 85,647 | 27,838 | 57,809 | 32.5 | 67.5 |
| 2009 | 22 | 3,036 | 658 | 2,379 | 21.7 | 78.4 |
| 2010 | 22 | 1,040 | 193 | 847 | 18.6 | 81.4 |
| 2011 | 20 | 54,982 | 17,363 | 37,619 | 31.6 | 68.4 |
| 2012 | 26 | 6,199 | 1,443 | 4,756 | 23.3 | 76.7 |
| 2013 | 25 | 202,099 | 57,197 | 143,976 | 28.3 | 71.2 |
| 2014 | 22 | 2,451 | 438 | 2,013 | 17.9 | 82.1 |
| 2015 | 34 | 105,220 | 40,503 | 64,717 | 38.5 | 61.5 |
| 2016 | 27 | 2,407 | 387 | 2,020 | 16.1 | 83.9 |
| 2017 | 22 | 2,957 | 515 | 2,442 | 17.4 | 82.6 |
| 2018 | 27 | 2,846 | 607 | 2,239 | 21.3 | 78.7 |
| 2019 | 13 | 986 | 201 | 785 | 20.4 | 79.6 |
| 2020 | 13 | 763 | 158 | 605 | 20.7 | 79.3 |
| 2021 | 4 | 231 | 45 | 186 | 19.5 | 80.5 |
| 2022 | 1 | 18 | 4 | 14 | 22.2 | 77.8 |
